# DNABP: Identification of DNA-Binding Proteins Based on Feature Selection Using a Random Forest and Predicting Binding Residues

**DOI:** 10.1371/journal.pone.0167345

**Published:** 2016-12-01

**Authors:** Xin Ma, Jing Guo, Xiao Sun

**Affiliations:** 1 School of Science, Nanjing Audit University, Nanjing, China; 2 State Key Laboratory of Bioelectronics, School of Biological Science and Medical Engineering, Southeast University, Nanjing, China; Harbin Institute of Technology Shenzhen Graduate School, CHINA

## Abstract

DNA-binding proteins are fundamentally important in cellular processes. Several computational-based methods have been developed to improve the prediction of DNA-binding proteins in previous years. However, insufficient work has been done on the prediction of DNA-binding proteins from protein sequence information. In this paper, a novel predictor, DNABP (DNA-binding proteins), was designed to predict DNA-binding proteins using the random forest (RF) classifier with a hybrid feature. The hybrid feature contains two types of novel sequence features, which reflect information about the conservation of physicochemical properties of the amino acids, and the binding propensity of DNA-binding residues and non-binding propensities of non-binding residues. The comparisons with each feature demonstrated that these two novel features contributed most to the improvement in predictive ability. Furthermore, to improve the prediction performance of the DNABP model, feature selection using the minimum redundancy maximum relevance (mRMR) method combined with incremental feature selection (IFS) was carried out during the model construction. The results showed that the DNABP model could achieve 86.90% accuracy, 83.76% sensitivity, 90.03% specificity and a Matthews correlation coefficient of 0.727. High prediction accuracy and performance comparisons with previous research suggested that DNABP could be a useful approach to identify DNA-binding proteins from sequence information. The DNABP web server system is freely available at http://www.cbi.seu.edu.cn/DNABP/.

## Introduction

DNA-protein interactions play significant roles in various biological processes, such as gene regulation, DNA replication and repair, transcription and other biological activities associated with DNA [1–3]. Identification of DNA-binding proteins is fundamentally important to understand how proteins interact with DNA. DNA-binding proteins can be identified by many experimental techniques such as chromatin immunoprecipitation on microarrays, X-ray crystallography and nuclear magnetic resonance (NMR). However, the experimental techniques to recognize DNA-binding proteins are labor-intensive and time-consuming. Considering the weakness of determination of DNA-binding proteins using wet experiments, computational methods to identify putative DNA-binding proteins have become increasingly important in recent years. In recent years, rapid advances in genomic and proteomic techniques have generated numerous DNA-binding protein sequences. In 2014, the number of DNA-binding proteins in the UniProt database was more than 10 times greater than that in 2000. These large amounts of data provide the foundation for research on the identification of DNA-binding proteins using computational approaches.

Currently, there are two major tasks for the computational prediction of DNA-binding proteins. One is to identify DNA-binding proteins using structure information and the other is to predict them using sequence information. Obtaining structural information is difficult; therefore, it is necessary to develop prediction methods for DNA-binding proteins based on amino acid sequences.

During the past few decades, a series of studies on the identification of DNA-binding proteins using sequence information have been published [4–14]. Machine learning algorithms were employed to construct models to predict DNA-binding proteins and produced effective performances [4–9,11–19]. Interestingly, the support vector machine (SVM) algorithm has been used frequently to predict DNA-binding proteins [4–6,8,12–16]. Cai and Lin first applied the SVM algorithm for DNA-binding protein prediction using a protein’s amino acid composition and a limited range of correlations of hydrophobicity and solvent-accessible surface areas as input features [4]. More recently, Zou et al. developed an entirely sequence-based protocol that transforms and integrates informative features from different scales used by SVM to predict DNA-binding proteins [14]. Zhang et al. proposed newDNA-Prot, a DNA-binding protein predictor that employs an SVM classifier and a comprehensive feature that categorized features into six groups: primary sequence-based, evolutionary profile-based, predicted secondary structure-based, predicted relative solvent accessibility-based, physicochemical property-based and biological function-based features [13]. DNA-Pro based on SVM algorithm to distinguish DNA-binding proteins from non-binding proteins [17]. They incorporated features of overall amino acid composition, pseudo amino acid composition (PseAAC) proposed by Chou and physicochemical distance transformation. Liu et al. proposed a predictor called iDNAPro-PseAAC [18] which used PseAAC feature Combined with SVM algorithm. The most recent prediction method for DNA-binding proteins was aslo proposed Liu et al.which called iDNA-KACC[19]. The iDNA-KACC was developed by combing SVM classifier as well as by incorporating the auto-cross covariance transformation. The protein sequences are first converted into profile-based protein representation, and then converted into a series of fixed-length vectors by the auto-cross covariance transformation with Kmer composition. Random forest (RF) alorgithm, which is a useful machine learning classifier, was aslo used to prdict DNA-binding proteins. Lou et al. applied the RF algorithm to predict DNA-binding proteins using predicted secondary structure, predicted relative solvent accessibility and position-specific scoring matrix as the primary sequence features[8].

In this study, a systematic attempt was made to develop models to predict DNA-binding proteins with high accuracy using only sequence information. DNA-binding proteins have DNA-binding residues and non-binding proteins should not have DNA-binding residues. Therefore, the presence of DNA-binding residues could be used to predict DNA-binding proteins. We established an effective model, DNABR [20], to predict DNA-binding residues. The information of DNA-binding residues and non-binding residues predicted by DNABR was constructed as a feature vector to classify DNA-binding proteins and non-binding proteins. In addition, we proposed a novel feature, PSSM-PP, based on a position-specific scoring matrix (PSSM). The PSSM-PP feature not only represents the evolutionary information obtained by PSSM, but also contains information about physicochemical properties. Thus the novel method DNABP uses a random forest (RF) algorithm [21] in conjunction with a hybrid feature. The hybrid feature comprises 64 features selected from the PSSM-PP, DNA-binding propensity measures obtained from the information of DNA-binding residues, non-binding propensity measures obtained from the information of non-binding residues and physicochemical property features using the minimum redundancy maximum relevance (mRMR) method combined with incremental feature selection (IFS). Since user-friendly and publicly accessible web-servers represent the future direction for developing practically more useful models, simulated methods, or predictors as pointed out in [22–24] and emphasized in [25,26], we have established a web-server presented in this paper.

## Materials and Methods

### Dataset

All DNA-binding protein sequences and non-binding protein sequences were collected from the UniProt database (http://www.uniprot.org/) [27] and only manually annotated and reviewed proteins were selected for this study.

To obtain the DNA-binding proteins as the positive dataset, “DNA binding” was used as keyword to search the UniProt database. More than 30000 DNA-binding proteins were obtained. As in previous works [4,6,9,12,28], we removed proteins with lengths less than 50 amino acids because they might be fragments and proteins of more than 6000 amino acids because they might be protein complexes. Protein sequences including irregular amino acid characters such as “x” and “z” were also removed. To avoid any effects on our experimental data from the similarity of the dataset, we removed any redundant data using the BLAST package [29] available from NCBI, with a threshold of 40%. Finally, our positive dataset had 7131 DNA-binding protein sequences.

To obtain the non-binding proteins as the negative dataset, we first selected all of the proteins from the UniProt database that did not have an implied RNA/DNA-binding functionality using a similar procedure to that proposed by Cai and Lin [4]. In total, 528,086 non-binding proteins were processed according to the similarity criteria as the negative dataset. Consequently, we selected 67029 non-binding protein sequences as the negative dataset. An equal number of positive data and negative data is important to develop the prediction system for DNA-binding proteins. However, the number of DNA-binding proteins in the positive dataset was much less than the number of non-binding proteins in the negative dataset. The imbalance between the positive and negative data would affect the prediction performance; therefore, we randomly selected 7131 non-binding proteins from the negative dataset to balance with the positive dataset. The main dataset (Mainset) then comprised the 7131 DNA-binding proteins in the positive dataset and the selected 7131 non-binding proteins in the negative dataset (See Additional file S1 Table).

We further divided the 14262 proteins in the main dataset into two datasets: 1) the training dataset (Trainset), which comprised 6928 DNA-binding proteins and 6928 non-binding proteins (total 13856); 2) an independent test dataset (Testset), which consisted of 203 DNA-binding proteins and 203 non-binding proteins (total 406). The independent test dataset was used to evaluate the performance of our method against previous works [7,9,11]. Therefore, the proteins in Testset did not include any proteins that were used in previous works [7,9,11].

### Feature vector

#### Position-specific scoring matrix combined with physicochemical properties (PSSM-PP)

The PSSM, which represents evolutionary information of amino acid sequences, has been used widely in research on the prediction of DNA-binding residues [30–35] and DNA-binding proteins [6,14,28] based on sequence information. Compared with other features, PSSM contributes most to improving the prediction performance of DNA-binding residues and DNA-binding proteins.

The PSSM scores used in this work were generated by PSI-BLAST [29]. PSI-BLAST searches for each amino acid sequences were carried out against the non-redundant dataset of proteins in NCBI with an E value of 0.001. The 20 values of PSSM, obtained for each sequence position, were then scaled to the range of 0–1 using the following formula:

$$f(x)={\left[1+\exp(-x)\right]}^{-1}$$ (1)

where *x* is the element value of the PSSM profile.

The PSSM feature for different proteins has a different vector dimension. Taking a query protein with *N* amino acids as an example, the vector dimension of the PSSM feature is 20*N. Considering the fact that the machine learning model construction requires a fixed vector dimension, the variable vector dimension of PSSM feature should be converted into a fixed dimension.

Furthermore, to improve the PSSM feature, we considered a physicochemical property feature combined with the PSSM feature. In our previous work, we combined the PSSM with the physicochemical property feature to predict DNA-binding residues [20] and achieved excellent prediction performance. Therefore, the novel PSSM-PP feature considered six physicochemical properties for each amino acid: the pKa values of the amino group, the pKa values of the carboxyl group [36], the electron-ion interaction potential (EIIP) [37], the number of lone electron pairs, the Wiener index [38] and the molecular mass [39]. Those six physicochemical properties are relevant to DNA-protein interactions and contributed most to improving the prediction performance of DNA-binding residues in proteins compared with other physicochemical properties in the AAindex database [40] when combined with the PSSM feature [20]. Those six physicochemical properties were normalized to the range of 0–1 using the following formula (2):

$$NP_{a}(i)=\frac{P_{a}(i)-\min\{P_{a}(1),P_{a}(2),\cdots ,P_{a}(20)\}}{\max\{P_{a}(1),P_{a}(2),\cdots ,P_{a}(20)\}-\min\{P_{a}(1),P_{a}(2),\cdots ,P_{a}(20)\}}$$ (2)

where *NP*_*a*_(*i*) represents the normalized quantitative property values that range from 0 to 1, i indicates the *i*-th amino acid and *a* is the index of the physicochemical property. Then *NP*_*a*_(*i*) is the value of the physicochemical property a of the *i*-th amino acid.

The PSSM-PP feature was constructed by combining PSSM with six physicochemical properties and took into account the fact that different proteins should have the same vector dimension. The PSSM-PP feature was constructed using the following procedure. 1) Similar to several previous studies [6,14,28,41], all rows in the PSSM were selected that belong to the same amino acid and form a new matrix. Then, 20 new matrices were obtained with the size Ak*20, where *Ak* is the number of amino acids of type *k*. 2) All values in each column were added into each new matrix. Each new matrix was converted to a vector. Therefore, we produced a 20-dimensional vector for each new matrix; a 20×20 = 400 dimension vector was obtained by the PSSM feature. 3) PSSM-PP was generated by merging the 20 amino acid columns of the PSSM into a single column containing the information of a certain physicochemical property. The value in row *a* and column *k* in PSSM-PP matrix, named *S*_*ak*_, was calculated with Eq (3):

$$S_{ak}=\sum_{i=1}^{20}NP_{a}(i)f_{k}(i)$$ (3)

where *a* is the index of a certain physicochemical property, *k* is the index of the type of amino acids in the query protein sequence, *i* is the index of the type of naïve amino acids, *f*_*k*_(*i*) is the scaled value of the *i*-th type of naïve amino acid for the *k*-th type of amino acid in the protein sequence of the PSSM calculated by formula (1), and *NP*_*a*_(*i*) is the normalized physicochemical property values of *a* for the *i*-th type amino acids calculated by formula (2). *S*_*ak*_ represents the index of the type of amino acids *k* in the query protein sequence for a certain physicochemical property *a* and it not only contains the evolutionary information captured by PSSM, but also the conservation information about the amino acid *k* at the level of its physicochemical property *a*. Finally, the dimension size of the PSSM-PP feature was 6×20 (120).

#### Binding propensity measures (BP) and non-binding propensity measures (NBP)

DNA-binding proteins contain DNA-binding residues and the binding residues tend to gather together on the surface of the protein. Therefore, DNA-binding residues could play an important role in identifying DNA-binding proteins. Previously, we constructed a useful classifier named DNABR [20] (http://www.cbi.seu.edu.cn/DNABR/) to predict DNA-binding residues based on sequence information. DNABR outperformed other prediction methods for identifying DNA-binding residues. Therefore, DNABR was used to predict DNA-binding residues to construct binding and non-binding propensity measures in this study. Considering the characters of DNA-binding residues, we constructed two binding propensities measures named BP(1) and BP(2).

The DNA-binding residues, which we used in the binding propensities, were also obtained by DNABR. Therefore the reliability of the prediction needs to be considered. The two binding propensity measures (BP(1),BP(2)) were defined as follows:

$$BP(1)=\frac{\sum_{i=1}^{n}RI(i)}{10N}$$ (4)

where *N* and *n* are the number of amino acids and the number of DNA-binding residues in this protein, respectively; *RI(i*), a positive integer in the range 0 to 10, is the predicting reliability index of DNA-binding residue i generated from DNABR. More reliable predictions will have higher *RI*(*i*) values.

$$BP(2)=\sum_{i=1}^{N-1}\frac{\sum_{k=1}^{n(i)}RI(k)}{10(N-i)}\log_{2}\left(\frac{\sum_{k=1}^{n(i)}RI(k)/10(N-i)}{{\left(\sum_{k=1}^{n}RI(k)/10N\right)}^{2}}\right)$$ (5)

Where *N*, *n*, and *n*(*i*) are the number of amino acids, the number of DNA-binding residues and the number of two DNA-binding residues with the distance i in the query protein, respectively.

*RI*(*k*) is the predicting reliability index of DNA-binding residue *k* generated from DNABR.

For a query protein, *BP*(1) describes the information of the appearance of DNA-binding residues in the amino acid sequence and *BP*(2) describes the correlation of DNA-binding residues in the amino acid sequence and represents the relevance of two DNA-binding residues with different gaps from 1 to *N*-1 amino acids. Furthermore, when $\sum_{k=1}^{n(i)}RI(k)$ equals zero in Eq (5), the problem 0log_2_ 0 appeared in the Eq (5). To solve the problem, Eq (5) was transformed to Eq (6) using a Taylor series.

$$\begin{array}{l} BP(2)=\sum_{i=1}^{N-1}\frac{\sum_{k=1}^{n(i)}RI(k)}{10(N-i)}\log_{2}\left(\frac{\sum_{k=1}^{n(i)}RI(k)/10(N-i)}{{\left(\sum_{k=1}^{n}RI(k)/10N\right)}^{2}}\right)\\ =\sum_{i=1}^{N-1}\frac{1}{\ln\;2}\left[\frac{\sum_{k=1}^{n(i)}RI(k)}{10(N-i)}-{\left(\frac{\sum_{k=1}^{n}RI(k)}{10N}\right)}^{2}+\frac{\left(\sum_{k=1}^{n(i)}RI(k)/10(N-i)-{\left(\sum_{k=1}^{n}RI(k)/10N\right)}^{2}\right)}{2{\left(\sum_{k=1}^{n}RI(k)/10N\right)}^{2}}\right]+o\left(\frac{\sum_{k=1}^{n(i)}RI(k)}{10(N-i)}-\left(\frac{\sum_{k=1}^{n}RI(k)}{10N}\right)\right) \end{array}$$ (6)

#### Physicochemical property feature (PHY)

The physicochemical property feature was constructed based on the formula used in research on prediction of DNA-binding proteins [11], prediction of RNA-binding proteins [42] and functional classification in proteins [43]. Eight physicochemical properties, including hydrophobicity, polarity, polarizability, charge, surface tension, secondary structure, solvent accessibility and normalized Van der Waals volume, were used. Each physicochemical property divided the 20 types of amino acids into three groups. Then, the three descriptors, composition index (C), transition index (T) and distribution index (D), were introduced by the work of Dubchak et al.[44] to represent each physicochemical property. The composition index was calculated by the number of a certain property divided by the length of the query protein. The transition index was obtained by dividing the number of amino acids with a certain property followed by amino acids of a different property by the length of the query protein minus one. The distribution index measures the percent of the length of a query protein within which the first 25%, 50%, 75% and 100% of the amino acid of a particular property are located, respectively. Each physicochemical property generated a feature vector with a dimension of 21, thus the physicochemical property feature has a vector with dimension 168.

### Evaluation method

Cross-validation is a reliable method to test the performance of a new prediction model. We used five-fold cross-validation to evaluate our model. In five-fold cross-validation, the dataset was randomly divided into five parts. The evaluations were conducted five times using four parts as the training dataset to construct a classifier and the remaining part as the test dataset to evaluate the performance. The performance of each model was computed as the average of the five runs.

In this work, four performance measures, namely accuracy (ACC), sensitivity (SE), specificity (SP), and Matthew correlation coefficient (MCC) [45], were calculated to evaluate the prediction performance.

The accuracy is defined as $Accuracy=\frac{TP+TN}{TP+FP+TN+FN}$, which evaluates the overall percentage of DNA-binding proteins and non-binding proteins that were correctly predicted. The sensitivity is defined as $Sensitivity=\frac{TP}{TP+FN}$, which evaluates the percentage of DNA-binding proteins that were correctly predicted as DNA-binding ones.

The specificity is defined as $Specificity=\frac{TN}{TN+FP}$, which evaluates the percentage of non-binding proteins that were correctly predicted as non-binding ones.The MCC is a statistical parameter that assesses the quality of the binary classification and is defined as $MCC=\frac{TP\times TN-FP\times FN}{\sqrt{\left(TP+FP)(TN+FN)(TP+FN)(TN+FP\right)}}$. where *TP*, *TN*, *FP*, and *FN* represent the number of true positive, true negative, false positive and false negative results, respectively. An *MCC* equal to 1 indicates that the model has a perfect prediction performance and *MCC* close to 0 indicates that the model has a random prediction performance.

### Random forest classifier

A random forest (RF) is an ensemble of a large number of classification trees. Each tree in the ensemble is trained on a subset of training instances that are randomly selected from the given training set. At each node, the best split is chosen from a set of variables selected at random from the set of input features. The prediction results of the RF classifier are based on the ensemble of those decision trees and each tree gives a classification result. Finally, the RF classifier selects the prediction result that has the largest number of votes from the classification results. The RF R package [46] was used to implement the RF algorithm.

### Feature selection

The main purpose of feature selection is to remove the least used features from the original feature to improve the prediction performance. In this work, we used the mRMR method combined with IFS to select the prominent features that identify the positive instances from negative ones. The mRMR-IFS method has been used successfully to select important features in several classification studies [47–54].

The mRMR algorithm is a sequential forward selection algorithm first proposed by Peng et al to process microarray data [55]. Each feature selected by the mRMR algorithm has the maximal relevance with target class and the minimal redundancy with other features. A detailed description of the mRMR algorithm can be found in the literature [55], and the mRMR program can be obtained from the website http://penglab.janelia.org/proj/mRMR/.

After the mRMR procedure, the mRMR feature set contained all features. The more prominent features obtained by mRMR algorithm have smaller orders. The IFS step was then used to determine the optimal set of features. Each feature in the mRMR feature set was added one by one from the first to the last. Therefore, N feature subsets were obtained if the mRMR feature set had N features. For each feature subset, an RF was constructed and evaluated by five-fold cross-validation. The IFS scatter plot was drawn with the number of feature subsets as its x-axis and corresponding MCC values as the y-axis. We chose the optimal feature subset when the IFS scatter plot reached a peak.

### The steps of the DNABP method

The following steps were performed and are described as follows:

1. The protein sequence data were collected form the UniProt database.
2. The collected protein sequence data were preprocessed and assigned class labels.
3. The protein sequences were converted to feature vectors.
4. The optimal feature subset was obtained using mRMR-IFS.
5. The RF prediction model was constructed based on the optimal features.
6. The RF prediction model was evaluated.

**Fig 1:**
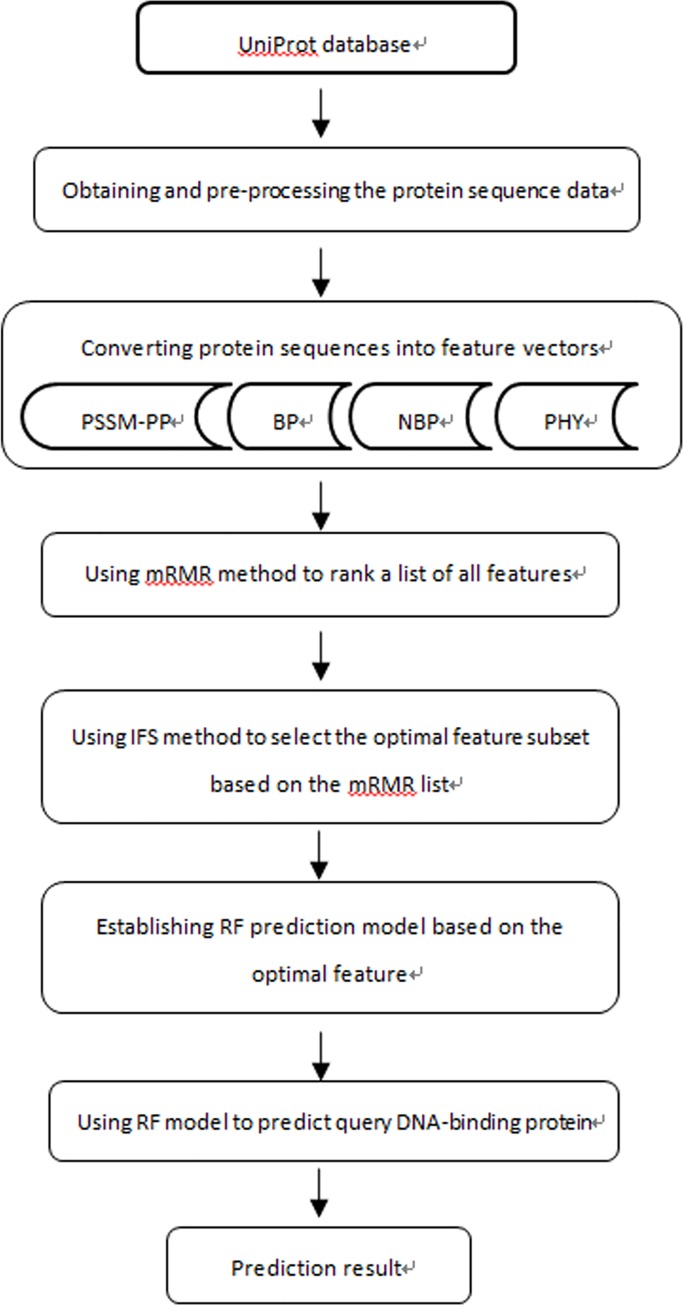
Workflow of DNABP

A detailed flowchart of our work is shown in Fig 1.

## Results and Discussion

### The performance of DNA-binding protein prediction

Based on the Mainset, the different DNA-binding protein prediction models were constructed by RF and various features. The prediction performance of each model was evaluated using five-fold cross-validation (see Table 1).

**Table 1:** Comparison of the performances of various features using the RF algorithm based on Mainset with five-fold cross-validation

Feature	ACC	SE	SP	MCC
**PSSM**	**0.7962**	**0.7602**	**0.8321**	**0.594**
**PSSM-PP**	**0.8169**	**0.7892**	**0.8445**	**0.635**
**PHY**	**0.7765**	**0.7354**	**0.8176**	**0.555**
**PSSM-PP+BP+NBP**	**0.8368**	**0.8101**	**0.8634**	**0.674**
**PSSM-PP+PHY**	**0.8267**	**0.7995**	**0.8539**	**0.654**
**BP+NBP+PHY**	**0.8040**	**0.7688**	**0.8392**	**0.609**
**ALL features**	**0.8464**	**0.8223**	**0.8706**	**0.706**
64 Optimal features*	**0.8690**	**0.8376**	**0.9003**	**0.727**

*The RF-based method with the best parameter (ntree = 1000, mtry = 20)

The classifier using RF with the PHY feature just received a 77.65% accuracy and a 0.555 MCC. When the RF classifier was combined with the PSSM-PP feature only, it obtained a 81.69% accuracy and a 0.635 MCC, which outperformed the prediction performance obtained from the PHY feature. The classifier appending either PHY or BP and NBP features achieved total accuracies of 82.67% and 83.68%. When we constructed classifier using RF with all of the combination of all features, we achieved the best performance, with a 84.64% accuracy and a 0.706 MCC. The results represented that the combination of all features captured more information to discriminate DNA-binding proteins from non-binding ones compared with a single feature. Therefore, we implemented the mRMR-IFS algorithm to select an optimal feature subset from all features, including PSSM-PP, PHY, BP and NBP.

In Table 1, it is worth noting the comparison results between prediction performances obtained by the PSSM-PP feature with that of PSSM. Although the PSSM-PP used a significantly lower size of 120 dimensions in the input vectors than the 400 for PSSM, the PSSM-PP feature improved the prediction performance. This result indicated that PSSM-PP, which provides evolutionary information of the protein at the level of physicochemical properties, could effectively distinguish DNA-binding proteins from non-binding ones. Therefore, PSSM-PP was used as a significant feature rather than PSSM in this work.

### The feature selection results obtained by the mRMR-IFS method

**Fig 2:**
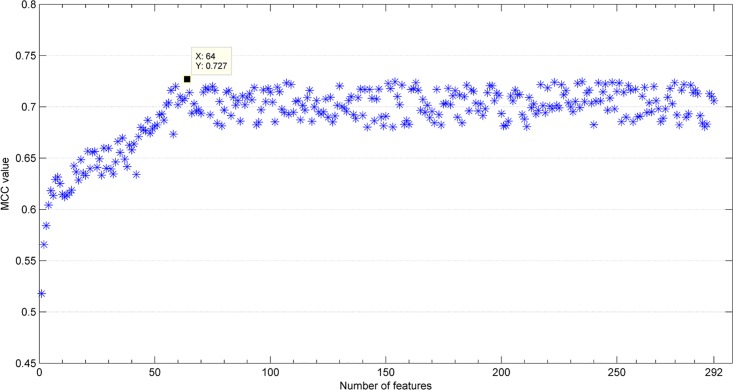
The IFS curve showing MCC values plotted against feature numbers. The maximum MCC value was 0.727 when the top 64 features were selected.

To identify the most prominent features and improve the prediction performance, the mRMR-IFS method was used in this research. First, we used the mRMR method to rank a list of 292 features for the Mainset. A small index value for a feature in this mRMR list represents a more effective power to distinguish DNA-binding proteins from non-binding ones. Second, we used IFS to select the optimal feature subset based on the mRMR list. The 292 different predictors were constructed by increasing the number recursively from rank one to rank 292, and the performance of each predictor was evaluated on the Mainset. The IFS scatter plot was constructed by feature indices and MCC values obtained from the corresponding predictor (Fig 2). A maximum MCC value of 0.727 was obtained using the top 64 features. As seen from Table 1, it is clear that the performance of the prediction model using those 64 features is better than that of the prediction model using all 292 features. The 64 optimal features are shown in Table 2. Finally, the DNABP model for predicting DNA-binding proteins was constructed by the RF algorithm using the 64 optimal features.

**Table 2:** The optimal 64 features for the prediction of DNA-binding proteins

Rank	Feature	p-value
**1**	**PSSM-PP of ARG in the protein sequence for the pKa values of amino group**	**0.00001768**
**2**	**BP(2)**	**0.00002050**
**3**	**PSSM-PP of TYR in the protein sequence for the pKa values of carboxyl group**	**0.00003429**
**4**	**PHY of the solvent accessibility of the composition index for group 2**	**0.00003553**
**5**	**PSSM-PP of GLN in the protein sequence for the electron-ion interaction potential**	**0.00006525**
**6**	**PSSM-PP of ARG in the protein sequence for the molecular mass**	**0.00006760**
**7**	**PSSM-PP of SER in the protein sequence for the pKa values of carboxyl group**	**0.00007535**
**8**	**PSSM-PP of MET in the protein sequence for the pKa values of amino group**	**0.00009378**
**9**	**BP(1)**	**0.00015372**
**10**	**PHY of the hydrophobicity of the composition index for group 2**	**0.00019227**
**11**	**PSSM-PP of ASN in the protein sequence for the pKa values of amino group**	**0.00020420**
**12**	**PHY of the secondary structure of the distribution index of 75% for group 3**	**0.00022754**
**13**	**PHY of the secondary structure of the composition index for group 2**	**0.00023748**
**14**	**PSSM-PP of THR in the protein sequence for the Wiener index**	**0.00026525**
**15**	**PSSM-PP of SER in the protein sequence for the molecular mass**	**0.00026799**
**16**	**PSSM-PP of HIS in the protein sequence for the electron-ion interaction potential**	**0.00028914**
**17**	**PSSM-PP of GLN in the protein sequence for the molecular mass**	**0.00032150**
**18**	**PHY of the solvent accessibility of the distribution index of 50% for group 1**	**0.00032208**
**19**	**PSSM-PP of ARG in the protein sequence for the pKa values of carboxyl group**	**0.00033437**
**20**	**PHY of the hydrophobicity of the distribution index of 75% for group 2**	**0.00035738**
**21**	**PHY of the hydrophobicity of the distribution index of 50% for group 2**	**0.00036935**
**22**	**PHY of the secondary structure of the transition index for group 1**	**0.00038636**
**23**	**PSSM-PP of TRP in the protein sequence for the molecular mass**	**0.00041504**
**24**	**PSSM-PP of HIS in the protein sequence for the molecular mass**	**0.00043729**
**25**	**PSSM-PP of ARG in the protein sequence for the Wiener index**	**0.00049554**
**26**	**PSSM-PP of LYS in the protein sequence for the pKa values of amino group**	**0.00050351**
**27**	**PSSM-PP of PRO in the protein sequence for the pKa values of carboxyl group**	**0.00050751**
**28**	**PHY of the surface tension of the distribution index of 75% for group 2**	**0.00051977**
**29**	**PSSM-PP of THR in the protein sequence for the pKa values of amino group**	**0.00058123**
**30**	**PSSM-PP of THR in the protein sequence for the pKa values of carboxyl group**	**0.00058488**
**31**	**PSSM-PP of HIS in the protein sequence for the pKa values of amino group**	**0.00061484**
**32**	**PHY of the charge of the distribution index of 100% for group 2**	**0.00062851**
**33**	**PHY of the polarizability of the transition index for group 1**	**0.00064670**
**34**	**PSSM-PP of GLU in the protein sequence for the pKa values of carboxyl group**	**0.00065977**
**35**	**PSSM-PP of PHE in the protein sequence for the pKa values of amino group**	**0.00066767**
**36**	**NBP(2)**	**0.00067536**
**37**	**PSSM-PP of ASN in the protein sequence for the pKa values of carboxyl group**	**0.00069458**
**38**	**PHY of the solvent accessibility of the transition index for group 3**	**0.00070656**
**39**	**PHY of the polarity of the transition index for group 2**	**0.00072207**
**40**	**PSSM-PP of TYR in the protein sequence for the pKa values of amino group**	**0.00072656**
**41**	**PHY of the hydrophobicity of the distribution index of 50% for group 3**	**0.00073102**
**42**	**PHY of the hydrophobicity of the distribution index of 75% for group 1**	**0.00074980**
**43**	**PSSM-PP of VAL in the protein sequence for the pKa values of amino group**	**0.00075765**
**44**	**PSSM-PP of GLY in the protein sequence for the pKa values of amino group**	**0.00076497**
**45**	**PSSM-PP of GLY in the protein sequence for the electron-ion interaction potential**	**0.00081140**
**46**	**PHY of the charge of the transition index for group 3**	**0.00081304**
**47**	**PSSM-PP of ILE in the protein sequence for the pKa values of amino group**	**0.00083573**
**48**	**PHY of the hydrophobicity of the transition index for group 1**	**0.00090222**
**49**	**PSSM-PP of ASN in the protein sequence for the molecular mass**	**0.00095262**
**50**	**PSSM-PP of TRP in the protein sequence for the pKa values of amino group**	**0.00096390**
**51**	**PSSM-PP of TYP in the protein sequence for the Wiener index**	**0.00096746**
**52**	**PHY of the polarity of the distribution index of 75% for group 3**	**0.00098588**
**53**	**PSSM-PP of LYS in the protein sequence for the electron-ion interaction potential**	**0.00098960**
**54**	**PSSM-PP of MET in the protein sequence for the pKa values of carboxyl group**	**0.00176958**
**55**	**PHY of the hydrophobicity of the distribution index of 100% for group 1**	**0.00318856**
**56**	**PHY of the charge of the composition index for group 3**	**0.00648432**
**57**	**PSSM-PP of GLN in the protein sequence for the pKa values of amino group**	**0.00961556**
**58**	**PHY of the charge of the distribution index of first for group 2**	**0.01480051**
**59**	**PHY of the surface tension of the transition index for group 3**	**0.02073781**
**60**	**PSSM-PP of ASP in the protein sequence for the molecular mass**	**0.04208745**
**61**	**PSSM-PP of ALA in the protein sequence for the pKa values of carboxyl group**	**0.05963021**
**62**	**PSSM-PP of LYS in the protein sequence for the number of lone electron pairs**	**0.07794997**
**63**	**PSSM-PP of ARG in the protein sequence for the electron-ion interaction potential**	**0.10816428**
**64**	**PHY of the solvent accessibility of the distribution index of 100% for group 1**	**0.15849621**

### Comparison with other research on DNA-binding proteins

There are several studies on the prediction of DNA-binding proteins using sequence information [4–14]. To the best of our knowledge three methods, namely enDNA-Port [11], iDNA-Prot|dis [7] and nDNA-Prot [9], were proposed recently and provide web servers to predict DNA-binding proteins. These three methods all showed better performances when compared with previous methods such as DNA-Prot [28], DNAbinder or iDNA-Port [56]. The predictor enDNA-Prot (http://bioinformatics.hitsz.edu.cn/Ensemble-DNA-Prot/) identifies DNA-binding proteins using physicochemical properties as input features and employing the ensemble learning technique. Liu et al. constructed a predictor, named iDNA-Prot|dis (http://bioinformatics.hitsz.edu.cn/iDNA-Prot_dis/), by incorporating the amino acid distance-pair coupling information and the amino acid reduced alphabet profile into the general pseudo amino acid composition (PseAAC) vector. Song et al. described the predictor nDNA-Prot (http://ndnaprot.aliapp.com/Prediction.jsp), which is an ensemble classifier named for classifying DNA-binding and non-binding proteins using the frequencies of the appearance of every kind of amino acid and physicochemical properties as input features. We used the Testset to evaluate our DNABP in comparison with the other three methods mentioned above. enDNA-Port, iDNA-Prot|dis and nDNA-Prot could predict DNA-binding proteins on the web server; therefore, the Testset was submitted to those three web servers for prediction. As shown in Table 3, the enDNA-Port achieved an MCC of 0.183 with 59.11% ACC, 54.19% SE and 64.04% SP. The iDNA-Prot|dis method achieved an MCC of 0.324 with 66.01% ACC, 73.4% SE and 58.62% SP. The nDNA-Prot predicted all of the proteins as non-binding proteins, therefore the nDNA-Prot achieved an MCC of 0 with 50% ACC, 0% SE and 100% SP. To obtain the performance of our DNABP, the process of constructing the prediction model was repeated based on the Trainset, and then predicted the DNA-binding proteins in the Testset. The ACC, SE and SP of DNABP prediction were 0.7315, 0.6847 and 0.7241, respectively, which resulted in an MCC value of 0.409. The results indicated clearly that our DNABP model achieved the best performance and demonstrates the superiority of our DNABP method, both in feature extraction and selection, compared with the other three methods.

**Table 3:** The performance of DNABP, enDNA-Port, iDNA-Prot|dis and nDNA-Prot based on the Testset

Method	ACC	SE	SP	MCC
**DNABP**	**0.7315**	**0.6847**	**0.7241**	**0.409**
**enDNA-Port**	**0.5911**	**0.5419**	**0.6404**	**0.183**
**iDNA-Prot|dis**	**0.6601**	**0.7340**	**0.5862**	**0.324**
**nDNA-Prot**	**0.5000**	**0.0000**	**1.0000**	**0.000**

In this research, we constructed DNABP model based on the Mainset dataset which is different from the benchmark dataset Xu et al. used to establish enDNA-Prot model [11]. Then the question is that whether a DNABP model constructed based on the benchmark dataset would achieve better performance than the enDNA-Prot model. Therefore, a new DNABP model was trained based on benchmark dataset using 64 optimal features with RF algorithm and test on two independent datasets used in the research of Xu et al. When test on independent dataset1, DNABP model reached accuracy, sensitivity, specificity and Matthew correlation coefficient equal to 89.56%, 89.02%, 90%, and 0.789, respectively. While the enDNA-Prot model achieved accuracy, sensitivity, specificity and Matthew correlation coefficient equal to 84.62%, 73.18%, 94% and 0.7, respectively [11]. When test on independent dataset2, the prediction performance of our DNABP model is also outperforms that of enDNA-Prot model (See Table 4). Those results show that our DNABP method superior to the enDNA-Prot method.

**Table 4:** Comparison of the performances of DNABP and enDNA-Prot based on various test dataset

Model	Test dataset	ACC	SE	SP	MCC
**DNABP**	**Independent dataset1**	**0.8956**	**0.8902**	**0.9000**	**0.789**
enDNA-Prot*	**Independent dataset1**	**0.8462**	**0.7318**	**0.9400**	**0.70**
**DNABP**	**Independent dataset2**	**0.8599**	**0.8571**	**0.8626**	**0.720**
enDNA-Prot*	**Independent dataset2**	**0.8171**	**0.8455**	**0.7905**	**0.64**

*The results are obtained from reference [11]

### The feature selection results

**Fig 3:**
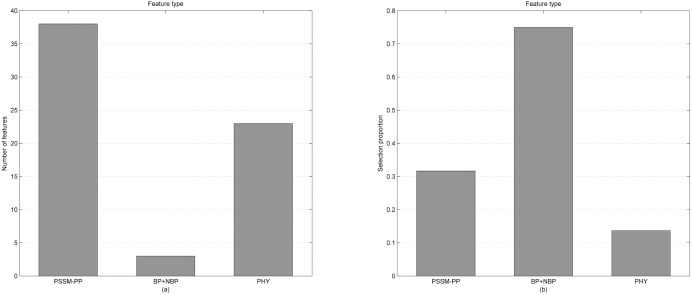
(a) Feature distribution for the 64 optimal features. (b) The selection proportion of each type of feature.

Based on the mRMR-IFS method, we selected 64 features as the optimal feature subset from 292 original features. The 64 features outperformed all 292 features for distinguishing DNA-binding proteins from non-binding ones. The 292 features are divided into three types: PSSM-PP, BP/NBP and PHY and the number of each type of feature in the optimal feature subset is shown in In recent years, rapid advances in genomic and proteomic techniques have generated numerous DNA-binding protein sequences. In 2014, the number of DNA-binding proteins in the UniProt database was more than 10 times greater than that in 2000. These large amounts of data provide the foundation for research on the identification of DNA-binding proteins using computational approaches. Fig 3A. There are 38 PSSM-PP features, three BP/NBP features and 23 PHY features in the optimal features subset. Therefore, in the optimal subset, the number of PSSM-PP features is the highest and the number of BP/NBP features is the lowest. Considering that the number of each type of feature is different, we calculated the proportion of each type of selection feature for the corresponding type of feature. As shown in Fig 3B, we found that although the number of BP/NBP features in the optimal feature set was the lowest (3), the selection proportion of BP/NBP features was the highest (75%). This result indicated that BP/NBP features play an important role in the prediction of DNA-binding proteins. The number of PSSM-PP features is lower than the number of PHY features in the original feature set, while the number of PSSM-PP features is higher than the number of PHY features in the optimal feature subset. Thus, PSSM-PP features have the second largest selection proportion and PHY features have the smallest selection proportion. This result indicated that PSSM-PP features are more effective than PHY features in distinguishing DNA-binding proteins from non-binding ones. Taken together, these results proved that the results obtained in Table 1 are reliable. We also investigated the statistical significance of the differences for these features between DNA-binding proteins and non-binding proteins on the Mainset. The p-values of a two-sample t-test were calculated and are shown in Table 2. A small p-value indicated greater separation and large p-values indicated less separation. As seen from Table 2, 53 out of 64 (53/64 = 0.828) features have a p-value less than 0.001. This result, that 64 optimal features selected by the mRMR-IFS method have statistically significant differences between DNA-binding proteins and non-binding proteins, indicated that those features are useful for separating the DNA-binding proteins from non-binding proteins and could greatly improve the prediction performance for DNA-binding proteins.

### Analysis of 64 features obtained by the mRMR-IFS method

#### Analysis of PSSM-PP features in 64 optimal features

**Fig 4:**
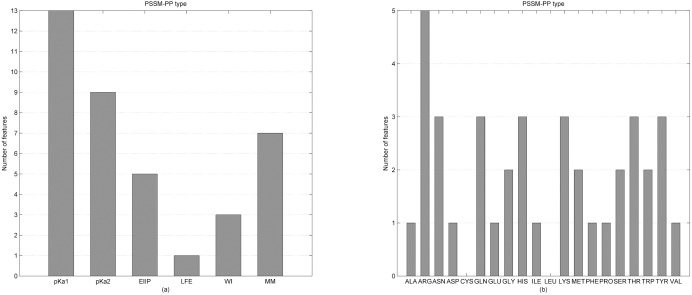
(a) Physicochemical property distribution of the 38 PSSM-PP features that were selected in the optimal feature set. (b) The type of amino acid distribution used to construct the 38 PSSM-PP features that were selected in the optimal feature set.

Thirty-eight PSSM-PP features were selected in the optimal features subset. Among the 38 selected PSSM-PP features, there are 13 features constructed by the pKa values of amino groups, nine features constructed by the pKa values of carboxyl groups, five features constructed by the electron-ion interaction potential (EIIP), one feature constructed by the number of lone electron pairs, three features constructed by the Wiener index and seven features constructed by the molecular mass. The contributions of each type of physicochemical property that constitutes the PSSM-PP features are shown in Fig 4. This result showed that among the six physicochemical properties, pKa values of the amino group and pKa values of the carboxyl group were selected the most and the number of lone electron pairs was selected the least. This shows that the pKa values of the amino group and pKa values of the carboxyl group play important roles in DNA-binding protein prediction and that the number of lone electron pairs contributes least to the prediction of DNA-binding proteins, which is consistent with the result obtained in Table 2.

#### Analysis of BP and NBP features in the optimal features

The mRMR-IFS method selected two BP features and one NBP feature among the 64 optimal features, which means that only one NBP feature was not selected in the optimal feature subset. The high selection proportion suggested that BP and NBP features contribute most to distinguish DNA-binding proteins from non-binding ones. As shown in Table 2, the p-values of BP and NBP features between the binding proteins and the non-binding ones were much less than 0.001. This result also indicated that BP and NBP play a vital role in discriminating between DNA-binding proteins and non-binding proteins.

The BP/NBP features selected in the optimal feature subset were BP(1), BP(2) and NBP(2). The BP(1) feature represented the information of the appearance of DNA-binding residues in the query protein. The selection of the BP(1) feature reveals the reliability of the definition of the BP(1) feature that DNA-binding residues should appear in the DNA-binding proteins. BP(2) and NBP(2) represent the correlation of DNA-binding residues with DNA-binding residues and non-binding residues with non-binding residues in the amino acid sequence, respectively. The selection of BP(2) and NBP(2) indicated that the BP(2) and NBP(2) formulas, which represented the spatial information in DNA-binding proteins and non-binding proteins, respectively, were reliable. NBP(1) was not selected as an optimal feature, possibly because the number of non-binding residues is greater than the number of DNA-binding residues in the majority proteins, which would result in no statistically significant difference between DNA-binding proteins and non-binding proteins.

#### Analysis of PHY features in the optimal features

**Fig 5:**
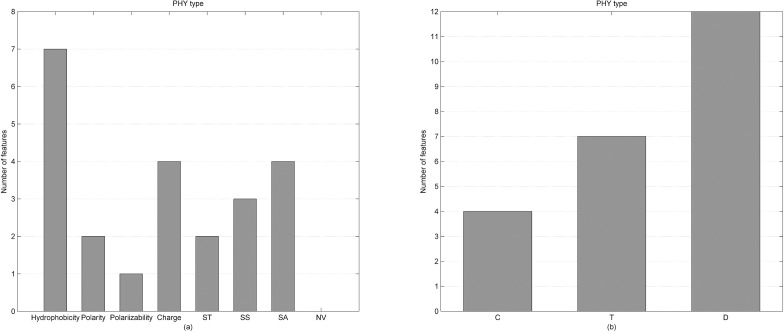
(a) Physicochemical property distribution used to construct the 23 PHY features that were selected in the optimal feature set. (b) Distribution of the three descriptors used to construct the 23 PHY features that were selected in the optimal feature set.

Twenty-three PHY features are in the optimal feature subset, and their distribution is shown in Fig 5. The 23 PHY features were divided into eight types by physicochemical properties, including hydrophobicity, polarity, polarizability, charge, surface tension, secondary structure, solvent accessibility and normalized Van der Waals volume. As seen from Fig 5A, there are seven PHY features obtained from hydrophobicity property, which was the most among the eight physicochemical properties. The charge property and the solvent accessibility property both have four PHY features, which were the second most among the eight physicochemical properties. These results indicated that the three physicochemical properties were more useful for revealing the mechanisms of DNA and protein interactions than the other five physicochemical properties. A possible explanation could be: 1) DNA-binding residues in binding proteins should cluster on the surface of the proteins to bind to DNA; therefore, binding residues would tend to be hydrophobic residues, and the solvent accessibility property of DNA-binding residues should be stronger than that of non-binding residues; 2) DNA-binding residues tend to be positively charged so that they can easily interact with DNA, which is negatively charged. The polarizability property only has one PHY feature and the normalized Van der Waals volume did not have any PHY feature in the optimal feature subset. Thus the polarizability and the normalized Van der Waals volume contributed least to distinguishing DNA-binding proteins from non-binding ones.

The 23 PHY features were divided into three groups by the descriptors, which are composition index (C), transition index (T) and distribution index (D). As shown in Fig 5B, the C index has four PHY features, the T index has seven PHY features and the D index has 12 features among the 23 PHY features in the optimal feature subset. Each physicochemical property generated 21 PHY features and the C index generates three PHY features, the T index generates three PHY features and the D index generates 15 PHY features. Although the D index has the most features in the optimal feature subset, the selection proportion of the D index is the least (10% (12/(15*8)). The selection proportion of the T index is the most among the three descriptors (29.2% (7/(3*8)), which suggested that the T index contributed most to predicting DNA-binding proteins.

### The reliablility of negative samples in the Mainset

As mentioned in “Dataset” section, the mainset was comprised by 7131 non-binding proteins randomly selected from the negative dataset and all of the the 7131 DNA-binding proteins in the positive dataset. The question arises then, whether the random selection of different dataset of 7131 non-binding proteins would change the prediction performance. Therefore other four randomly selected datasets of non-binding proteins was used to construct the DNABP model. Four dataset of 7131 non-binding proteins randomly selected from the negative dataset were respectively combined with 7131 DNA-binding proteins in the positive dataset and form four main dataset named Mainset_1, Mainset_2, Mainset_3 and Mainset_4. The predicton performances of DNABP models which built respectively from four main datasets using the RF algorithm with all of the 292 features were list in Table 5. The performance of four DNABP models which built from four different main datasets were very similar to the performance which obtained from Mainset. The result shows that the 7131 negative samples in Mainset is reliability to constructed DNABP model.

**Table 5:** Comparison of the performances of various dataset using the RF algorithm based on 292 features with five-fold cross-validation

Dataset	ACC	SE	SP	MCC
**Mainset**	**0.8464**	**0.8223**	**0.8706**	**0.706**
**Mainset_1**	**0.8443**	**0.8260**	**0.8626**	**0.689**
**Mainset_2**	**0.8527**	**0.8547**	**0.8507**	**0.705**
**Mainset_3**	**0.8436**	**0.8446**	**0.8425**	**0.687**
**Mainset_4**	**0.8612**	**0.8622**	**0.8602**	**0.722**

## Web server

Based on the 64 optimal features selected by the mRMR-IFS method, a web server DNABP was developed to identify DNA-binding proteins from amino acid sequences. DNABP is freely available at http://www.cbi.seu.edu.cn/DNABP/. On the DNABP web page, users can submit an amino acid sequence in FASTA format. The DNABP model was established using the RF algorithm on the Mainset. The RF algorithm is implemented using the R package [46]. After submitting the query sequence, the DNABP web server returns a quick prediction result that is sent to the user by e-mail. The DNABP server also returns the binding information of each residue, which is predicted by DNABR when the query protein is predicted as the DNA-binding protein.

## Conclusions

To predict the DNA-binding proteins using sequence information, we proposed a new and useful method, DNABR, which combines an RF algorithm and an mRMR-IFS feature selection method. The method has novel features, including evolutionary information that combines conservation information with the physicochemical properties of amino acids (PSSM-PP), binding propensity measures (BP) and non-binding propensity measures (NBP). The results proved that these features markedly improved the predictions. The mRMR-IFS feature selection method was implemented to obtain the optimal feature subset. The RF model with the novel optimal feature subset selected from the hybrid feature set, including PSSM-PP, PHY, BP and NBP, achieved excellent performance with 86.90% accuracy, 83.76% sensitivity, 90.03% specificity and an MCC of 0.727. A comparison between DNABP and other prediction methods indicated that our DNABP method is currently the most effective method to predict DNA-binding proteins using only sequence information. A web server named DNABP (http://www.cbi.seu.edu.cn/DNABP/) has been developed to aid the use of the DNABP model to predict DNA-binding proteins.

## Supporting Information

S1 Table The accession numbers of UniProt entries of 14262 proteins in main dataset. (DOCX)
