# Supplementary material for: DNABP: Identification of DNA-Binding Proteins Based on Feature Selection Using a Random Forest and Predicting Binding Residues
Source: PLoS One. 2016 Dec 1;11(12):e0167345. doi: 10.1371/journal.pone.0167345 (PMC5132331; doi:10.1371/journal.pone.0167345)
Supplement: S1 Table — (DOCX) [file pone.0167345.s001.docx]

Table S1. The accession numbers of UniProt entries of 14262 proteins in main dataset

| **Main dataset (14262 proteins)** | | | | | | | | | | | | |
| --- | --- | --- | --- | --- | --- | --- | --- | --- | --- | --- | --- | --- |
| 7131 DNA-binding proteins | | | | | | | | | | | | |
| Q6A8L0 | | A6NI15 | | Q8N1G0 | | Q83NU2 | P48383 | P29673 | | Q0V7X4 | | Q9M9R4 |
| Q7V7T9 | | Q60EQ4 | | Q83950 | | O34709 | P20134 | Q7XM16 | | Q8ITI5 | | Q8GXM7 |
| Q9ZUP2 | | P0C983 | | Q8BXJ2 | | P0A9U6 | Q9XFH4 | Q9FV71 | | Q52QH4 | | P27369 |
| Q2JVG1 | | A4WGY5 | | Q5EXX3 | | Q9XFB0 | Q06481 | Q9SCK6 | | Q9L9G1 | | Q9LQZ2 |
| Q9K4Q3 | | P35638 | | Q69GZ5 | | A0R692 | Q9ZUL5 | Q54P62 | | Q6ZDF3 | | P39792 |
| Q4FV41 | | P0DJA5 | | P0CQ67 | | Q9S758 | Q8H506 | Q84JG2 | | P44694 | | P75433 |
| P9WNR8 | | Q6VAM4 | | P0CO92 | | Q9NQ33 | Q58371 | A5DF43 | | Q9MA32 | | Q55854 |
| Q5Z3Z8 | | A8FKI7 | | Q99MU3 | | P43965 | O59741 | P36631 | | Q46855 | | P57690 |
| A0LUZ1 | | P67711 | | Q9YUS3 | | P56179 | Q5AQ33 | P77730 | | Q9LSI7 | | P36704 |
| Q4FUU7 | | P0ACI8 | | Q8CHC8 | | P68350 | Q24151 | Q10127 | | O22176 | | P30198 |
| Q96IR2 | | Q923G2 | | Q17308 | | P0CY07 | Q9NL40 | O23487 | | P30979 | | P04892 |
| A1AU70 | | Q3SZN5 | | Q92618 | | Q9FLI1 | O31522 | O02326 | | P52696 | | P40914 |
| Q8ZJH2 | | P58768 | | Q9Y2K7 | | Q9KC68 | Q9YW29 | G5ECG2 | | P61798 | | P34555 |
| Q8U9U6 | | P55323 | | Q9UNY4 | | P0CK38 | Q9ULD5 | P38531 | | Q9LSL6 | | Q1KKU7 |
| Q4JYF5 | | Q9D084 | | Q7Y1C4 | | Q9DB38 | P47988 | P09309 | | P81392 | | P60319 |
| A2Y5G8 | | B0R2R5 | | Q8WW38 | | P0AC51 | O61660 | Q23696 | | P76086 | | P21308 |
| Q9MBF7 | | Q5F410 | | P30958 | | Q9QYL0 | Q57809 | P11414 | | Q7F9W2 | | O34381 |
| P62722 | | P0ACH0 | | P35251 | | Q64364 | O31626 | Q54RY6 | | O06987 | | O31560 |
| Q8YC76 | | Q8ZSD2 | | P20585 | | Q923Z4 | P39529 | P13528 | | A6NJG6 | | Q9FLI0 |
| A9WQ89 | | Q9PLI1 | | Q4G0F8 | | O24646 | Q9SY74 | Q9LTS4 | | Q9M128 | | G4RJY9 |
| Q7RXR3 | | P0C9Y7 | | O76332 | | Q8BHG9 | A6QNQ6 | Q9UUI6 | | Q53W21 | | A6NLW8 |
| Q9RVT3 | | P0C9E5 | | Q9QSK1 | | P75542 | O18531 | P59583 | | P16944 | | Q9M1U2 |
| B9MN06 | | P06116 | | P00519 | | P12305 | Q9C5P0 | Q681I0 | | P37459 | | Q5XEN5 |
| C5BKM1 | | P62536 | | P47806 | | Q5FUK4 | P14736 | Q54513 | | P37078 | | P70354 |
| B1MXB4 | | A8MT69 | | Q6BRF0 | | Q9FGC9 | Q9P0L1 | P14196 | | Q5RM09 | | Q9X1E1 |
| P42147 | | P0C9Y0 | | Q779J8 | | O83063 | Q14872 | Q9C6V4 | | P44876 | | B1H0B1 |
| Q89WI8 | | O28646 | | P82094 | | P42179 | Q9SLC6 | Q04688 | | P35547 | | Q26605 |
| Q9HUW0 | | P17138 | | A7NPT5 | | Q9C995 | A6UN73 | Q7T6Y0 | | P40759 | | Q9VM17 |
| Q7VKV0 | | P51587 | | Q75AA7 | | P56958 | P91943 | Q96BR6 | | P37930 | | Q38895 |
| A2RHP2 | | Q8QG78 | | B2RRE4 | | P52937 | Q7M3K2 | Q9W6G7 | | F4HY61 | | Q0K2R3 |
| Q47VK9 | | P46100 | | P97305 | | Q28772 | Q9Y3M9 | Q7TP98 | | Q9Z3Q6 | | Q03435 |
| P40287 | | Q80UV9 | | P57528 | | P10842 | Q6NQ79 | P13468 | | P54722 | | P47236 |
| Q9KSW4 | | Q7S133 | | Q8BX22 | | Q0MXD1 | Q9TZL0 | Q9XTJ4 | | Q04013 | | Q9SX41 |
| Q9CAF6 | | P03355 | | Q9Y4E5 | | Q82CI4 | Q9UFB7 | O18141 | | P39140 | | P20384 |
| Q4QLV2 | | Q92547 | | Q5EBL2 | | Q44333 | P16376 | Q5ZDJ6 | | Q9LW31 | | Q97TX9 |
| Q00056 | | P81128 | | F4I6M1 | | Q48660 | Q9LTJ8 | P96663 | | P17224 | | C5CXR6 |
| P37218 | | Q80TE0 | | P0DJH0 | | P37960 | Q5AXT5 | Q19802 | | Q8RV83 | | Q86QH9 |
| A7ZMC4 | | Q5UZ40 | | Q8NFZ0 | | Q8LC30 | Q8N895 | Q06003 | | O34920 | | P40267 |
| Q47YA8 | | P42704 | | Q6Z4T3 | | P0DJA6 | O14270 | P52605 | | P48987 | | P17488 |
| Q2QW53 | | E1B328 | | P59923 | | P59697 | P52551 | Q9TZ99 | | Q10B98 | | Q2QW55 |
| Q46577 | | Q9NSC2 | | Q12704 | | A6QJJ8 | P38699 | P38022 | | Q8H115 | | Q9M8K6 |
| A5F6Z2 | | P09546 | | Q6PGE4 | | Q8LG10 | Q5ANJ4 | Q06183 | | P07270 | | B2KCD9 |
| Q4QK86 | | O43345 | | O22243 | | P9WJA6 | Q8LJT8 | P15005 | | P72131 | | Q9SJ09 |
| P9WMF4 | | Q4KWZ7 | | P03008 | | P62574 | Q54GG8 | Q23294 | | P05683 | | P22645 |
| Q9L4K2 | | P27570 | | Q9JUK9 | | P66857 | P49956 | Q04073 | | L0TCG5 | | Q7Z142 |
| P36720 | | C4ZJU1 | | Q64028 | | P0A0Z0 | P28468 | P44152 | | P03691 | | P9WPY8 |
| A7MXB2 | | P9WGF3 | | Q9BVI0 | | P80944 | Q8MLV1 | Q9Y330 | | Q5VV52 | | P10083 |
| Q4QN02 | | P73340 | | Q9H2U1 | | Q1IKG0 | Q86IH1 | Q13952 | | Q9FGB0 | | Q71U11 |
| O54479 | | C7GP35 | | Q9Y467 | | O07127 | Q8T2A4 | Q8LT07 | | Q9CAA4 | | Q9LTF7 |
| Q9KTY3 | | A2YX04 | | Q8IX07 | | O80542 | Q9SW80 | P03006 | | P37517 | | Q84J78 |
| P52653 | | A6QQ70 | | O70157 | | P01551 | O13658 | P76639 | | Q9FZA4 | | Q196Z2 |
| Q8EA81 | | B7ZRU9 | | Q9ZJ57 | | Q980W9 | Q9WY22 | Q10MN8 | | P46068 | | Q10186 |
| A7MFW6 | | Q9ESZ8 | | Q01954 | | P0ACS6 | Q8L6Z7 | Q93ZL5 | | O34859 | | P40923 |
| P36601 | | P38935 | | Q93575 | | D4GZB4 | P41895 | Q00858 | | P03030 | | P10038 |
| Q9X600 | | Q54UV8 | | P25941 | | P9WMH8 | Q869R9 | P70434 | | O45521 | | B8DSV1 |
| Q6FCX7 | | Q24087 | | B2RQL2 | | O32237 | P28698 | O42954 | | Q9C9N3 | | Q8W0F1 |
| Q9R9M8 | | A4IFA3 | | Q8NDX5 | | P0DKR7 | Q9P8W3 | A9A1T2 | | Q93246 | | P37640 |
| P22161 | | P06400 | | O76906 | | P9WMJ4 | Q24312 | Q9M126 | | Q9FRV4 | | O69280 |
| B0TDI0 | | Q8HWS3 | | P47540 | | O27001 | Q8IRH5 | Q09565 | | O02235 | | P26797 |
| Q82EA6 | | Q9Y6K1 | | A4PBL4 | | Q28GF5 | Q24558 | P41933 | | Q9M2Y9 | | O10440 |
| P18096 | | Q92GB7 | | D2EAC2 | | P22874 | Q01842 | Q9ZXB2 | | P36798 | | Q9HSF6 |
| B1JS04 | | Q04RT2 | | P22670 | | P9WMI4 | P14164 | E1BEA8 | | Q9SY12 | | Q9GNV2 |
| Q7N976 | | Q9UQ84 | | Q6N043 | | O00488 | Q5A4H5 | Q3V4R9 | | P77700 | | Q5VS55 |
| P17396 | | Q8N5U6 | | O44952 | | P93822 | P18494 | P55373 | | Q9USU7 | | Q9RAJ1 |
| B2GH83 | | Q9HR31 | | Q99MT2 | | Q6MT21 | A3BH85 | Q02361 | | Q9LRH6 | | P37928 |
| Q6DA22 | | Q80V63 | | Q5T7W0 | | P9WJB6 | Q01295 | P53050 | | P52689 | | Q56185 |
| Q03256 | | P03073 | | Q72TG0 | | Q9NRG0 | Q20595 | O14229 | | P45349 | | O81313 |
| O87455 | | Q10EL1 | | Q0PA12 | | Q9ZD50 | Q6C7B9 | Q01856 | | Q6NV18 | | Q47718 |
| Q1I490 | | Q60611 | | Q8FPC1 | | P9WN84 | Q01582 | P17899 | | P07273 | | P27872 |
| C6DF27 | | Q08050 | | Q03188 | | P0AFP2 | Q8ZS46 | O10368 | | Q9C5T3 | | P54884 |
| Q8TIN0 | | A0R3R7 | | Q8WY36 | | C1CYP4 | O43952 | Q22127 | | O28487 | | P96676 |
| A8APW0 | | Q59046 | | P37938 | | P0C961 | A8WL06 | P12768 | | P76369 | | P39334 |
| Q9L6X9 | | Q9X105 | | Q01831 | | Q9S426 | P03690 | P71389 | | Q9FRL5 | | P06227 |
| A8AJA5 | | P52550 | | Q8IXF0 | | Q5JGD9 | Q5ISE2 | Q8GXT3 | | O76762 | | P28815 |
| P29178 | | O69460 | | C7J0A2 | | Q9NR55 | Q5RIX9 | Q9FLU1 | | P39413 | | Q9P0T4 |
| P04198 | | Q9R1D1 | | Q9RAA9 | | P06933 | Q04996 | B8FFL8 | | Q9FXA3 | | P42587 |
| A1JI47 | | Q5JMM1 | | Q9WU40 | | B3H469 | Q24143 | Q04849 | | Q9C9F5 | | Q9LMK2 |
| C6DK30 | | Q6FSK8 | | Q9NYF8 | | P0CY11 | P38766 | Q84WJ2 | | O82632 | | Q8S151 |
| Q8R9V5 | | P53528 | | P58501 | | O34533 | P22808 | O02219 | | P52684 | | O69055 |
| Q890K1 | | Q80X44 | | O86810 | | P0ACN2 | Q7QDU4 | O42115 | | Q9N2K7 | | P06019 |
| O13535 | | Q5VK71 | | P0CS63 | | Q66EU5 | P45612 | Q9SFZ3 | | O35038 | | O96642 |
| A1YEP8 | | O82802 | | P54784 | | Q9ZU26 | Q9P2Y4 | Q21701 | | Q7XHI7 | | P55411 |
| P27235 | | Q96T25 | | P30320 | | P46003 | Q01593 | P12959 | | Q97Y84 | | Q9RMY4 |
| P0A824 | | P0A5A6 | | Q6KAQ7 | | P0CY13 | Q59P39 | Q8NS92 | | Q8S9H7 | | P42097 |
| Q9CG78 | | Q87039 | | Q5SVQ8 | | P10841 | Q9H9D4 | P38944 | | Q8LDR0 | | Q9VGJ5 |
| A7FN79 | | O88907 | | Q15361 | | Q8F052 | G5EGD2 | Q26366 | | P61827 | | O02761 |
| A7ZV62 | | P46063 | | Q14934 | | O21945 | P46605 | Q9SX27 | | Q91424 | | Q707Y7 |
| P36808 | | G3MY25 | | Q7Z5Q5 | | P9WF36 | Q94818 | Q75JL2 | | B2JMF6 | | Q58CM8 |
| B7UJV9 | | A7BJS7 | | P18751 | | Q9PFB1 | Q5JNA1 | P38128 | | P28809 | | C5CFS3 |
| P16058 | | Q10086 | | Q9ZJE9 | | P9WMI6 | P51023 | Q9NU63 | | P52679 | | P96701 |
| P15172 | | P52323 | | Q9Y2X9 | | Q01239 | Q9PU53 | Q8TFF3 | | P32512 | | B0I1G7 |
| Q8G7B5 | | Q39204 | | Q8BI67 | | P55922 | A7TPF8 | P41154 | | O94424 | | P96440 |
| A7ZUI3 | | Q9UN79 | | A8XLS0 | | C0SPB6 | Q24248 | Q9C9T2 | | P03009 | | P94548 |
| A4W0M7 | | Q04864 | | Q60953 | | P0A9T7 | Q25BI3 | A2CEZ5 | | P77171 | | O55208 |
| P27223 | | Q2TA17 | | Q9L4M7 | | P0AE71 | Q9W4S7 | P34028 | | O31249 | | Q9P7S9 |
| P54662 | | Q9LUZ4 | | Q0VG06 | | Q8FDB5 | O88942 | A0LE53 | | P41817 | | Q8REJ7 |
| Q5M623 | | Q5NUA6 | | Q7M3M6 | | P22753 | Q54GH0 | Q8J1X7 | | Q9FYK5 | | P35165 |
| Q9YAX2 | | A9Q1D5 | | Q9UBT6 | | P51237 | P41073 | F4JYZ8 | | Q9FJF4 | | P52286 |
| Q8CX05 | | Q5F4B2 | | Q9UEG4 | | Q00572 | Q9RT63 | P38141 | | P52686 | | Q93WU9 |
| B1HPM0 | | Q2TBI2 | | O02799 | | O53333 | F4I443 | P11107 | | A3BKM2 | | P45215 |
| Q8EQ23 | | O95365 | | O88509 | | P09075 | Q5AK51 | Q9SLB9 | | C4ZGY6 | | Q76EJ0 |
| C1G966 | | Q9UGP5 | | A6ZM04 | | Q9UTN9 | Q9SUM4 | Q9NRM2 | | Q851V1 | | Q7WY76 |
| P74391 | | P03680 | | O25722 | | Q16559 | Q3V0C1 | Q9NWS9 | | O66401 | | O07001 |
| Q9EZJ8 | | Q9P0K8 | | Q60636 | | P17530 | O65420 | Q5A5Q6 | | Q96I27 | | Q9SSW0 |
| P25035 | | Q6BY37 | | P03361 | | A0M2C1 | Q59QC7 | P09083 | | Q9I6Z9 | | Q6DN03 |
| O02786 | | Q9BWE0 | | P42226 | | Q01256 | O15060 | Q04719 | | P35692 | | Q8SRR1 |
| P24314 | | Q9Z1T5 | | Q9LE38 | | E0TZF9 | Q0J952 | P80074 | | Q7XRX3 | | Q9UW19 |
| Q65U90 | | Q2TA45 | | Q9BYV9 | | P03131 | Q5Z5Q3 | Q6NQK2 | | P34663 | | Q2GDJ1 |
| P45599 | | Q70KY4 | | Q94F88 | | P03052 | O15816 | Q24535 | | P37728 | | P45785 |
| A8AHC2 | | P98152 | | P39678 | | Q822W7 | Q8AYI2 | Q8LFV3 | | C1F735 | | O34549 |
| Q1RHT8 | | Q51761 | | A2QJX5 | | P11873 | P35901 | P39572 | | Q9FDW1 | | P40396 |
| A6H907 | | A7TMJ6 | | P74397 | | Q03576 | P11420 | Q9MAT6 | | O49459 | | Q1ECI2 |
| P40764 | | A2BGM5 | | Q9Z103 | | P68676 | Q59MJ1 | Q53PH2 | | Q04803 | | Q8SR09 |
| P10103 | | Q851W4 | | Q7Z4V0 | | P03042 | P02788 | B1APH4 | | Q73NE4 | | Q9ADP7 |
| Q2K619 | | Q5NAN5 | | Q00839 | | P08874 | P49699 | Q0P9V4 | | P55389 | | P46581 |
| Q80905 | | Q00613 | | P28284 | | Q8FHF3 | P07112 | G7WMP6 | | O31443 | | Q7G1L2 |
| P20264 | | P56282 | | O77638 | | P03130 | P08153 | A1L2F3 | | Q03615 | | P08707 |
| Q65LS8 | | Q0VBL1 | | Q9UKN8 | | P67699 | Q5ZAY0 | P09084 | | C9X1G6 | | Q9ZR15 |
| Q9I8V2 | | Q8N884 | | Q4V348 | | O33347 | B5X582 | P19541 | | P34618 | | A6NFQ7 |
| P04682 | | P07928 | | Q9SWB4 | | Q6MX01 | P36417 | P0CAP4 | | P17678 | | Q9SN52 |
| A8ACM1 | | B5VGZ3 | | Q47281 | | P9WF24 | Q86TG7 | P41225 | | P26798 | | Q12041 |
| A8ADG3 | | A1YEQ3 | | Q6BSE2 | | B7UTE9 | Q9P7H9 | Q54QY7 | | P39399 | | O34857 |
| P11826 | | P20839 | | Q12172 | | Q23946 | Q06335 | Q06184 | | Q9SVB7 | | P51704 |
| A7ZIA5 | | Q6F6A2 | | Q6L4V0 | | Q52277 | Q0DVU4 | Q5ANB1 | | Q9SUS1 | | P52933 |
| Q9CD72 | | O97716 | | O93746 | | P9WF08 | A5DU46 | Q9R002 | | Q9FKM5 | | P22159 |
| P06921 | | B8DN19 | | Q13105 | | P9WF42 | P54789 | Q21878 | | P30864 | | Q6GKL1 |
| A7Z017 | | P03110 | | Q8W4L5 | | P0AE72 | O00910 | Q6IR52 | | Q2FPW6 | | P32398 |
| P28356 | | Q00566 | | P75352 | | P9WJ87 | P34184 | P10025 | | O49515 | | O34892 |
| Q0J3Y6 | | A5E4Y7 | | Q5RD14 | | O60356 | Q12039 | P32031 | | Q9KAV6 | | Q9LIB5 |
| Q52BY4 | | P68335 | | Q8N393 | | Q9VQS6 | Q8J257 | Q24318 | | Q9ZN79 | | Q9JN69 |
| O29910 | | Q14774 | | Q3URR7 | | P9WJ44 | Q90625 | Q60688 | | Q945M9 | | O68583 |
| P81409 | | Q60641 | | P16383 | | P0C079 | Q75DE9 | P06991 | | Q05587 | | Q9FGJ3 |
| Q8U526 | | Q6Z358 | | Q9UIU6 | | P80001 | Q9FT73 | Q08792 | | Q8SVS9 | | A9BIJ5 |
| Q7YRJ7 | | O06581 | | Q6XP49 | | P9WKT7 | P35995 | Q9LJG8 | | Q9FL62 | | B5YKF0 |
| P10577 | | Q9LVG2 | | C4XS37 | | P9WJ84 | P32325 | Q5IW40 | | P77402 | | Q9XIB4 |
| P18102 | | A5PK30 | | Q04411 | | Q52279 | P49866 | A9CB86 | | P39376 | | Q0DXB1 |
| Q1KKX5 | | Q8K923 | | Q6ZSB9 | | P21344 | P33748 | P39959 | | P77744 | | P94433 |
| Q8HZP6 | | Q9FAX2 | | P95979 | | P15342 | Q75K81 | Q9SJN0 | | Q04778 | | O32228 |
| P10571 | | Q91XE9 | | B2V693 | | P81552 | Q338N2 | P03835 | | P46363 | | Q94ID6 |
| O42131 | | Q9PU65 | | Q9ZLL4 | | Q8ILR9 | P39875 | P07065 | | P25547 | | Q6CPW2 |
| Q1CMQ0 | | Q5FVM4 | | O43422 | | Q96JG9 | P13989 | Q0WNR2 | | Q67TP9 | | O87883 |
| P51128 | | Q5Z818 | | Q9UI36 | | Q9M3G7 | Q06149 | Q64759 | | Q98SH9 | | Q8EWT6 |
| P12902 | | P06990 | | P29503 | | F4IHS2 | Q5NB85 | O61764 | | P20027 | | Q9YAD5 |
| O08307 | | Q4R8G6 | | E9PZZ1 | | Q8I1N6 | P17214 | O74205 | | Q9NAH2 | | Q1G3B8 |
| Q5UYM9 | | Q13283 | | Q6IV72 | | Q6ZRS2 | Q657B3 | Q9HCK0 | | P14815 | | P45904 |
| A0R979 | | Q46259 | | Q8NCD3 | | Q9NDJ2 | Q9URT4 | P23874 | | P52659 | | O34643 |
| A2TED3 | | O13161 | | Q6P3Y5 | | Q96L91 | P55965 | Q86AD6 | | P26950 | | Q64756 |
| P31271 | | O95947 | | Q9HC78 | | Q8CHI8 | Q89AK2 | Q8GWJ4 | | P43460 | | Q7Z5D8 |
| O06978 | | Q8NAM6 | | Q9UNA4 | | P28167 | P05709 | P30052 | | P52667 | | P59927 |
| Q9HPF2 | | O95232 | | P97302 | | E9QAM5 | Q9HDX1 | Q5JLB5 | | Q47129 | | Q9SJK7 |
| Q9ZMV7 | | P28324 | | Q8N196 | | Q8IN94 | Q9NX65 | Q12950 | | Q1KKR7 | | Q8TY11 |
| P30333 | | Q9W734 | | P0C7P8 | | Q9W0T1 | Q9P326 | Q23175 | | P39592 | | B2RD01 |
| P55317 | | Q14653 | | Q6E7H0 | | Q9BYK8 | Q9HGM8 | Q9BJK5 | | J7FCF0 | | Q2N2K6 |
| Q62414 | | P70338 | | P85037 | | Q9C0A1 | P74893 | Q6MHJ5 | | Q62814 | | Q9CIV9 |
| P36279 | | P03660 | | P17888 | | Q22258 | Q12753 | Q64749 | | P20668 | | P04527 |
| Q87BY1 | | P0A0C6 | | P13010 | | Q9VXG8 | P96574 | Q39199 | | P53768 | | Q6NLD5 |
| Q9CMY2 | | Q5DU99 | | P37398 | | Q924A2 | P55368 | Q6ZSS3 | | Q1KKZ2 | | P34326 |
| Q8X6I1 | | Q5ZHN5 | | P57220 | | Q96JM2 | O96006 | O83097 | | O32504 | | P40573 |
| P10152 | | P29558 | | Q00790 | | P14248 | Q84JE8 | Q27355 | | P32338 | | P32265 |
| A7ERG1 | | Q9Y4X4 | | Q4VC44 | | Q5U249 | P54269 | Q93XW7 | | Q9FHH1 | | Q45585 |
| Q7Y0V7 | | Q52873 | | P75437 | | Q9NEL2 | Q96TL7 | Q60420 | | P77746 | | P76053 |
| Q4U3Q4 | | Q9FDX8 | | Q9Y253 | | Q54RD4 | P29741 | O17611 | | O34574 | | P75488 |
| Q98867 | | F1Q514 | | Q61AP6 | | A1Z8P9 | Q25132 | P46372 | | Q9FKK2 | | Q9KVG9 |
| P50512 | | P52439 | | Q9Y2K1 | | Q9DGT6 | O74252 | P24219 | | Q50900 | | P44923 |
| Q4L8V4 | | P0ACI4 | | P17531 | | Q18ER3 | P54529 | Q5ANI6 | | P39647 | | P35907 |
| Q83II5 | | Q8JI10 | | P23246 | | Q11107 | P22813 | P47980 | | P32805 | | Q9LYB9 |
| P0ACE3 | | Q8GXB3 | | P20311 | | Q9C0G0 | O60393 | Q9FWS3 | | Q8T0D4 | | Q9UW22 |
| Q4L4Y3 | | O43502 | | A6ZTU2 | | Q68DE3 | F5HCV3 | Q26263 | | P37641 | | Q4PSR7 |
| Q740J9 | | Q5EAD3 | | Q99941 | | F4KBP5 | O26901 | Q95QD7 | | Q9FMB6 | | Q5XVH0 |
| Q9GLW0 | | P11901 | | P25980 | | Q6EVK6 | Q10067 | O02151 | | Q8W1E3 | | Q8U1L3 |
| Q1RI04 | | Q05749 | | O34204 | | Q9SGH2 | Q9HB58 | P38826 | | Q6H7J5 | | P04524 |
| Q1KKX7 | | P78426 | | Q4WPQ8 | | P10180 | P52087 | Q12415 | | Q8RWU3 | | Q8IUE0 |
| Q61473 | | Q08DR0 | | P42166 | | Q9UIF8 | P13574 | P18745 | | P94678 | | Q38Q40 |
| P17535 | | P53566 | | Q9Y4A8 | | P34706 | P53038 | Q12398 | | O04609 | | Q9LQ28 |
| P31277 | | P18848 | | Q9C5P1 | | Q9DE13 | Q9HBE1 | P60470 | | P45008 | | P37929 |
| P19782 | | Q9XH36 | | P78424 | | P51123 | O05069 | Q8T053 | | P52044 | | P31357 |
| B6EGC9 | | Q7RTV3 | | Q940D0 | | C0H4W3 | Q9FG77 | O41107 | | Q9LSQ3 | | P42584 |
| P49731 | | P14316 | | Q8BY02 | | Q7X9V2 | Q9UXG1 | P52957 | | Q9ZW81 | | Q8NHV9 |
| Q00578 | | Q99M85 | | P0C7T5 | | B5BT18 | P87235 | Q10655 | | Q98PK2 | | Q02486 |
| Q8HZ00 | | P23511 | | Q9NQV8 | | Q6UDF2 | P15038 | Q8EIX3 | | Q2V9B0 | | Q9C9I2 |
| P80205 | | P03098 | | Q75IP6 | | Q8LK56 | Q557I1 | P07664 | | Q746H2 | | O23875 |
| P0CE59 | | P0A5R3 | | O51528 | | F4J7T3 | Q53H47 | A1YKT1 | | P49330 | | Q54X41 |
| P51505 | | Q16342 | | Q29CW2 | | O43065 | Q17768 | O60158 | | P31449 | | Q9M2U4 |
| P27256 | | Q6DCW1 | | Q16650 | | Q13129 | P08979 | P10069 | | P77559 | | A8M8Z5 |
| B0R7F7 | | Q8L999 | | Q9ZMY3 | | Q4PGL2 | P32432 | Q753Y2 | | Q9FKQ6 | | Q05286 |
| B2IGF1 | | Q91998 | | Q96BD5 | | F4J7T2 | O14978 | Q60296 | | Q8KB21 | | P55559 |
| P30667 | | P0ACN8 | | O15062 | | Q9SZ67 | Q58884 | Q8S897 | | Q43385 | | Q9FGH2 |
| Q14814 | | P14908 | | P29128 | | Q766Z3 | O01409 | P34474 | | P54161 | | Q9SNC0 |
| P31310 | | Q5E9X1 | | Q8R3L2 | | Q9VXE6 | P40333 | Q5AFK0 | | Q47141 | | Q08784 |
| B0T360 | | Q62233 | | Q16IB4 | | Q6R7C9 | Q09838 | Q99335 | | P52670 | | O74045 |
| Q86567 | | Q5RAJ7 | | Q97AI2 | | P32333 | Q9NPA5 | P23737 | | P40883 | | Q8H1D2 |
| Q2FDM8 | | Q04713 | | Q05516 | | Q7Z401 | Q9SJ56 | P40193 | | Q9SKZ1 | | Q8L925 |
| Q8PQ45 | | Q99853 | | Q6BLT3 | | Q9HFR4 | Q2HR71 | Q5Z8L1 | | P16942 | | Q94AW5 |
| Q2NX28 | | Q19A41 | | Q14190 | | O14981 | Q6YVX9 | Q9BXG8 | | P35884 | | Q707Y6 |
| Q06924 | | Q9SNW8 | | Q3U288 | | Q9UL36 | P53968 | Q6X5Y6 | | Q57748 | | Q3IYV6 |
| Q4PFV5 | | Q2RY21 | | Q0DA50 | | Q68CP9 | P22121 | Q04787 | | Q96N20 | | Q98PV9 |
| A6R7S1 | | Q6PJQ5 | | Q2TB10 | | Q9U1H0 | Q9LIH5 | Q01622 | | Q9C7S0 | | O35009 |
| P10266 | | Q92DX9 | | Q8NI51 | | Q4P328 | Q54Y85 | Q0WV96 | | Q8LC79 | | P71015 |
| P30666 | | P0A9F8 | | Q99607 | | O33845 | Q9VS05 | Q09415 | | Q54L82 | | P17487 |
| O73673 | | P31316 | | O97472 | | O15015 | Q12224 | P39933 | | O14210 | | P07156 |
| Q8V2N7 | | O68014 | | Q9JL61 | | Q5JET0 | Q6FRL4 | Q9ZUU0 | | P18921 | | P39667 |
| P22605 | | P50222 | | Q96FV9 | | Q22516 | Q9LTS7 | Q9NSD4 | | Q9SKD9 | | P79373 |
| Q76HI7 | | Q6AUG0 | | Q6YZ49 | | P34333 | Q07243 | Q8GWK2 | | P40917 | | Q9YFV8 |
| Q95JA5 | | Q5ZI72 | | P13099 | | Q9SIV5 | Q6CR69 | Q22555 | | O31456 | | P36627 |
| B5RHS5 | | Q9RY51 | | O22781 | | P11283 | Q9YVX9 | Q9P7D9 | | O74986 | | Q8RVQ9 |
| Q2L4T2 | | P05827 | | Q54T16 | | O92815 | P57781 | P11455 | | P16931 | | B2CW77 |
| Q8TK90 | | F4I7L1 | | O15353 | | Q5UQR0 | Q9YB01 | O77215 | | Q9M161 | | Q84NC2 |
| Q8U4R1 | | Q46M54 | | Q6R0H1 | | Q8TEK3 | Q5N6V0 | P09085 | | P52691 | | P75811 |
| Q5JFD3 | | Q8GRL4 | | Q9NTW7 | | F4IV99 | P32389 | P25555 | | Q50EX6 | | Q8VUP4 |
| P50541 | | Q9D902 | | P45512 | | Q557C9 | Q9NA51 | Q9S7L2 | | P12254 | | P94370 |
| Q8MIH8 | | Q7XH85 | | P44680 | | Q54B29 | P03134 | Q77MR5 | | P23841 | | P37554 |
| Q88XZ6 | | Q9JJZ6 | | Q9FN69 | | Q9NK54 | Q8H2D5 | P55449 | | Q06596 | | P77626 |
| P0ACG0 | | Q5M948 | | Q00184 | | Q6F598 | Q06339 | Q8S3Q9 | | Q0MXD0 | | A1RY32 |
| Q6C0B5 | | O42173 | | Q9Z2E2 | | P29617 | C4Y1P0 | P52741 | | Q6R0G4 | | Q11094 |
| P28340 | | Q6PZD9 | | O74915 | | B7ZS37 | Q9UDV7 | Q9LZJ8 | | Q8JJ26 | | P51894 |
| Q5QMZ9 | | P29692 | | Q925Q8 | | P52746 | P40059 | Q9HEV5 | | Q9LT89 | | P07249 |
| P92948 | | Q6P5C5 | | B8AX23 | | P77933 | P71229 | Q3EAI1 | | P49373 | | O94588 |
| Q8XD86 | | P26993 | | P23799 | | Q9VDY1 | B0X0K1 | Q6H7U2 | | P33634 | | Q03081 |
| P51179 | | P59800 | | Q9NQX1 | | Q58295 | Q9C2N1 | Q7KM13 | | O34827 | | P32607 |
| Q9BQW3 | | Q9C056 | | Q9UBL3 | | A0Q5Y3 | Q9ZUM0 | Q9N4Q7 | | P77309 | | O29501 |
| Q9PTN2 | | O86820 | | O24606 | | O58530 | Q6BQR8 | P23758 | | P96725 | | Q6ZT77 |
| P53409 | | Q01IK0 | | Q9M548 | | A7U6F1 | P01105 | Q9NX45 | | Q9FJ16 | | Q00423 |
| Q8RED1 | | P43697 | | Q5ND04 | | Q58907 | Q55GK3 | Q22236 | | Q9FNY3 | | Q707Y3 |
| P25144 | | Q32KY3 | | Q7F1M0 | | Q5BAZ5 | P40656 | Q57594 | | Q9FE22 | | O34843 |
| O93385 | | Q9R1S4 | | Q8BUR3 | | Q96RK0 | P28614 | B3MB79 | | P42507 | | O05409 |
| A6TEB9 | | Q9LX82 | | Q9ZJA1 | | P35820 | P98149 | Q96LX8 | | A4Z012 | | O83101 |
| O46238 | | Q9HBU1 | | P75426 | | Q95YM8 | Q54F11 | O23090 | | Q9FH59 | | O07458 |
| Q82I57 | | Q93WV7 | | O25149 | | P39769 | Q5XVF0 | P03870 | | O15266 | | Q7Z2G1 |
| Q8CNQ7 | | Q8X4Z9 | | Q24762 | | A5DCV3 | Q873Y3 | Q9UBD0 | | P54000 | | Q9X1T8 |
| P58378 | | P0ACM7 | | Q96MX3 | | Q9U6A1 | Q5SJ47 | Q54IF9 | | Q9SHB5 | | Q9LHY9 |
| P0CO24 | | Q4JAI6 | | Q96CK0 | | Q12766 | Q08400 | Q19691 | | Q9SB92 | | Q796S4 |
| Q9YEY2 | | P63541 | | Q8N554 | | P74918 | Q5JNZ3 | Q93WU7 | | P94423 | | Q06909 |
| P37474 | | Q8FFE0 | | Q9LYP5 | | P38859 | Q08AG5 | P71073 | | O32255 | | P06023 |
| B4L1Z2 | | Q84XB3 | | Q69NK8 | | Q9W539 | Q96JL9 | O45460 | | P37499 | | Q874N1 |
| P17010 | | Q13886 | | P13027 | | Q9HDV4 | Q9W352 | Q9CQM6 | | P39127 | | P07040 |
| P29496 | | P70660 | | Q91431 | | P28859 | P55879 | P80073 | | P06229 | | Q9SJT8 |
| A0JMF8 | | P75989 | | Q9P215 | | Q54SV8 | O43058 | Q6YTQ4 | | Q6NU14 | | Q6DSS2 |
| Q9C1A4 | | Q8X835 | | O64235 | | Q8RXS6 | P79005 | P34536 | | O93958 | | Q1PES7 |
| Q9U903 | | P63202 | | P22544 | | P57071 | P07636 | O14979 | | P16400 | | Q01414 |
| O94130 | | P51858 | | P13360 | | Q24167 | O28469 | O45291 | | Q52154 | | P23484 |
| Q6IEI0 | | A2Y5N0 | | P32807 | | Q54BK0 | Q8MSG8 | Q5T0B9 | | P33122 | | F4KH89 |
| Q5IS44 | | Q57SD4 | | Q15326 | | P14284 | Q2QMT7 | P54366 | | P96660 | | A8ABJ9 |
| P16989 | | P97436 | | Q9P2K5 | | Q03468 | Q94890 | Q9ZSL6 | | P40950 | | A2BLW2 |
| Q80GM6 | | Q3C1V8 | | Q9HGI2 | | Q9VGI8 | Q8GT06 | Q9S840 | | P55181 | | Q8VWQ5 |
| Q9QUS4 | | P0ACM2 | | P14199 | | Q9P6L6 | Q45032 | Q55E93 | | Q1PFD1 | | O07627 |
| Q3MCB5 | | I7CA98 | | P59055 | | F4HW51 | Q5B8L0 | Q9ZVH0 | | P94403 | | Q6XD76 |
| Q5L299 | | P33779 | | P62598 | | Q5SXM2 | Q9PHK2 | P82976 | | P52666 | | Q5KVB2 |
| Q0VCE2 | | E7S4M0 | | D3YU81 | | P13382 | Q58434 | P10035 | | Q1INE2 | | Q9SR34 |
| P18739 | | A0R666 | | P0CS57 | | P63135 | Q5W9E7 | Q54TY7 | | O18214 | | Q872F4 |
| A2SQM6 | | P69202 | | Q05950 | | Q9Y767 | A5WFR0 | P35192 | | P96662 | | F4I8U3 |
| P18957 | | P66808 | | O73932 | | A2BGR3 | P22807 | P25293 | | O34701 | | Q58038 |
| A7X3L7 | | P0ACU3 | | P97459 | | Q6FK48 | A8WW61 | Q6EWX1 | | Q7X9H9 | | O22259 |
| P54479 | | Q9KJC4 | | Q15723 | | Q2UTQ9 | P04197 | P09077 | | G3ECR2 | | P31538 |
| A2YQ56 | | P82979 | | Q8BII1 | | Q27395 | Q6CXS0 | Q53N87 | | O67550 | | Q09926 |
| Q8CP71 | | Q02582 | | P10258 | | P39113 | O83258 | Q8NC26 | | P18289 | | A0RYF4 |
| B3MMA5 | | Q3MHZ1 | | Q9LNJ5 | | Q1ZXH0 | P41412 | P28515 | | Q6C6V8 | | P55613 |
| Q6DV79 | | A0R4Z6 | | Q03654 | | Q02362 | P43079 | Q9TXJ1 | | O59753 | | P70790 |
| P51514 | | Q2YDP3 | | Q96IT1 | | F4I8S3 | O54003 | Q9XVV3 | | Q8GWF1 | | P25150 |
| Q6YVY0 | | Q4R7I4 | | Q9UGN5 | | Q2RL77 | Q9UTA7 | Q27350 | | O31500 | | O43812 |
| O43524 | | P0ACZ6 | | Q9P2F9 | | P28040 | Q6ZK57 | P22711 | | O07906 | | P34496 |
| Q6GN21 | | Q683D7 | | Q52QU2 | | Q9T0I1 | A4ZU91 | Q09602 | | Q50899 | | Q58006 |
| Q08346 | | P77170 | | Q8IZM8 | | Q54MH9 | Q6C030 | Q7SXV2 | | Q8LE43 | | Q5EBM4 |
| Q12948 | | Q9SW63 | | Q2RY11 | | P19538 | B5X570 | Q1PFE1 | | O13074 | | Q9H609 |
| Q7PYU6 | | P0ACT8 | | O95243 | | Q9URU2 | Q89703 | P71635 | | Q26602 | | Q98924 |
| Q96SC8 | | Q8U3H5 | | Q54TN2 | | A4IHD2 | O50224 | Q98JM5 | | Q08143 | | P39784 |
| Q8GGL1 | | Q00P32 | | Q9EQJ4 | | Q9SJQ6 | P40814 | P79007 | | Q00840 | | P21678 |
| Q00958 | | Q6NH62 | | Q8L7L8 | | Q4PGG5 | F1R983 | P06184 | | Q84LH3 | | O05070 |
| Q4UNG8 | | P04130 | | Q4Z9G3 | | Q9S775 | Q01473 | Q01080 | | P50902 | | P55658 |
| Q2KI09 | | C7U331 | | Q5YGP8 | | P20482 | Q58352 | A5HBE1 | | P43011 | | P40762 |
| Q2NKI2 | | P37195 | | Q9H5J0 | | A9KTE6 | Q94CJ8 | P38621 | | P44540 | | O94704 |
| A8GQ15 | | P63205 | | Q9GZV8 | | Q9US25 | Q9XIN0 | Q56YJ8 | | F4KIX1 | | A8MZ59 |
| Q9W7R5 | | P0AFW1 | | Q569E7 | | B2SU53 | P19880 | Q8IEF6 | | Q8GW17 | | Q7XGM6 |
| P17430 | | Q823K0 | | Q1CW50 | | Q9FL92 | Q9SU25 | O18048 | | Q5UQ00 | | P44580 |
| P39788 | | Q5LII9 | | P51333 | | Q67MD5 | P32367 | P27476 | | P20428 | | Q9LQW3 |
| Q2FVN3 | | P53568 | | Q8R0T2 | | Q19272 | P43634 | O73718 | | F4K5X6 | | P04546 |
| P0CF50 | | P0A583 | | Q84K00 | | P25172 | P06225 | P18119 | | P96343 | | Q0JP11 |
| Q872I5 | | Q3KJK7 | | Q75LL6 | | A9X4T1 | Q9C469 | Q59029 | | Q9C983 | | P42585 |
| Q9YCB6 | | A7HH57 | | Q96T92 | | Q6BGY8 | Q15397 | P14707 | | Q7EZD5 | | Q7RTU0 |
| P89453 | | O33060 | | Q80VM4 | | Q54I65 | O31644 | P34454 | | P40331 | | O31541 |
| P28932 | | P55360 | | Q4R5U8 | | Q2KHR2 | O95125 | Q8R0S1 | | Q8W2F2 | | O33817 |
| G4MZJ4 | | Q80920 | | Q8BG36 | | Q91690 | Q6K977 | Q9SVU6 | | Q93W88 | | P42195 |
| Q01K26 | | P06931 | | P0CO51 | | Q9U7E0 | Q92076 | Q56XU4 | | C6W9U8 | | P31078 |
| P10275 | | P37582 | | Q9ZN49 | | P11978 | P13054 | Q9UXF8 | | P11746 | | O05404 |
| Q8QGQ6 | | Q64221 | | Q8R4E9 | | P51521 | Q02100 | O61734 | | P0CAP3 | | Q9PPT7 |
| P38132 | | Q7ABA6 | | P49868 | | F4JAA5 | P40489 | Q04635 | | Q8LAV5 | | P12551 |
| Q8WYA1 | | P87558 | | Q60976 | | Q05854 | P18723 | P57620 | | P24042 | | P43461 |
| Q9YE64 | | P37376 | | O48652 | | Q9UST7 | P08763 | Q9FKK6 | | Q9KPI4 | | O05236 |
| O95409 | | A3N1H2 | | P0DH85 | | B2GEY4 | Q24256 | Q10938 | | Q53W62 | | Q9FMC2 |
| A6R7F0 | | P0CG11 | | Q96KC8 | | P13002 | Q6CTH5 | Q26486 | | Q84JK2 | | P74739 |
| Q9D6K9 | | Q056Q2 | | O76808 | | Q8BP86 | Q6DG03 | Q8GYY1 | | P19071 | | O65155 |
| Q9X3Y3 | | P40622 | | O95201 | | Q9SR66 | Q58559 | O62389 | | Q99020 | | I6Y8F7 |
| Q9I9A3 | | P24094 | | A2WW87 | | O23277 | P12364 | B7GSG2 | | P79011 | | Q0BXT3 |
| P61802 | | P0A9E4 | | Q9Z0E3 | | Q9NVI1 | Q24266 | Q2S6G1 | | P27426 | | Q90963 |
| Q1DFP6 | | Q64256 | | P43064 | | Q09811 | Q20646 | Q0JGI1 | | P77396 | | Q9M1U4 |
| Q11NR3 | | Q02347 | | Q64321 | | Q54FL0 | P12258 | Q59KL6 | | Q856K7 | | O13706 |
| Q8PRG0 | | P33875 | | Q9H175 | | Q9H582 | Q16587 | Q6EU30 | | P77379 | | Q6DRA6 |
| P18184 | | Q63245 | | Q9GNL2 | | O04716 | Q9USR9 | A1L4X7 | | Q26604 | | Q9FZ51 |
| P0CF63 | | A9LZQ2 | | P52288 | | P07248 | P17789 | O22763 | | Q66LM5 | | P79404 |
| A0R8S0 | | B0B9S0 | | Q86YH2 | | P09547 | O15945 | P45815 | | P19769 | | P16146 |
| P14971 | | P0ACH1 | | Q57623 | | Q51334 | O60129 | Q09728 | | Q9M0K4 | | P17311 |
| O57337 | | Q8PD37 | | Q15554 | | Q54FX2 | Q8TD17 | P50901 | | P52685 | | Q8GW46 |
| P58253 | | P40274 | | P0CN24 | | Q9GQN5 | Q9PPZ6 | Q15270 | | Q9C598 | | P96708 |
| Q8FLP9 | | P03670 | | P74839 | | O17582 | Q19720 | Q28571 | | P32677 | | Q80VX4 |
| Q8R2Y9 | | Q57120 | | Q9FXG8 | | Q1INB1 | P50831 | Q8N7U7 | | Q7X7N3 | | Q4PSK1 |
| Q76MX4 | | P05835 | | Q93WT0 | | O64146 | P45158 | Q9YFU8 | | Q8N8Q3 | | Q707Y8 |
| Q81QX8 | | Q2EES9 | | Q8BI73 | | Q8ZGC7 | P40522 | Q9MA98 | | Q1PDN3 | | Q9LUJ3 |
| Q8WNV1 | | P26634 | | P18748 | | Q9HCE3 | F1NPG5 | Q10PR4 | | P82970 | | O07582 |
| P40634 | | P37549 | | Q9UL17 | | F4K3G5 | Q54VB4 | P29129 | | Q1KKY1 | | O33682 |
| P24928 | | P31537 | | Q5ABZ2 | | Q94F87 | C9JSJ3 | P06435 | | Q853W0 | | P51783 |
| P0CT41 | | P31505 | | O65685 | | Q6GZW7 | Q54PU1 | O73790 | | Q93WV4 | | O69450 |
| P03261 | | Q62388 | | Q14781 | | Q24595 | Q10445 | P24607 | | P46117 | | Q43472 |
| B3DJM5 | | Q06A37 | | P84445 | | Q9SD34 | Q5H9K5 | Q9Y7L4 | | P37767 | | O23310 |
| O97676 | | Q8TD26 | | Q9GZX5 | | O13682 | Q4PGT8 | P56672 | | Q5UQA4 | | O04681 |
| Q805F9 | | Q9DE14 | | Q7Z3I7 | | Q6FLP2 | Q04505 | Q05543 | | Q9LV17 | | Q9D7K2 |
| P25847 | | Q09XV5 | | P42681 | | P35207 | P33520 | G5EE86 | | P03069 | | Q15929 |
| Q9TUZ1 | | P31629 | | P51448 | | O74842 | Q9FGT7 | Q3V4S9 | | Q9LR74 | | O82248 |
| P03070 | | O14497 | | Q94C33 | | P90520 | P74143 | Q58184 | | Q8NML3 | | Q9LMK5 |
| Q298W7 | | Q63505 | | Q16YA8 | | B2A610 | P10105 | P24813 | | Q9YGN6 | | P23992 |
| Q8TE85 | | Q9Z985 | | Q8R2T8 | | Q9BL02 | Q9S9P3 | Q06453 | | Q75GR5 | | P17637 |
| Q5R6A9 | | P30317 | | Q45119 | | Q9QSK2 | P40631 | Q9NU39 | | P51721 | | Q7JUR5 |
| Q8C208 | | Q3UH06 | | Q561R1 | | Q57532 | Q68ED3 | P24647 | | P43458 | | O31564 |
| P45448 | | Q86U86 | | P70056 | | Q10135 | Q9LL45 | Q8NBB4 | | Q6ZMS7 | | O58782 |
| A1CU75 | | Q640Z6 | | Q84J39 | | D0EL35 | P31503 | Q9M9B9 | | P17410 | | Q5V5E2 |
| Q5XGT8 | | P03354 | | Q66JL1 | | Q554C5 | Q04049 | Q17103 | | D2KQB0 | | P46910 |
| O60548 | | Q5RES4 | | Q9UL58 | | P52172 | P26047 | P23250 | | Q9LXV2 | | E1V7V9 |
| Q1A1A6 | | Q5T890 | | Q54ZP8 | | Q5UQE6 | Q54WN7 | Q8H507 | | P17211 | | Q88FY0 |
| P23769 | | D3ZGB1 | | Q9XI90 | | Q55GU9 | O83045 | P34670 | | Q26600 | | Q97QZ2 |
| A0PJY2 | | Q9BX26 | | O43474 | | Q8LKW0 | Q23976 | Q15697 | | Q9WYG1 | | P76062 |
| O16963 | | Q9QJ16 | | Q8NIZ4 | | P06494 | Q21006 | Q8N693 | | P32788 | | P18515 |
| P36198 | | Q6AHZ1 | | Q8BUZ3 | | P15801 | Q54RM2 | Q9LUH8 | | Q5VR07 | | O05217 |
| P09065 | | O93530 | | Q62158 | | O74502 | Q66652 | P43130 | | Q0DBW8 | | P29556 |
| O04134 | | Q86US8 | | Q9H8N7 | | P20193 | Q6BKP5 | P18016 | | Q966L8 | | O07920 |
| Q0JPT4 | | P54132 | | Q8W4H1 | | Q9NG98 | P33749 | O94714 | | Q9TTE5 | | Q91975 |
| Q9Y2V3 | | Q9H4Z2 | | Q8N2W9 | | Q8XYE3 | Q6FLG1 | Q6C9I6 | | Q9PWL7 | | Q9SIZ0 |
| P25545 | | Q9HAW4 | | P51815 | | Q03834 | Q04437 | Q06126 | | O33813 | | Q05092 |
| Q32PF6 | | Q6P4R8 | | Q9QXK2 | | Q9NUA8 | P71366 | Q2R9D2 | | Q6K9U2 | | Q58948 |
| Q9YGT0 | | Q925H1 | | B0R7U1 | | O59610 | O14628 | Q26656 | | Q92373 | | P42547 |
| A1YEY5 | | Q2NKX8 | | O75603 | | Q54XG7 | Q94KL5 | P20154 | | Q9ZQ19 | | O34692 |
| P18816 | | B1MZM2 | | Q80932 | | P34127 | P38830 | O17934 | | Q58520 | | Q9FN29 |
| P79891 | | P9WMQ4 | | Q6K687 | | Q9JKB5 | Q07635 | Q94JW8 | | Q53QI0 | | O07465 |
| Q2YV56 | | Q2NKJ3 | | Q5TC79 | | Q7S1P9 | O51066 | P32511 | | P26833 | | P56901 |
| Q8DET1 | | P14629 | | Q9LS40 | | A1Z9R4 | P33400 | Q9LVB0 | | Q7RTU5 | | A3DN07 |
| B5XCB8 | | O43593 | | P78022 | | P55265 | Q9UTN0 | P21957 | | P39075 | | P27555 |
| Q5PMJ1 | | Q89AB3 | | Q9FJL4 | | E1X022 | Q5A4F3 | P15593 | | Q93356 | | Q3E922 |
| Q864P9 | | Q81ZL2 | | O57685 | | P25357 | P51022 | P10485 | | Q00753 | | O77618 |
| A1TEV0 | | Q97ZF5 | | P33293 | | Q54Z40 | P34707 | Q9M1G6 | | P03627 | | Q7N1I3 |
| Q2FJ20 | | Q8IUX7 | | P9WJW3 | | Q9YC75 | P38889 | O77027 | | Q8LFJ8 | | Q9FZP6 |
| Q6NTS2 | | Q6N1B7 | | P18629 | | P55201 | P36011 | Q8LSZ4 | | Q8VCI7 | | Q2RVT5 |
| P56659 | | Q9Y2G1 | | O43541 | | Q7YU81 | P22336 | Q93WJ9 | | Q6XL52 | | O32181 |
| P30665 | | P11204 | | Q9BUG6 | | Q9X3X7 | Q5IZC5 | P03857 | | P39781 | | P0C1H6 |
| Q8R4S5 | | Q08999 | | P29549 | | P45157 | B4NBL5 | A0ZZA1 | | Q9SJ07 | | P09532 |
| Q9UKY1 | | O75717 | | Q8NAP8 | | Q80Y50 | Q8NBF1 | Q59VR1 | | Q58161 | | B0R717 |
| P41739 | | Q12986 | | Q9LSE2 | | A7U6F2 | Q9FIE3 | P49453 | | Q9FUD3 | | Q8R5U7 |
| O15119 | | P87164 | | P47974 | | A0JMF1 | O36398 | P35693 | | Q9SVX5 | | O31472 |
| Q293F6 | | P9WIQ4 | | Q5R8K4 | | B6DMK2 | Q2QPW2 | Q657C0 | | P77569 | | O31592 |
| Q9QB84 | | P9WMQ2 | | A2RV70 | | P22374 | Q8H181 | O22759 | | P43465 | | O34949 |
| O70230 | | C9X1G5 | | Q28ZA9 | | O52236 | P41829 | Q67NG0 | | Q9FGD1 | | P49925 |
| Q9NQB0 | | Q17R98 | | P97443 | | P24349 | Q9P6I9 | A4LBC0 | | Q93WV6 | | P30734 |
| Q6FML7 | | P0CJ78 | | O25916 | | P17671 | Q9I9A7 | Q8S2E6 | | Q7ZZ00 | | Q10370 |
| Q861Q9 | | Q6NPP4 | | O28484 | | Q55750 | Q6BSZ4 | P54992 | | Q9ZN78 | | P22392 |
| Q9NZI7 | | Q9Z7G7 | | P10226 | | Q24U48 | P54790 | Q96LI6 | | Q9LTA2 | | P71049 |
| C7GJZ2 | | P03599 | | Q9CAA9 | | Q01529 | Q9N4B8 | P52604 | | Q8GVZ6 | | Q9CYR0 |
| P62509 | | P19212 | | Q796Q6 | | O13527 | P27526 | Q9PHE9 | | Q94398 | | Q641M3 |
| F1QDF8 | | Q14527 | | O35892 | | Q58524 | B0FIL5 | P17222 | | A2Z1U1 | | P06455 |
| Q15562 | | Q6CQX2 | | Q24753 | | Q8IMA8 | O74954 | Q9C518 | | Q9FJV5 | | Q9XIK5 |
| Q8TQF8 | | Q6NU40 | | O60304 | | Q7UQV4 | P06601 | P46358 | | Q7EZT1 | | Q58958 |
| O97552 | | Q03410 | | A4VCM0 | | Q9VPQ6 | Q08949 | P41896 | | Q5Z6B6 | | P96705 |
| O51804 | | Q9ZP54 | | P28938 | | Q9VUM0 | Q91573 | Q45618 | | Q9STF1 | | Q8SUB7 |
| Q57S50 | | Q75LH6 | | Q8GZM7 | | Q8I615 | Q38897 | Q9M347 | | Q54KJ8 | | O64702 |
| Q04649 | | Q7X9B9 | | Q3KRF1 | | Q6UDK2 | O34398 | O66659 | | Q00462 | | P40183 |
| Q9X5P2 | | Q9H4I2 | | Q9UNN4 | | Q9SY59 | P31367 | Q9LZ56 | | P53147 | | P13902 |
| C4KH08 | | Q8TCN5 | | P56270 | | Q9ZV43 | Q02953 | A3DH61 | | P0C7X2 | | O61016 |
| P24734 | | Q6P1N0 | | Q41558 | | P51979 | P39768 | Q8X229 | | Q8S9N6 | | P15556 |
| Q3KNW1 | | Q9DBR1 | | P19484 | | Q5A1A0 | Q92372 | Q22812 | | Q8IRC7 | | P24068 |
| A2YWA6 | | Q92771 | | Q6K6S5 | | Q86YC2 | P55610 | P27898 | | Q9SK55 | | P89479 |
| Q8MIC2 | | Q52KI8 | | Q02574 | | P51834 | O23661 | O17082 | | O31449 | | Q06260 |
| Q89551 | | O95644 | | Q06945 | | B9E1H0 | P40969 | Q6CHJ8 | | Q8LAU9 | | Q6AJ46 |
| C7GIZ9 | | O30477 | | P75539 | | B8CW13 | P04993 | Q8GZ38 | | A6NDR6 | | O14087 |
| A6QKF4 | | P08970 | | Q96C00 | | Q88WJ9 | Q08775 | Q9FGZ4 | | P43459 | | P33395 |
| Q9HAJ7 | | P37935 | | Q9FKR9 | | Q8KBS6 | O94395 | P78926 | | Q9LBW6 | | O68025 |
| O49595 | | Q9DG67 | | O93511 | | Q8REH4 | P53749 | O93748 | | Q8NA92 | | P44675 |
| C3MY51 | | Q8LPU5 | | Q0J0K1 | | B0M0P5 | P50070 | P23179 | | Q9FHR7 | | P37503 |
| P50775 | | Q96LU7 | | Q6B0B8 | | P08394 | O77245 | O16867 | | Q9SSW2 | | P00641 |
| P0CF67 | | Q38087 | | Q9XUB2 | | Q8TZY2 | Q9VLA2 | O43435 | | P15308 | | P54574 |
| Q62247 | | P26811 | | Q9D489 | | P12954 | P18733 | P55631 | | P39842 | | Q941D1 |
| P40641 | | Q336P2 | | Q9DAI4 | | P08956 | O31551 | Q8JN65 | | Q9IG42 | | Q8W191 |
| B4JQ42 | | P9WMG1 | | Q8H2D1 | | P34534 | A3BUD2 | Q88JX7 | | Q18273 | | P96653 |
| Q95153 | | P59966 | | P9WL37 | | O51578 | Q9PM37 | Q9SZ05 | | P10290 | | F9VN79 |
| P97500 | | Q9JHS1 | | Q93VB5 | | F4I3Z5 | O83505 | O60056 | | P26754 | | O51141 |
| P19028 | | Q15572 | | Q16600 | | Q3AA35 | C5M3V9 | P34303 | | P39015 | | P33786 |
| P18493 | | Q8IXK0 | | Q9PRE2 | | Q01371 | P20050 | Q97MB3 | | P13988 | | P22974 |
| Q2R482 | | Q9Y2L8 | | Q06433 | | P23477 | P47112 | Q9FV70 | | P03127 | | P50208 |
| Q9KRB2 | | O43196 | | Q13887 | | P93022 | P46394 | P24350 | | P76241 | | Q9L132 |
| P86938 | | Q923B3 | | A2WV68 | | P14727 | Q06455 | Q2J6D7 | | P17041 | | P04586 |
| Q61221 | | Q9JJY4 | | P48590 | | P34111 | Q09FC8 | Q5CD17 | | P25047 | | Q9T1U3 |
| Q7TMI3 | | P54253 | | Q9SUP7 | | O66878 | O44712 | F5HID2 | | Q02283 | | Q8KAM2 |
| Q90WU3 | | Q5SMU7 | | Q65956 | | Q4P6I8 | Q54ER9 | P37233 | | Q2V0P1 | | Q97W73 |
| Q14807 | | P07799 | | Q06546 | | Q4IJ84 | Q98QB3 | P53709 | | Q8LA53 | | Q5SI16 |
| O93120 | | Q32MZ4 | | Q652B0 | | Q59ZC8 | Q9ZUL3 | P55643 | | Q9LXV5 | | Q55244 |
| P63964 | | Q9Z726 | | O48772 | | P34228 | Q9HM14 | Q73P36 | | Q7XFU9 | | Q20561 |
| Q59S45 | | Q8IUM7 | | Q02638 | | Q70GP4 | Q60275 | B1W0D5 | | O66861 | | P56407 |
| Q1L8W0 | | Q9SF37 | | Q15306 | | D1KF50 | Q24142 | P41999 | | A3KNX5 | | Q8QHI2 |
| Q8N5A5 | | P15699 | | Q9STM4 | | Q5U263 | P31368 | Q97WM8 | | P32029 | | P52003 |
| P81067 | | P0A5U5 | | P78412 | | Q18LF9 | Q26228 | P39943 | | Q04832 | | Q4A666 |
| Q5NAW2 | | Q4R8W3 | | F4IPE3 | | Q9I920 | O78411 | O73823 | | P26586 | | P0A3R9 |
| P11161 | | P52379 | | P43651 | | Q196U0 | Q59189 | Q94JL3 | | C4VB43 | | O34777 |
| B3MZY6 | | Q66K89 | | P25696 | | Q9HK21 | Q5A220 | B8PBQ6 | | Q9ZVF5 | | O66475 |
| P35716 | | Q8CGW4 | | Q84LH8 | | Q21554 | Q86B79 | Q24537 | | Q93WV5 | | O22010 |
| P40426 | | A5E406 | | P9WKH7 | | F1QDI9 | Q6P1L6 | O22921 | | P0C6A0 | | Q9S763 |
| Q8EQ56 | | P25976 | | P56931 | | Q5D892 | Q22703 | Q67VL7 | | Q9LXG0 | | Q55433 |
| Q6AYD2 | | Q8CE22 | | Q02398 | | P36607 | Q92258 | B1ZN03 | | Q765S2 | | P96625 |
| Q39234 | | Q9PLE5 | | Q9Y7I8 | | Q1ZXA9 | P33417 | O42503 | | Q9LT67 | | Q7XSB2 |
| Q8TAU0 | | P48437 | | Q940I0 | | Q9LK10 | Q24849 | P23803 | | P42777 | | P20436 |
| Q03060 | | A2BGA0 | | Q59549 | | Q6P0N0 | P33655 | Q9FGF8 | | Q6QB00 | | O53509 |
| B0U178 | | Q9R1Y5 | | A8WWH5 | | P31380 | G5EBU4 | Q55CU7 | | Q6HN08 | | P97247 |
| Q9SZ70 | | Q9LW85 | | Q7XKC4 | | Q09228 | P78704 | Q12132 | | O14354 | | Q9FGX2 |
| A1YES6 | | Q8N187 | | Q96N77 | | P38144 | Q54K19 | Q8R890 | | Q9LZW2 | | Q9FRI0 |
| Q6RZN2 | | Q9Z7G4 | | Q17905 | | Q5JGV6 | P28159 | Q58793 | | P07760 | | O31672 |
| Q62232 | | O22208 | | P65982 | | O51568 | Q58113 | Q6Z0D2 | | Q10Q26 | | P75487 |
| Q06661 | | Q9JN65 | | P41768 | | Q5UQU6 | O94263 | Q32KV8 | | Q6Z0D9 | | Q9FYA2 |
| Q2G1N7 | | Q9FFI0 | | A1YEX3 | | Q9SPU7 | O02491 | P18752 | | P96690 | | P18853 |
| Q4JV96 | | A2AQ19 | | Q0WNR6 | | P07648 | P34522 | Q5Z807 | | O33812 | | P03671 |
| Q931H9 | | P50239 | | Q7XS74 | | Q03LF7 | P19705 | B6VQA1 | | C7J2Z1 | | P13434 |
| P27900 | | Q9H116 | | Q6ZPI3 | | Q64751 | C5BKL9 | O80358 | | P26343 | | P96582 |
| Q3LU41 | | Q5ZM39 | | F5HC97 | | P32644 | O42709 | O72902 | | Q8L4M6 | | P01549 |
| P03039 | | O54839 | | Q29CW0 | | P44945 | Q6FIN4 | P15025 | | P21649 | | A9JTY4 |
| Q5L6Z3 | | Q8BIQ8 | | Q5VYV0 | | Q08773 | Q9VEI9 | P42641 | | P33228 | | P06153 |
| P79775 | | P48972 | | Q9SA80 | | Q18034 | Q92804 | P08044 | | Q04296 | | Q89808 |
| Q06198 | | A9YTQ3 | | P41969 | | Q84LK0 | Q46802 | P55626 | | Q46942 | | O66625 |
| Q7A3C7 | | Q8UVQ4 | | B4F777 | | O77448 | Q9LZS0 | Q9C7P1 | | Q9SZ06 | | P96194 |
| Q2NEV4 | | B8BDQ4 | | Q502F0 | | Q89AB4 | Q96MW7 | O17657 | | P18756 | | P52665 |
| P45265 | | Q5ZKW8 | | P32579 | | Q5AMQ6 | P29555 | Q8RY99 | | Q10007 | | O07586 |
| B3QLX3 | | P19323 | | P17208 | | Q9SMV7 | Q05215 | Q8GW05 | | P81394 | | P40626 |
| P18743 | | Q10M00 | | Q8C6P8 | | Q5A4G2 | P19704 | Q9SVQ0 | | O49255 | | P50209 |
| Q7A3W9 | | P10074 | | Q6NT76 | | P40798 | P34233 | Q5Y2C3 | | O97039 | | Q6XZ05 |
| B2SB45 | | A6QR00 | | P61496 | | A3EWL3 | P41828 | P21940 | | O81788 | | Q65XK1 |
| Q5HI00 | | Q69QA6 | | Q8LPR5 | | Q9JI57 | Q12308 | P56260 | | Q05943 | | P42103 |
| Q2FEJ8 | | P18850 | | Q9FHM5 | | Q3U0P1 | P40535 | O74747 | | Q021H8 | | Q10574 |
| A7ZZ97 | | P9WMR4 | | Q5RKG3 | | Q9H2P0 | D4A7U2 | F4KGY6 | | B9KKS1 | | B0R4X4 |
| P10070 | | Q9I5W0 | | Q4FZR5 | | Q56830 | Q84VG7 | Q9NR83 | | Q5QMM3 | | P41055 |
| F5HDQ6 | | Q9CWW7 | | Q96E29 | | J3F2B0 | Q9C882 | P27552 | | Q84YL3 | | Q07928 |
| Q85428 | | Q01167 | | Q7EXZ2 | | P06777 | P13894 | Q9CAR4 | | Q9C633 | | Q9SLG0 |
| Q2URJ5 | | Q5Z8Q9 | | Q9UIH9 | | Q58815 | P40151 | P55615 | | P19653 | | Q55C24 |
| Q91187 | | B0X9H6 | | Q8L5W8 | | Q54UG3 | P07548 | Q9LT81 | | B2LXS7 | | Q8TV90 |
| Q04861 | | Q96GC6 | | Q01KM7 | | Q24478 | O27276 | P15840 | | Q1WG82 | | Q9WZ73 |
| Q4A127 | | Q3U133 | | P60230 | | Q9LYD9 | P09932 | Q96PM9 | | P41772 | | P54182 |
| P84403 | | Q1HVF7 | | Q9Z2E1 | | P38114 | Q84WU6 | Q5AMM4 | | P45876 | | P21260 |
| P35712 | | Q91WC1 | | P56143 | | P25302 | Q6CCK2 | Q9FNY2 | | P06774 | | P06022 |
| Q01714 | | Q9FFY9 | | A6NKF2 | | Q8W103 | P78978 | O18326 | | Q9FUY6 | | O06008 |
| P06839 | | Q5TJE2 | | B0R4P0 | | P28349 | Q10096 | Q7SYB3 | | Q99142 | | Q03182 |
| P61875 | | O93257 | | Q63934 | | A8MQ14 | Q9SJJ3 | P71344 | | Q07956 | | P27336 |
| Q2H6C5 | | Q10Q08 | | Q969G3 | | P48786 | P09145 | P34161 | | P77634 | | O06474 |
| P08199 | | Q59ZZ6 | | P27278 | | P38078 | Q6FV94 | P41894 | | Q4PSU4 | | Q05007 |
| O72907 | | P52739 | | Q4JQW5 | | P40352 | Q5AL16 | Q7K0S9 | | O74796 | | P05662 |
| Q8VEE4 | | O57863 | | Q9WUU6 | | P40349 | P56095 | Q9XI36 | | O41056 | | Q9LTC6 |
| Q63503 | | P32519 | | P09274 | | O65607 | Q18192 | P36702 | | P06847 | | Q9LTC5 |
| F7BLM1 | | Q9JLZ6 | | Q96H86 | | Q9NJB5 | P14870 | O15315 | | Q96C55 | | Q5F882 |
| F1MF21 | | Q89AB2 | | Q8S3D1 | | Q55BR6 | Q04089 | Q9SYG2 | | B0R9X6 | | P52953 |
| Q8U0K8 | | O95831 | | P41218 | | P89105 | P32152 | O02305 | | Q700E3 | | Q9FNV8 |
| Q91910 | | Q02086 | | Q965W2 | | Q02308 | Q10076 | Q9M4A1 | | A4FQ42 | | O04027 |
| P70459 | | P48988 | | Q9FX53 | | P49869 | A2WM14 | P27159 | | P15238 | | Q74MZ3 |
| P18146 | | P49881 | | P28958 | | O13807 | Q96UP6 | Q03935 | | Q9LZV6 | | O31497 |
| Q02780 | | Q3MHN7 | | Q9QZW9 | | P17255 | O60174 | G2TRT1 | | P05552 | | P70993 |
| Q1W1G1 | | Q3KSS7 | | Q99090 | | P55197 | P34450 | Q8L9Y3 | | Q5AFP3 | | O34527 |
| Q9CXC9 | | Q8N3X6 | | O88621 | | A7UXD4 | Q08605 | P22809 | | Q84513 | | Q75JQ9 |
| P23759 | | Q9UGU5 | | O49403 | | P12383 | Q9H930 | Q24533 | | P39360 | | Q8SWA6 |
| Q55081 | | Q9PLC9 | | P48581 | | Q9W0K4 | P15620 | P76102 | | Q9ZPW7 | | Q02835 |
| Q9JKQ4 | | Q92908 | | Q15583 | | C5E4G1 | Q6CK33 | P36981 | | P52744 | | Q97FU2 |
| Q9AK82 | | Q7Z7K2 | | P80011 | | Q9ULJ3 | Q09587 | P53035 | | Q8NB15 | | P86252 |
| Q3KR53 | | Q03989 | | Q9SEZ3 | | P22373 | Q17370 | P22148 | | Q65WX1 | | Q09821 |
| Q0P569 | | Q9BTC8 | | Q61179 | | P46676 | Q9GTD4 | Q9SM27 | | Q9SIE4 | | P28816 |
| Q0IH98 | | Q5R638 | | Q44406 | | Q9I9K0 | Q01201 | P01104 | | Q94166 | | P25279 |
| Q4R4M6 | | Q02395 | | Q5HZ36 | | P34739 | Q5W1J5 | O16966 | | A5G742 | | P37510 |
| Q68EL6 | | O08789 | | Q04088 | | O73954 | Q9VQS7 | A0L3I9 | | Q08398 | | P96158 |
| P55055 | | Q6PQQ4 | | Q6BEB4 | | P49413 | Q24180 | P07676 | | Q9YDF6 | | Q5ZM20 |
| Q6NRM0 | | Q651U1 | | Q0VG99 | | Q19546 | Q9F5P4 | P36784 | | Q9SX99 | | P04537 |
| P12540 | | P19893 | | P28813 | | O05052 | P05527 | Q09184 | | P03698 | | Q55963 |
| O54743 | | P9WHJ0 | | Q32SG4 | | P28166 | F1R3W0 | P01102 | | Q42430 | | P94578 |
| P26630 | | Q9Z7F0 | | Q8S9L0 | | O95625 | Q9LZM8 | Q54PJ7 | | P06946 | | Q8NE65 |
| A2YMR0 | | P97863 | | Q9Y5K1 | | Q4PD88 | P56721 | O42934 | | P68349 | | P52675 |
| Q9FFX1 | | A6YP92 | | Q9NA56 | | Q384Y0 | Q39117 | P38071 | | P23774 | | P07059 |
| O60663 | | P38530 | | Q9FWX2 | | Q55F37 | O07570 | P20268 | | A2Z0Q0 | | Q3SZB8 |
| P50481 | | A4FVP6 | | Q24645 | | Q6K5K2 | P34664 | Q00461 | | Q1KKR8 | | O28314 |
| Q6L4H4 | | D8Q8R5 | | B4IB36 | | O75362 | P17098 | Q95QY7 | | Q08595 | | P96652 |
| Q9YE67 | | P08062 | | Q9ZWA1 | | P39720 | O67779 | P46330 | | O51395 | | Q25214 |
| Q9QZ59 | | Q810A1 | | O60870 | | Q54ED2 | Q9Y7G2 | E9PGG2 | | P77732 | | P87294 |
| P57722 | | Q49166 | | P29548 | | P14083 | Q9LDD4 | Q9C690 | | P39361 | | Q6ZRF7 |
| A4SC23 | | P43016 | | O18086 | | Q6PGB8 | A3M0E6 | O27636 | | Q00492 | | P43463 |
| Q5L648 | | Q8IX15 | | P35881 | | A7U6F3 | Q6BJ51 | Q54JH6 | | Q6X7K1 | | P06966 |
| Q8S7W9 | | Q5FWU5 | | P40796 | | Q6PJG2 | Q9AWB2 | Q90415 | | Q9GM03 | | P32103 |
| P03001 | | P03710 | | Q96FI4 | | P0CAP5 | Q96I24 | Q5JNS0 | | P36930 | | Q97YX6 |
| O94697 | | Q5TJF7 | | Q8X7R5 | | E7F888 | Q19345 | Q9FNG6 | | A5FEP6 | | Q08824 |
| A6NHT5 | | A6NJT0 | | P34877 | | O49498 | Q8NDF8 | Q5A4N5 | | P09009 | | P32445 |
| P40348 | | Q3U381 | | P28875 | | P17040 | Q6AZ28 | Q6X7J4 | | Q9LP56 | | P23541 |
| Q8R4E6 | | Q5UQQ9 | | P41453 | | Q60295 | O22056 | Q9T0D3 | | P32104 | | P30735 |
| Q8WVM0 | | P35637 | | Q810F8 | | Q6NXK2 | Q9ZVX2 | Q93348 | | P94591 | | P72979 |
| Q15853 | | Q66JW3 | | Q9LQM3 | | Q6YUL8 | Q9RWR5 | Q8MYF0 | | P53551 | | P44558 |
| P43345 | | Q9WVG9 | | P16557 | | P10486 | Q4H3P5 | Q28590 | | Q9BDJ7 | | Q58177 |
| P0A9F4 | | Q9BZE0 | | Q49434 | | Q12180 | P53695 | Q48AS5 | | Q23697 | | P45277 |
| A2XDD6 | | P49777 | | Q3V4U8 | | Q851V5 | Q54C22 | Q45620 | | P39650 | | P20269 |
| P10036 | | Q66HA2 | | Q6DD87 | | O14709 | O24621 | Q55BA5 | | P26354 | | P75679 |
| P0CF79 | | Q9TX44 | | Q10GM4 | | O61735 | Q9U405 | P06602 | | Q9FJA2 | | Q8PW88 |
| A1YF08 | | Q9UBZ4 | | P29303 | | P33244 | P94544 | P10075 | | O95231 | | Q93PU7 |
| Q6IP18 | | P51449 | | P21711 | | Q6FPE6 | P45897 | O16391 | | O05261 | | Q8BDG7 |
| P17433 | | Q6ZN18 | | Q3SY56 | | Q4U3V0 | Q8NCP5 | Q56A18 | | Q9SJG4 | | Q9FX77 |
| P0AD02 | | Q9BQA5 | | Q28FE5 | | Q6C2R8 | O14108 | P75431 | | Q40359 | | Q9N0M2 |
| Q01068 | | Q90X44 | | P9WMB2 | | P33339 | Q08518 | Q93WI2 | | P09774 | | P97876 |
| P66797 | | Q9SB79 | | Q700W2 | | Q7KUT2 | P23792 | O02073 | | Q9HG13 | | P38877 |
| P20098 | | P49643 | | Q9C7G4 | | Q9U3V5 | Q37989 | Q8L4E7 | | Q7XBH4 | | Q8SVT3 |
| O60682 | | P11831 | | A2YXQ1 | | Q24119 | O65590 | Q8L7N8 | | Q9Y1B2 | | Q57920 |
| P70827 | | Q46087 | | Q24648 | | P33537 | Q9NW07 | Q03444 | | A0R2D4 | | P13976 |
| P29596 | | Q5ZJL7 | | B3H5A8 | | P41511 | Q5T619 | O04017 | | P15082 | | P71036 |
| P36806 | | A7KBS4 | | O81037 | | P23009 | O23116 | Q2SQZ4 | | Q9XIP5 | | Q8N726 |
| P52161 | | Q9LVQ7 | | Q9SK74 | | P53091 | P78871 | F4IQL7 | | P0CB25 | | Q8STR8 |
| Q9V2D7 | | Q96CS4 | | B5DR03 | | Q9FYG2 | Q20977 | Q8L627 | | Q01108 | | P53547 |
| P28042 | | Q64249 | | P9WGG4 | | Q9S1G2 | P52890 | Q90718 | | Q6CB23 | | P17502 |
| Q5XJ97 | | Q1HGE8 | | Q9S7Y1 | | Q6GZR5 | O13329 | Q6RFH8 | | Q9M8M5 | | Q55940 |
| Q99VT5 | | P26408 | | Q9H4Q4 | | Q9LKZ3 | Q05534 | Q9S7U5 | | Q94165 | | P87394 |
| P06555 | | Q9Y2Y4 | | Q93V43 | | Q9P1Z0 | F5HIC6 | Q0J6N4 | | A5G743 | | Q46864 |
| P81195 | | Q0VC30 | | P23768 | | A7TSV4 | P06519 | O16425 | | Q8ZYL6 | | Q90ZL1 |
| Q2KS10 | | P97360 | | Q9LD44 | | P41846 | Q9X1G3 | Q9UTL5 | | Q9LVU8 | | Q9XSB7 |
| Q12873 | | Q9C0J9 | | Q9UPM6 | | Q6CIL8 | P05066 | Q9C9F0 | | P23393 | | Q8CNK0 |
| P0CE41 | | Q9GL32 | | Q83NZ4 | | O74458 | P49412 | Q5UQS2 | | P46106 | | P06627 |
| A1L1R6 | | P94426 | | Q13351 | | P25992 | O14335 | Q9H7X3 | | Q65X92 | | O27346 |
| Q5RE34 | | Q86VK4 | | O02316 | | Q9Y802 | Q10134 | Q8S3D2 | | P29073 | | Q9PQK9 |
| Q9R0G7 | | P19474 | | P20046 | | Q54LU5 | P25364 | Q5W6R4 | | D4GYE7 | | Q8SRE2 |
| I0IUP4 | | Q92753 | | P03023 | | P15330 | Q9N5D6 | P15370 | | Q75WM6 | | O53838 |
| Q66611 | | O01931 | | Q01JD1 | | Q80WQ8 | F4K2R6 | P91829 | | Q700D9 | | Q708A7 |
| Q6PCR6 | | Q0E3F8 | | A2A9I7 | | E5AW45 | Q10144 | Q9FUA4 | | Q8CSL5 | | Q08082 |
| Q6ZQJ5 | | P10828 | | Q9LFQ9 | | P74933 | Q84JT7 | Q54RZ9 | | Q9XEF0 | | O34712 |
| E1B7X9 | | Q84TK1 | | O95600 | | P38485 | Q6XDT4 | Q9FM03 | | P14984 | | O52029 |
| O36363 | | P42167 | | B9DI20 | | P52960 | Q8U3J2 | P32028 | | Q8F874 | | P23848 |
| P08504 | | Q3B7U2 | | Q6Z528 | | P09804 | Q7KSD8 | P27609 | | P54717 | | P56269 |
| Q3YC04 | | Q3SWU4 | | P07674 | | P05468 | Q02457 | Q9HGP0 | | Q93352 | | P73145 |
| Q9X909 | | P24116 | | A2XMN1 | | P0C1D3 | P04522 | Q0IHI7 | | Q9LW00 | | O57568 |
| Q8BY46 | | O43829 | | Q2QMT2 | | B7ZQJ9 | P40209 | Q682D3 | | P10726 | | A0QRY6 |
| Q0V9Q6 | | Q9FNZ1 | | Q60668 | | P26359 | Q9XYB7 | Q9LK48 | | P40847 | | O28051 |
| P03162 | | Q75EN0 | | B3DQV1 | | Q57YG0 | O60131 | Q2RV90 | | P34684 | | Q8SSG3 |
| P41512 | | A2XFC1 | | P06134 | | Q8I7Z8 | Q8N2I2 | O22264 | | Q8TWK3 | | Q07053 |
| Q9PEV8 | | P54274 | | Q91VJ1 | | Q6CHE5 | Q6C764 | O17927 | | Q6J9S1 | | Q04580 |
| Q9EQB9 | | Q9NP62 | | Q9FK12 | | Q8TUT0 | P07269 | Q8GXH3 | | Q12MQ7 | | P96673 |
| Q8NYT6 | | Q01194 | | Q84WK0 | | G5EDG2 | P31370 | Q26417 | | P35878 | | P96631 |
| Q8FJN1 | | Q99J79 | | Q84JQ8 | | O18017 | Q5A4X5 | P28519 | | Q10GP0 | | Q7KRI2 |
| Q653H7 | | Q6U8D7 | | Q96B01 | | O13339 | Q6PQQ3 | Q96C28 | | Q9LRJ4 | | O26703 |
| Q6GL57 | | Q8BXG3 | | Q9LSL1 | | Q55971 | Q18919 | O77051 | | Q5PP65 | | Q47274 |
| Q2MHH4 | | Q9H171 | | Q9ZUG9 | | P35600 | Q6NQ88 | P23757 | | Q69KP0 | | P33788 |
| Q1BYA7 | | Q02556 | | P36547 | | P12689 | Q9FVQ1 | Q9SK02 | | Q9QPN4 | | Q60386 |
| Q90867 | | Q0V8G8 | | Q8TFU8 | | Q8VY05 | P43609 | O81791 | | Q0K846 | | P50337 |
| Q62431 | | P39022 | | P10181 | | Q0P897 | Q93WV0 | P03267 | | Q06182 | | Q06259 |
| C5MCK5 | | B2RXF5 | | Q9NP08 | | Q9XYD3 | O94720 | Q9ZPY0 | | P29506 | | Q9T1Q8 |
| Q9Y692 | | P18754 | | Q9FMF4 | | O74773 | Q94545 | O44960 | | P77721 | | Q3E7N5 |
| A4FUF0 | | Q9WUQ2 | | Q15329 | | O63852 | P11292 | Q77MR9 | | P09090 | | Q54HV7 |
| P03264 | | P15976 | | Q6PV68 | | Q9RXB5 | P55878 | Q9CAQ8 | | Q9ASX9 | | Q8SW51 |
| P41153 | | Q16254 | | Q93WU8 | | P25502 | P34205 | P96629 | | O31713 | | O93774 |
| P31266 | | Q6PD05 | | Q94AN4 | | C5DGQ7 | Q54WV4 | P25042 | | P46337 | | P30346 |
| A6H7J1 | | Q9H161 | | Q9PSY4 | | P41508 | Q38914 | O31517 | | Q9Y9V7 | | Q92399 |
| Q8GYC1 | | Q99089 | | Q12952 | | P06701 | P03373 | O35652 | | Q8H2D0 | | P26768 |
| Q6XBJ3 | | Q8BZ89 | | P35227 | | Q9W0K7 | P27536 | Q707Y0 | | P03856 | | Q57988 |
| Q58NQ5 | | Q7SDY3 | | O95076 | | Q54HX6 | O13988 | Q9SPG3 | | P17890 | | P37486 |
| Q7NMD1 | | Q61817 | | Q8GX84 | | Q1MTM9 | Q8I8V0 | Q25604 | | O31654 | | P15142 |
| O00409 | | Q3KSU3 | | Q6L5G1 | | Q54IJ9 | P05846 | Q9C5G0 | | Q6H509 | | Q94045 |
| P11308 | | Q9C009 | | Q9SUQ2 | | P33200 | Q07231 | Q5UQP9 | | Q9SJ98 | | Q8BDG6 |
| Q9PUK5 | | Q0VCU0 | | Q02991 | | P39881 | P14064 | Q6NKN9 | | O34449 | | A1SDH7 |
| P19544 | | Q6AXY9 | | P35168 | | Q24432 | P03632 | Q9US51 | | Q91EK1 | | Q05095 |
| C5CH91 | | P0CB48 | | O00321 | | Q9Y794 | P27558 | P36093 | | Q752S4 | | Q86WV5 |
| Q2YDI2 | | Q86VE0 | | Q7Y0W3 | | P30189 | P10479 | Q570R7 | | Q942D6 | | P17909 |
| P0CJ85 | | Q4V7E1 | | Q61467 | | P24279 | Q9C519 | B1ZSD3 | | P28174 | | Q02123 |
| B4JND4 | | Q05738 | | Q17QR5 | | B0G0Y5 | Q28EG9 | Q02360 | | P42968 | | Q9RZ89 |
| P22015 | | Q5PP28 | | Q9ZV33 | | P21657 | P02836 | Q9PTK2 | | Q8ZWG7 | | Q708A1 |
| Q1LWL8 | | Q5JT82 | | Q94F58 | | Q96FC9 | Q01663 | Q726D5 | | P76161 | | P22896 |
| O94160 | | Q8K0S9 | | Q93ZH2 | | Q867Z4 | Q02479 | Q948L5 | | Q9PQS0 | | P25211 |
| Q9BWW4 | | P83950 | | P9WMJ3 | | Q04222 | P42944 | Q02080 | | Q8ZWT6 | | P28267 |
| Q6VFT5 | | P9WGJ8 | | Q96MN9 | | P33538 | Q4JC68 | Q9FNY7 | | P05052 | | Q52620 |
| Q5XFQ6 | | P0CF88 | | Q9FH57 | | Q8G4H3 | P41046 | Q00265 | | P51984 | | Q01751 |
| Q84MN7 | | P68462 | | P9WPJ4 | | Q63HK3 | Q54DA1 | Q0B0Z2 | | P44780 | | P30340 |
| B2HZA5 | | Q7TNV0 | | P25858 | | P40467 | P23023 | O17933 | | Q9UTM0 | | Q9FFK0 |
| O35627 | | Q5J3Q5 | | P41779 | | P30319 | P35201 | P49997 | | P28361 | | P49886 |
| Q5NVR0 | | Q9LUM4 | | Q9SGS2 | | Q54PN5 | P07387 | Q700C7 | | Q38850 | | P40569 |
| P03409 | | O65508 | | Q7LYW4 | | O74412 | Q6NT04 | Q86UQ0 | | Q9YEZ5 | | Q8SVT6 |
| Q934T6 | | Q9KBQ0 | | Q9ZPW3 | | Q9V5M6 | Q9LXG8 | Q9FL40 | | Q74MV1 | | Q98VP9 |
| A5PKG8 | | P15263 | | Q75LX7 | | P34427 | P39736 | Q00401 | | O10308 | | P36394 |
| Q10M29 | | Q9MA17 | | P18715 | | Q10103 | O74944 | Q1PFR7 | | O13852 | | P54590 |
| P92953 | | Q8CGW9 | | Q9HTH5 | | Q9USQ1 | Q9LD95 | Q3JF36 | | Q72H90 | | Q9Y812 |
| C0HIA3 | | Q9S7B2 | | Q9SKT1 | | O93745 | O74308 | Q3A8M6 | | P76034 | | Q8LC59 |
| Q9H9S0 | | Q5ZA07 | | O82132 | | Q8XRH0 | Q5IFN2 | P75279 | | Q9SVL0 | | O69711 |
| A1C9M5 | | Q7XJK6 | | O95238 | | Q9UHL9 | Q08904 | Q8H102 | | P26275 | | A6X980 |
| Q9SZP1 | | Q84WP6 | | P37076 | | P25846 | C5DX31 | Q9FRH4 | | Q2HIV9 | | O52026 |
| Q59592 | | Q45968 | | Q9FIW9 | | Q84TH9 | O32006 | Q6Z1Z3 | | P13483 | | P15081 |
| P03568 | | C1CW06 | | Q66LM6 | | Q6YYC0 | Q5UPI0 | P10030 | | Q9CAN9 | | Q9T0H7 |
| Q66406 | | O75683 | | P97503 | | Q7TN75 | Q96AP0 | Q9M4A2 | | P26488 | | P21523 |
| Q04741 | | B5ZBT4 | | Q8FQS2 | | A7TE38 | Q8GT73 | P21514 | | P51007 | | P28009 |
| Q46967 | | Q8VYJ2 | | P62572 | | P32504 | Q4I0C5 | O42953 | | Q67UX6 | | O05023 |
| Q8EUW6 | | Q5IS79 | | O60902 | | P52027 | Q6NSZ9 | Q6ZN32 | | Q51872 | | O31679 |
| P29248 | | Q4P400 | | Q6AZW8 | | Q54YV4 | P16375 | P79779 | | O04682 | | Q8UAA8 |
| Q2G1T7 | | Q9Y5W3 | | O43638 | | Q9C587 | Q755A0 | O82277 | | P42736 | | Q45881 |
| P0A3H7 | | Q8BIA3 | | P75435 | | Q9LKI5 | C4R7Q5 | P49870 | | Q9FIX5 | | Q5AL63 |
| P69409 | | P24849 | | P0CX05 | | Q8IN81 | O85057 | P28482 | | Q9SVC5 | | Q47588 |
| P0AEL9 | | Q12972 | | Q9SR07 | | Q0D5G4 | P41934 | Q8GWP0 | | Q9YFT8 | | Q54YS0 |
| P25210 | | P23823 | | Q17295 | | P22265 | P22810 | Q9FYJ2 | | Q03316 | | Q07683 |
| Q64124 | | Q8GUC3 | | Q9FG68 | | Q38796 | P13469 | Q9FME3 | | Q93899 | | P33647 |
| Q99TA4 | | O43316 | | Q9T072 | | C0SP86 | Q17771 | Q66GR3 | | P25476 | | P36611 |
| Q51506 | | Q2NF79 | | P0A4T1 | | Q65XV7 | Q6PB75 | P49379 | | Q84K52 | | P04889 |
| Q5JHH2 | | Q9CXE6 | | Q84JS6 | | Q04052 | P33224 | Q9VWW0 | | Q03973 | | P45902 |
| C3N649 | | P9WNT0 | | Q6D0X0 | | A1Z9E2 | Q6FUG1 | P00969 | | Q8EQQ3 | | P0C7L5 |
| E1BD44 | | Q8IZA3 | | Q9FL26 | | P50104 | O16850 | Q9IAC6 | | P19433 | | P13657 |
| Q2FVY9 | | Q9LST3 | | P0CI75 | | Q6Z3U3 | Q9N658 | Q6MAG9 | | Q27850 | | Q8JN70 |
| P52926 | | O28401 | | Q53WG8 | | O70736 | Q3U3N0 | Q9FYG7 | | Q9Y7K9 | | Q46561 |
| Q57220 | | Q2RW61 | | P25233 | | O22864 | P38704 | Q53198 | | Q9SSW1 | | C0H3Y4 |
| O51418 | | P43161 | | Q9SV15 | | Q59VQ8 | Q9SV09 | Q09452 | | P37335 | | Q9J5B7 |
| Q9AKK7 | | P9WMJ1 | | Q9NGB4 | | Q9N4H4 | P40791 | Q6GR30 | | O65036 | | P18867 |
| Q25515 | | P37956 | | Q9SUP6 | | P87114 | P52168 | Q9SAK5 | | Q00096 | | P41249 |
| P13123 | | O17931 | | Q94LW4 | | P91868 | O94416 | P47175 | | Q9ZWA2 | | P27556 |
| Q7KU24 | | P55733 | | Q9SNB4 | | Q03631 | O66893 | O34336 | | Q9FZ90 | | Q01936 |
| Q6CA87 | | Q9FLJ2 | | Q00338 | | Q869W0 | Q03513 | Q9UJN7 | | B7XIV9 | | P0A3S0 |
| Q9Z330 | | B3QM22 | | P62559 | | Q10328 | Q6C0U2 | Q8K1L0 | | Q9LIN3 | | P23789 |
| A7TJI3 | | P58195 | | Q9SDW0 | | O13921 | O14283 | Q09732 | | P40574 | | Q9Z9W1 |
| P03362 | | P39343 | | Q8NCA9 | | Q5NB82 | P05845 | O59851 | | Q08182 | | P49536 |
| O70133 | | Q9K6K2 | | Q99NA2 | | P39521 | Q24423 | Q6IUP1 | | Q8TF63 | | P09589 |
| Q8ZQD5 | | Q8H0W3 | | P19257 | | Q0JC27 | Q9UST5 | P08342 | | O80917 | | P39213 |
| P43139 | | O55230 | | Q00577 | | P23914 | Q9Y2P0 | G2TRN9 | | O80654 | | O34419 |
| Q04AN7 | | P43472 | | P06709 | | O94623 | Q9SJ41 | Q9Z3R4 | | P29176 | | P97273 |
| Q6BIP2 | | O09113 | | P9WMF6 | | O60182 | P49882 | Q68G74 | | Q8SS62 | | Q7AKI9 |
| Q6E2N3 | | P09088 | | Q8S3F1 | | Q1ECW2 | Q20765 | O76828 | | O22230 | | Q1G3Z6 |
| Q0D9R7 | | Q13461 | | Q9LTC7 | | D0ZLR9 | Q59VM4 | Q08142 | | Q6FK91 | | O34647 |
| A4HVU6 | | P9WNU3 | | Q9C7T4 | | Q54PT9 | Q6LA43 | P07665 | | Q8AXQ4 | | O34844 |
| Q759V4 | | Q32L68 | | Q8KUV2 | | P54277 | Q01012 | A0AQW4 | | Q9SIB4 | | C0H3S6 |
| Q84173 | | Q9C932 | | Q99697 | | P54394 | P41830 | O64647 | | Q9SIC3 | | P96677 |
| Q80YR5 | | P52662 | | Q7L3S4 | | Q54BD4 | Q19196 | P04011 | | O86029 | | P49885 |
| Q5TM61 | | Q9SR17 | | Q9M374 | | P10582 | P49867 | P55729 | | Q9SGE3 | | O85264 |
| D3UEC9 | | Q94LW3 | | P42774 | | Q384Y1 | P15863 | P78962 | | Q54UM1 | | O35034 |
| F1QZ88 | | P46667 | | Q22292 | | Q580X6 | O14258 | P22058 | | C4V6H6 | | P29773 |
| Q6DD04 | | P0ACQ7 | | P36674 | | P87000 | Q9P6R8 | O81242 | | Q88886 | | Q9XG56 |
| B5VML1 | | Q9HIH6 | | P46924 | | P32896 | Q00400 | Q9S7W5 | | Q44929 | | O74807 |
| Q0SM54 | | P9WMF2 | | Q64662 | | P32797 | Q9WUB5 | Q8N8C0 | | Q02516 | | O88368 |
| Q01778 | | Q96IQ9 | | Q9LSD5 | | P30314 | Q9VPH2 | Q7T6Y4 | | P40628 | | P10590 |
| A0MS83 | | Q7GDL5 | | Q9U2C9 | | O16810 | Q0JKK6 | P25032 | | P24061 | | O68891 |
| B4JYN3 | | Q9C5T4 | | Q8LR86 | | O23463 | Q28811 | Q6C5G3 | | Q03474 | | O97965 |
| Q8N3S3 | | P67664 | | P13771 | | Q9WVL2 | Q0J235 | Q8H0V5 | | P41995 | | Q9VLR5 |
| P25425 | | Q7XPK1 | | Q9M9S0 | | O42861 | P38035 | Q1ZXF0 | | O54452 | | Q45666 |
| Q3U0V1 | | Q8VZP4 | | A1KNB8 | | P73077 | Q92259 | P03343 | | Q9N1Q5 | | P29927 |
| Q9H3D4 | | Q8LL11 | | P0A4T6 | | Q84WP3 | C4QV17 | Q01214 | | Q9LEZ9 | | A3A463 |
| O00571 | | Q01610 | | Q9UD57 | | P11637 | O32138 | P24426 | | P14233 | | Q65ZA6 |
| P14294 | | Q6AYI4 | | Q8RWN5 | | P30181 | Q03465 | Q3A8P5 | | Q057N0 | | Q8GY11 |
| Q05153 | | Q89AD1 | | Q19203 | | P41696 | Q9NV72 | Q9STX0 | | Q9FMY7 | | Q92GC8 |
| Q9CCG2 | | P22335 | | O22768 | | Q12151 | Q96EG3 | Q8RA66 | | Q8LDF9 | | P81315 |
| P52308 | | P0A9G3 | | P0ACR3 | | Q9M041 | Q54Q90 | Q24JK4 | | P22405 | | O31494 |
| Q9EPZ6 | | Q14863 | | Q96MM3 | | Q24573 | F4KFT6 | Q04116 | | P16684 | | O52028 |
| B5VEB9 | | P03695 | | E1WAC0 | | Q8EER3 | P78714 | Q9JMT8 | | Q02685 | | P15017 |
| Q5XXA9 | | P07903 | | P74422 | | Q2HR73 | Q7XSS9 | P15276 | | Q54KT7 | | P15417 |
| Q05913 | | P0A4T3 | | Q9BT49 | | Q8TF20 | Q11178 | Q9SD61 | | O31761 | | P52695 |
| Q9WV25 | | A2YNY4 | | P0A247 | | P52958 | Q9LEZ3 | Q97YC1 | | O34901 | | P10178 |
| B4PPK2 | | P0CL09 | | P29169 | | Q54MA9 | P40408 | Q8LPH6 | | P13393 | | P18866 |
| Q8VBU8 | | P0ACR6 | | P13545 | | Q54HP1 | Q2QTY2 | Q9C688 | | Q9SN74 | | P87057 |
| A2VD01 | | P75403 | | Q84U86 | | Q9LVX1 | P45264 | O83049 | | Q9FYE6 | | Q82XQ7 |
| P78413 | | O25758 | | Q9SYH4 | | O51498 | P54791 | Q9SB80 | | P24290 | | Q46560 |
| P15336 | | Q01105 | | P0ACR8 | | Q59RQ0 | O58001 | Q9KI19 | | Q08821 | | O31943 |
| Q58DJ0 | | P46118 | | Q9FJ00 | | Q59RR3 | P75438 | P25294 | | P13669 | | O32238 |
| B5VQH0 | | Q9PWD6 | | Q08597 | | Q24174 | Q9HE16 | Q2R3Y1 | | Q9HSL7 | | O31844 |
| P10157 | | P0ACI2 | | P35319 | | P07272 | Q9ULM2 | Q9HPW0 | | P11257 | | P00214 |
| P09086 | | Q08DY6 | | P70785 | | Q0JDM0 | Q9P7P2 | O22812 | | P29552 | | Q8SVD3 |
| Q05501 | | O70191 | | Q7F2L3 | | Q9H5L6 | Q5RBN8 | P17106 | | P49451 | | Q8SW50 |
| Q80WT2 | | Q97MK5 | | Q8ZZU1 | | O51157 | P20457 | P20271 | | P25084 | | P10179 |
| Q03828 | | Q9FNI3 | | Q8H1G0 | | Q12175 | O23076 | Q8REB0 | | P52108 | | P83211 |
| Q8BG99 | | Q801F8 | | P26316 | | Q9UUD1 | P26370 | O35719 | | P49953 | | O53478 |
| O00716 | | P12980 | | P0ACH9 | | P32863 | Q9USY4 | P16922 | | P44207 | | P50210 |
| Q6PF39 | | Q5KQI4 | | Q8VZ22 | | Q54GH2 | P70486 | Q6X7K0 | | P77601 | | O65154 |
| Q6CQ86 | | Q9LL85 | | Q94C69 | | Q7SQ98 | A3CEM4 | Q9YHT3 | | Q84T68 | | P03045 |
| Q924A0 | | B9K4A6 | | Q9EXL7 | | Q03833 | Q75A64 | P23922 | | P46908 | | Q57089 |
| Q9KT84 | | P03934 | | P14353 | | Q86HX9 | Q7XJU0 | Q05640 | | O65515 | | Q57807 |
| P54090 | | P18846 | | O80341 | | Q9M1B0 | Q8VIH1 | O55087 | | P41546 | | Q93K57 |
| Q9UMR3 | | Q8TAK6 | | P35112 | | Q9VK71 | Q9NZV7 | Q17241 | | P38564 | | P0C230 |
| Q64163 | | P0ACK4 | | Q8X4M5 | | Q54RC3 | P13682 | Q9HEQ9 | | Q9SFD8 | | Q90523 |
| Q8FA34 | | P12923 | | Q9NLC2 | | O16102 | Q8W2F1 | Q6J9Q2 | | Q94DH3 | | Q58335 |
| Q2FKF1 | | P32787 | | Q87ZG4 | | P28348 | Q5A7S7 | Q9FLX8 | | P39796 | | P45949 |
| P13623 | | Q38847 | | Q6EUG4 | | P20189 | P34219 | Q2FQQ2 | | D9J034 | | O83278 |
| A4UTQ0 | | P64159 | | A5F9F9 | | Q96KM6 | O59830 | O16604 | | P31453 | | Q9KMG4 |
| Q9F407 | | P75416 | | Q9UH92 | | P42283 | Q941Q3 | P52698 | | Q8WTZ3 | | P09634 |
| Q02962 | | P67854 | | Q9CA66 | | Q6FJJ1 | Q01791 | Q8XTL6 | | Q97Y88 | | Q9KMA5 |
| P25490 | | P43464 | | P34818 | | Q38X69 | Q9LME6 | Q9NLA3 | | O05509 | | Q06552 |
| B3F209 | | Q9UKW6 | | P55576 | | P38036 | O80536 | Q0JD07 | | P34683 | | P60471 |
| Q9VG73 | | A6NNA5 | | P67661 | | Q10213 | Q26601 | P41936 | | P22262 | | P14327 |
| P79937 | | Q87K77 | | Q9LES3 | | P08638 | A3BH91 | Q1KKV1 | | Q7UW59 | | O31480 |
| Q9NY43 | | Q9WUJ8 | | A2ZMR9 | | Q5AHJ5 | Q8IZ20 | Q38617 | | P03034 | | P87393 |
| B4F6I3 | | Q4V8J7 | | P37970 | | Q9Y5Q9 | O51653 | P90971 | | O35750 | | P05476 |
| O75367 | | P0A2T2 | | P24417 | | Q9VF30 | Q63369 | Q48514 | | Q9GPH3 | | A0QTT1 |
| P50220 | | P9WMD3 | | P0ACS8 | | Q06639 | Q9UTA1 | P46946 | | P22697 | | O22901 |
| Q62481 | | Q22910 | | P32326 | | Q06163 | P0CG31 | A8XA80 | | Q9LYD3 | | P23939 |
| B0S909 | | Q9M1K0 | | Q8LBQ7 | | P11536 | Q13950 | Q7JQ07 | | Q9LU18 | | P0A6X3 |
| Q9SPG2 | | Q9WX14 | | Q3BJS0 | | P38781 | A7EAG1 | D4ZX35 | | Q38851 | | P42505 |
| Q805B4 | | P71039 | | Q9M9X4 | | P52959 | Q9FXD6 | P73123 | | P22177 | | Q9Y1T5 |
| Q9MAN1 | | Q5EG55 | | P31617 | | P04386 | Q17RP2 | Q58030 | | Q2RNI6 | | Q57475 |
| Q4JB80 | | H8F0N6 | | P15795 | | P05085 | Q14119 | Q8JIT7 | | Q9AE24 | | O86228 |
| P25748 | | P03565 | | Q9RZE7 | | Q24206 | Q8ST83 | P41935 | | P55407 | | Q057E0 |
| Q6DRD3 | | Q06553 | | Q61937 | | Q6UDH3 | Q8S8P5 | O34829 | | P32425 | | O32222 |
| Q91574 | | P9WMI3 | | Q84RD0 | | Q9V9A8 | O23115 | Q4V7W5 | | Q45591 | | Q9PUE0 |
| P0ACP3 | | P0ACK6 | | Q40478 | | P47043 | P29775 | Q6CHI1 | | Q9LZR0 | | P27204 |
| P15314 | | Q6X7J9 | | O95983 | | P23497 | B0ZSF2 | P15419 | | A3FEM2 | | P55109 |
| A6USD6 | | Q9HU78 | | P9WGH3 | | P34021 | Q9FT81 | Q07961 | | Q09390 | | P22625 |
| P0A2Q4 | | P12746 | | Q33DK0 | | Q9USU3 | Q8BDG3 | Q0JXE7 | | Q10160 | | Q8GWU1 |
| Q9IG45 | | P57766 | | P34470 | | Q9V5M3 | O93870 | O17683 | | P18680 | | C6BQP8 |
| Q96EK4 | | Q8N5J4 | | P0C7M4 | | P40965 | P13658 | P07621 | | O31459 | | Q9XG57 |
| O26979 | | P9WME6 | | A2XZR3 | | Q95YE9 | O13606 | Q52221 | | Q5BUB4 | | P39212 |
| Q8XBK9 | | Q973F5 | | A8F7B4 | | P36617 | Q54S29 | Q653Z5 | | Q84W66 | | Q01709 |
| Q9LXE1 | | Q9HJQ0 | | Q9W769 | | Q54YU6 | Q6CQ07 | P59924 | | Q9ZV39 | | C0H3S9 |
| Q93CE9 | | O95475 | | O14627 | | Q8JIS6 | Q9ZNX9 | P35879 | | P70955 | | O34464 |
| A8MWA4 | | P03206 | | Q5W659 | | Q9W2Q1 | P29747 | P18809 | | P52392 | | Q23794 |
| Q05502 | | Q9CAP4 | | O08686 | | Q0VKZ4 | P09176 | P52661 | | P26356 | | O31178 |
| P41900 | | Q9LDE4 | | P13121 | | Q9VH20 | Q9VYG2 | P18811 | | Q22813 | | P04132 |
| P44487 | | Q9LND1 | | Q3TDE8 | | Q94819 | Q03372 | O17573 | | Q7LXL5 | | Q8G5K1 |
| A1YGA4 | | Q2NE55 | | Q9M1V5 | | Q96DT7 | O02279 | P37245 | | P42239 | | P77295 |
| P0ACM1 | | P67738 | | Q680D9 | | P40114 | Q2UMB1 | Q96WN6 | | P52133 | | Q39LF7 |
| Q99811 | | P9WMG6 | | P37671 | | P15619 | A3B6V0 | B1L400 | | O74859 | | Q26603 |
| Q9FMV5 | | Q07783 | | O43543 | | Q8NHY6 | Q55GG6 | Q8LFR2 | | Q9FJ90 | | Q05244 |
| Q0II87 | | P9WMG4 | | O80805 | | Q13342 | O56860 | Q9Y603 | | Q18336 | | P22876 |
| Q874J6 | | Q0P5B4 | | P46604 | | P22856 | Q9XUK7 | Q8U4G4 | | P40962 | | P44030 |
| P50553 | | Q99581 | | B1ZWL2 | | O59780 | P40992 | Q9MA75 | | P19601 | | Q26430 |
| P79769 | | A2RKH4 | | O83046 | | P34447 | Q8N680 | P13892 | | Q57949 | | P40268 |
| Q9PVY0 | | P22537 | | Q8R1H8 | | P08657 | Q84ZT0 | Q8L3W1 | | P60008 | | Q7SI91 |
| Q9LW49 | | P62181 | | Q652I1 | | O74365 | P23890 | B6IPE2 | | P14057 | | O50461 |
| Q4A9R7 | | Q10QS9 | | A5F384 | | Q54GS8 | F4JN35 | O42648 | | Q03125 | | P36322 |
| P21968 | | P0ACM9 | | Q5AI97 | | F4I2H2 | Q9RMZ4 | O87590 | | B3EWG9 | | Q64029 |
| P0AF30 | | P33909 | | Q02441 | | P45447 | Q8W475 | P27871 | | Q9LDT3 | | Q74NA9 |
| P13606 | | P9WMC8 | | Q0JQF7 | | P41813 | P10754 | P20024 | | Q9LSN7 | | Q47156 |
| Q9VJQ5 | | Q8N7R0 | | P31008 | | Q9LGU7 | Q96K62 | P38629 | | Q84ZA1 | | P83946 |
| P53996 | | Q8L4B2 | | A2RU54 | | Q92750 | P20385 | P32085 | | P31475 | | O80294 |
| A3PHR6 | | Q0VD35 | | Q9H2A3 | | P38630 | P14734 | Q05954 | | P31460 | | Q28601 |
| Q8N100 | | Q9LVR0 | | O00287 | | P79051 | P43574 | Q6NW59 | | Q6FJX6 | | O07434 |
| Q8U0P3 | | Q79VI7 | | P70284 | | Q9IZT2 | Q8TBC5 | P45968 | | P13870 | | P55924 |
| A0RCV1 | | B2S3D5 | | Q6K3B1 | | O18381 | Q86AF3 | P04521 | | Q6NVH7 | | P24341 |
| P62087 | | P9WME4 | | A8XSQ8 | | O94278 | P30373 | Q6VBA4 | | Q9HG12 | | P16117 |
| P0A183 | | Q1XDE4 | | P11115 | | Q750N3 | Q99160 | P50844 | | P32086 | | A0RYF8 |
| A6QPY8 | | Q8BHL6 | | Q6K3R9 | | Q10280 | O18400 | Q0JYN7 | | P42427 | | Q57682 |
| Q29087 | | P96856 | | Q9Z205 | | Q54NA6 | Q02930 | Q9DGW5 | | Q40170 | | O22059 |
| P0ACH5 | | Q91ZY6 | | O80337 | | Q5UQB5 | P32027 | P72171 | | P0CJ65 | | P0C0Q9 |
| Q4R947 | | P0ACS9 | | Q9BRJ9 | | Q9BYN7 | O95718 | P37248 | | P34030 | | P24508 |
| P10026 | | C0QKP5 | | O22798 | | Q59RL7 | P52167 | Q8C9A2 | | Q8SW18 | | Q9T1U6 |
| Q5BJU6 | | P9WMD7 | | Q13IY4 | | P43610 | Q9UT46 | O45449 | | P54794 | | P17638 |
| P30339 | | P66812 | | Q6S3I3 | | Q9FHJ6 | P13991 | Q55566 | | P15940 | | P20222 |
| P06689 | | P52932 | | O49741 | | P41410 | O14246 | Q01664 | | Q7AKG9 | | P02280 |
| A1QQ42 | | Q63630 | | Q16594 | | Q8IWY8 | P20153 | Q9LT23 | | O87940 | | P14150 |
| Q7A3K1 | | P0CY09 | | Q5NVM3 | | Q59V88 | P77698 | P37947 | | Q6P0X2 | | P09078 |
| Q9KPS0 | | Q2T9V8 | | Q5JD67 | | Q9SHG6 | A7SBN6 | P27343 | | O82286 | | Q9PSJ0 |
| Q7TWJ2 | | P0C745 | | O35885 | | O51768 | Q62724 | Q9LG02 | | Q8L9K1 | | P15281 |
| Q7XLX6 | | Q9BWT6 | | P35889 | | Q54LB3 | Q07608 | Q56201 | | Q56XP9 | | P16654 |
| A3MUI7 | | P9WMD0 | | Q9LY29 | | P08155 | A9CH39 | Q9FIW5 | | Q8GAL4 | | P24716 |
| P17305 | | Q9M836 | | P76268 | | Q24472 | B6V2N4 | Q9C6P5 | | Q3EAS6 | | P18863 |
| Q61329 | | Q9XI33 | | Q9M0R0 | | Q60563 | Q17381 | Q8RYD1 | | Q9XIB5 | | Q04621 |
| P34881 | | Q9QYC3 | | P06574 | | Q9VL72 | Q6XDT6 | O22900 | | Q7NAN2 | | P03041 |
| P53564 | | Q755R9 | | Q8GWK7 | | Q54GG9 | Q6FSW2 | P24242 | | P39885 | | Q8G5J9 |
| P26019 | | B1AJ90 | | P9WGI3 | | Q59MD2 | P18516 | P46665 | | P55701 | | Q9ZCH6 |
| P28925 | | P9WMC6 | | P0A3J6 | | Q12244 | P32566 | P21867 | | P96683 | | Q47149 |
| G3ECR1 | | O73692 | | Q9LQ08 | | Q23405 | P40041 | Q08812 | | Q5UR40 | | C0H3Q8 |
| P54098 | | Q9QZS2 | | Q9R9U0 | | Q6I576 | Q27403 | Q7T6X9 | | P39404 | | P24274 |
| A7EFH4 | | P0ACU8 | | P46897 | | Q8RWY4 | P27705 | Q8GY61 | | Q94JI5 | | Q99284 |
| Q2VIQ3 | | Q5UPI1 | | Q8LEG1 | | Q9I1X7 | Q9P6H9 | Q9C7W2 | | Q9V6Q2 | | P07695 |
| Q00942 | | P9WMC4 | | P0ACL7 | | P03010 | O14362 | Q7WTB0 | | O32192 | | B7EIH2 |
| Q14865 | | P0A0N5 | | Q84TE9 | | Q9LSP8 | P30020 | O05103 | | P15315 | | P02345 |
| Q3UTQ7 | | P9WMC2 | | A2YGL9 | | Q60GC1 | Q6FJK6 | Q9XIN7 | | Q39LF8 | | P31261 |
| Q4UMJ0 | | Q5TGS1 | | Q5VPE5 | | Q550C8 | Q92985 | B1Q2A8 | | Q9LTJ1 | | P13772 |
| O14746 | | P9WGH0 | | P44978 | | O60035 | Q9Y7D3 | Q94131 | | Q42464 | | Q3E835 |
| Q59Y41 | | Q8YQL3 | | P0CAV4 | | Q5JET4 | O02334 | Q6NI56 | | P75974 | | O32242 |
| Q87QP4 | | Q8UW72 | | P31616 | | P39001 | O14470 | P75604 | | P60622 | | P13320 |
| P30318 | | O68887 | | P0ACL6 | | Q9WTY8 | Q46901 | Q28F43 | | Q9LXD8 | | O74896 |
| Q8TUS2 | | P52617 | | P15360 | | Q59LV8 | P07247 | P24488 | | Q27896 | | P46496 |
| P38086 | | H8EXN1 | | P33234 | | Q54Y73 | Q96680 | Q9S851 | | Q5UP03 | | P22562 |
| Q9HAP2 | | P0ACS0 | | O80674 | | Q9UT23 | O24629 | P08345 | | O05494 | | Q58721 |
| E1BE02 | | Q8IXM2 | | Q39088 | | P10961 | O45666 | Q9FGD2 | | A4D998 | | P09590 |
| Q99708 | | Q38740 | | Q6REU5 | | P25611 | P33288 | Q9JMQ1 | | Q5A1E3 | | P43684 |
| Q0VBD2 | | P28830 | | O07776 | | Q54G94 | Q870J1 | Q9DE09 | | Q10241 | | P12552 |
| O13412 | | P70120 | | P05048 | | Q55681 | P33181 | Q8GXL3 | | O26310 | | Q28598 |
| O96651 | | P62088 | | A8MTQ0 | | P40377 | P17867 | P06843 | | P17486 | | P09020 |
| Q58DC8 | | Q8U2H1 | | Q8QME6 | | Q10332 | Q6UDI9 | P03688 | | Q59PR9 | | O33348 |
| Q94JM3 | | P9WME3 | | Q9FMX2 | | P39143 | Q9UL49 | Q53VV5 | | P38304 | | P13275 |
| O05560 | | B4RFS9 | | Q9ZR14 | | P46954 | C4Y732 | Q9LUA2 | | P29904 | | P21681 |
| Q5F3F2 | | P9WMG2 | | O49550 | | Q8T2T7 | Q91392 | Q54S00 | | Q9HDS5 | | Q7AKN0 |
| P79149 | | A6H1A3 | | Q9XH35 | | P11938 | Q5AJU7 | P22816 | | Q37906 | | Q47150 |
| Q5RA25 | | A5PJU7 | | Q8ZTY0 | | O60130 | O67465 | Q23489 | | O31432 | | P15859 |
| Q08CM4 | | Q9V2M0 | | Q9C7C3 | | Q9W349 | Q6H6Q7 | P43462 | | P39601 | | Q90683 |
| P81133 | | Q9UY16 | | Q6K5X1 | | Q63003 | P31369 | P77615 | | Q9LP07 | | P13975 |
| C5E4K0 | | Q8TZE2 | | A0RXH7 | | P19822 | Q6ZNH5 | Q8RYD6 | | Q9SI19 | | P20270 |
| Q75HY5 | | Q5XEA3 | | Q0JB92 | | P10563 | P82798 | Q2FL78 | | Q8TBB0 | | P83183 |
| Q8K4B0 | | F9VND5 | | Q91848 | | P32591 | P18488 | A2BKJ8 | | Q9STJ6 | | P14839 |
| Q9JK30 | | P0A2T4 | | Q2M1V0 | | P10078 | Q8GVZ8 | Q8TVS6 | | P29588 | | Q8JN69 |
| Q9FVS2 | | P68922 | | P34348 | | P52025 | P50729 | O84905 | | Q655V4 | | A0QRY5 |
| Q9ZD95 | | P9WME8 | | Q86XF7 | | P73625 | P15533 | Q9ZB11 | | P76114 | | O31834 |
| Q80SX8 | | P67702 | | P9WMH4 | | P21228 | P08557 | P69781 | | P09007 | | P14307 |
| P24486 | | Q8Y0Y3 | | A2YN17 | | C1A4A9 | Q22811 | Q5Z6A4 | | P29485 | | P08365 |
| Q1RI08 | | Q8IS98 | | Q51786 | | Q381V6 | Q7T105 | Q9LVW2 | | P97516 | | Q64204 |
| Q9FKL6 | | Q86QI0 | | P0ACK8 | | O59746 | Q0JP99 | Q68EY3 | | Q04310 | | P22995 |
| P01103 | | P31347 | | P10585 | | Q73MQ8 | Q9UDV6 | P56670 | | P37338 | | P03049 |
| P23913 | | Q38504 | | Q6NCZ5 | | Q59QW5 | P09081 | Q4P4Y2 | | Q70II3 | | Q3ECU8 |
| Q9CL21 | | P0AAR1 | | A6NFD8 | | Q94407 | C0SVV6 | Q23622 | | P38082 | | P14838 |
| Q6GNP2 | | E0TY12 | | Q9SEZ1 | | P11638 | P13925 | Q5QNI5 | | P03048 | | P10427 |
| Q12WQ0 | | P9WMI9 | | Q9FKG1 | | Q5JMF3 | P09087 | O80928 | | Q9P7V3 | | P75970 |
| B3LQ10 | | Q9CQ36 | | Q9FHA7 | | O33367 | Q6AT25 | O07008 | | Q47744 | | P06353 |
| A2SXS5 | | P9WF38 | | P54292 | | O59744 | P49309 | Q9LV52 | | Q3J6K8 | | Q54YI1 |
| Q9NPC7 | | P52144 | | Q58940 | | Q05958 | O81782 | O97764 | | Q3E703 | | P81192 |
| Q1RKH8 | | P0C5Z0 | | P0ADD8 | | P12894 | C4YHS3 | Q9M8K2 | | Q6QHK4 | | P22404 |
| Q5BKZ1 | | P9WMH6 | | Q9XFB1 | | Q9VH70 | D0VYS2 | P38749 | | B2JU32 | | Q3E829 |
| P19532 | | Q84RD1 | | Q01980 | | P42282 | O45436 | Q10424 | | P29284 | | P11020 |
| Q96ME7 | | P18712 | | A2XDK5 | | P57381 | P03108 | Q9M9B2 | | O82268 | | O31865 |
| Q5ZM33 | | Q7RTV0 | | O15522 | | Q383A1 | Q9N0N3 | Q53NF7 | | O07007 | | P15857 |
| Q1HE26 | | P67674 | | A2XLF4 | | P22035 | Q90643 | P70061 | | P53102 | | P76611 |
| P0A603 | | Q9LXK1 | | Q8C0Y1 | | P05445 | Q6BLM3 | O07567 | | Q8EWC5 | | P51705 |
| Q9ZIB7 | | P64387 | | Q8GZA8 | | Q09772 | Q93705 | Q6S4P4 | | P32761 | | O29774 |
| Q8R316 | | Q705F5 | | Q1XDE5 | | P48743 | Q8N720 | P34257 | | P93007 | | Q5F881 |
| O14901 | | P23397 | | Q06615 | | Q7ZVR6 | Q8MQH7 | Q9YDZ1 | | P53187 | | P03672 |
| Q6YRL3 | | P0A9V5 | | O30919 | | P21538 | O74804 | Q2QW44 | | P0CF60 | | Q8EIX4 |
| O43102 | | Q47948 | | Q7RTU4 | | Q8L7S3 | P29774 | A0MES8 | | Q9Z2J9 | | P09637 |
| P70323 | | B2I4R3 | | Q9FMR3 | | B6MUN4 | Q54WV0 | Q9SAD4 | | Q9W6I9 | | P14309 |
| O14770 | | Q971T6 | | Q9FH37 | | Q05192 | Q2QMT6 | Q9RXY7 | | Q39127 | | Q4UNB3 |
| P49698 | | Q83KJ8 | | P9WMD9 | | Q96KG9 | P39970 | O24463 | | Q93WY4 | | O07779 |
| P53338 | | Q5EE01 | | O56129 | | Q9XI07 | Q7GCL7 | Q5JUK2 | | Q08702 | | O22002 |
| Q9Z8M9 | | D3GKW6 | | O60248 | | P08770 | Q9FY82 | O13472 | | P51985 | | P27079 |
| B3DM25 | | Q01797 | | P03696 | | Q09555 | A2T928 | P36145 | | Q67591 | | Q54428 |
| Q9M7Q3 | | P9WFC3 | | Q9H2W2 | | Q27368 | Q93Z00 | P39356 | | P13342 | | P55409 |
| Q5ZJF3 | | Q92HL4 | | Q53N90 | | A7HAB8 | Q9ZSI7 | Q26657 | | B0W1V2 | | P23822 |
| P13474 | | P64131 | | Q9RC52 | | Q9UUA2 | Q9HLK5 | Q8VZ02 | | Q9C591 | | O32235 |
| P48000 | | Q4QNL8 | | Q1CAP8 | | Q5A287 | Q62396 | Q56XR0 | | D5W6H7 | | P0DJH6 |
| Q08CW8 | | Q46995 | | Q0JKX1 | | Q54WU0 | C4Y4V1 | A1RZT8 | | P47237 | | P06965 |
| P81413 | | P68926 | | Q07597 | | Q8KBK0 | P31396 | P20009 | | Q9HB31 | | P18865 |
| Q3SYB3 | | Q9P8G9 | | Q9H0W7 | | P53243 | O46339 | P06846 | | Q6Z461 | | P18679 |
| P03123 | | P24649 | | Q9D8P7 | | O94065 | Q95RW8 | P72065 | | A7UFC2 | | Q7M2P1 |
| Q5ZLN5 | | P41371 | | Q6NT89 | | P36341 | P10862 | Q54X22 | | Q83BT6 | | P33785 |
| Q4R6Y6 | | Q8NEZ4 | | Q623D4 | | P09959 | Q9ATB4 | Q25514 | | O05086 | | Q05459 |
| P50456 | | Q7TMY8 | | Q8NDY6 | | Q54F46 | Q9SI37 | Q95PX3 | | Q7XAQ6 | | Q46558 |
| Q6L4N4 | | Q8WN22 | | Q52QU1 | | O00841 | Q9SYL8 | Q8N0Y2 | | Q9SIT9 | | Q9ZX29 |
| Q14AI0 | | Q03164 | | Q9BYE0 | | A7TGQ2 | Q3U0L2 | P33651 | | P20434 | | P55565 |
| Q99551 | | Q24742 | | Q9N1Q6 | | C5DKJ7 | P09775 | P37247 | | P43344 | | P45903 |
| Q67U94 | | Q96T58 | | A2C1Y5 | | P20824 | Q19849 | O23659 | | Q42498 | | P22975 |
| P40427 | | O60673 | | P18750 | | Q8S9G8 | P91623 | P55629 | | P14204 | | P06020 |
| Q90YI8 | | Q8IWI9 | | P02964 | | Q54WZ0 | Q9W3M9 | O06703 | | Q88469 | | Q28466 |
| Q9SD40 | | Q3L8U1 | | A9B9X1 | | O83964 | P38165 | P37344 | | A8R3S7 | | P15856 |
| O76971 | | O60281 | | P0ACU0 | | A9ZPC9 | P18412 | Q0E7J7 | | Q1M7A0 | | P15858 |
| Q14549 | | P15822 | | A2YQK9 | | O34481 | Q9SGQ0 | Q09376 | | B2A5L5 | | P11470 |
| Q67UE2 | | Q9UMN6 | | P47347 | | P30322 | Q9FNY0 | Q9C9L2 | | P40685 | | P15860 |
| O80416 | | Q9Y618 | | A8R3T0 | | P20945 | P15062 | Q9I1P2 | | Q9WYK0 | | P23681 |
| Q9PT68 | | B0R0I6 | | Q9C8Z9 | | Q38CE9 | O96785 | Q6X7J5 | | Q1LMQ4 | | P43640 |
| Q3L6L8 | | Q9QX47 | | P0ACT3 | | P48785 | Q6E434 | Q8TWM7 | | Q92522 | | P29840 |
| O02755 | | Q8WYP5 | | O75446 | | P39956 | Q9LG05 | Q9CA27 | | Q9R9T9 | | P25735 |
| Q5ZKF4 | | P70670 | | P83733 | | Q54LY5 | Q9BSK1 | O87389 | | P96686 | | P20224 |
| Q95RV2 | | Q6XKE6 | | P22996 | | Q9DBY0 | P40466 | A4FFJ9 | | P51502 | | O34766 |
| Q8H1E4 | | Q8NFU7 | | P0A9F0 | | Q75BN0 | D5AKX9 | Q10116 | | O34994 | | P06903 |
| Q8QZV0 | | Q8IYD8 | | P9WMF9 | | P39961 | Q754J3 | O13493 | | Q94AD1 | | Q37907 |
| A6H7A8 | | Q6N021 | | P52106 | | Q7M3M8 | Q5URB4 | Q84TE6 | | Q9LYU3 | | P14816 |
| Q7RTS3 | | Q9EPQ8 | | P69343 | | O13360 | C4R1K8 | Q9C8W9 | | Q88FX7 | | O32067 |
| P26651 | | Q14966 | | P9WGM4 | | F4JP48 | P18416 | O83295 | | Q7XLP4 | | Q89707 |
| Q13516 | | Q12888 | | Q9Y4Z2 | | O67837 | Q8VWV6 | D4ZX34 | | P19219 | | P11866 |
| A0R3S7 | | Q9UIF9 | | P68589 | | O27438 | O94392 | P94387 | | Q38841 | | P71388 |
| Q5JF28 | | P47024 | | Q6QPM2 | | P21969 | Q6ARL8 | P37682 | | Q9M9L6 | | P31262 |
| Q99453 | | P17546 | | Q9LM84 | | Q6H8R9 | A9CJC9 | Q9M2U1 | | Q94126 | | P03036 |
| P16472 | | P0CO17 | | Q9SUA9 | | P40971 | Q04449 | A8HV31 | | Q8H0T5 | | Q9PUA8 |
| P07642 | | Q9CWL2 | | P0A2S7 | | P31244 | P35203 | Q9LDE1 | | P13295 | | P33997 |
| O42837 | | P14565 | | Q46791 | | P93119 | O13862 | Q8GY42 | | Q9M644 | | P81193 |
| O75840 | | P97789 | | Q84R27 | | Q96MR9 | P73421 | Q8PXY1 | | P22915 | | Q64201 |
| Q32LN0 | | Q13029 | | Q9RX92 | | Q8K1M4 | P28364 | P35883 | | Q00453 | | P37371 |
| B4UFZ1 | | Q8BG87 | | Q9LY05 | | Q9ZTX9 | Q9NR11 | P52906 | | P56644 | | O34791 |
| P28518 | | Q5TGY3 | | P75243 | | Q554P0 | Q756C3 | P36219 | | P24536 | | P31504 |
| Q99598 | | Q9Y6X0 | | O15499 | | Q59NX5 | P31778 | P19655 | | Q9FHA2 | | P12059 |
| Q1XID0 | | P51531 | | Q94AR6 | | O51272 | Q09528 | P23050 | | P75952 | | O31417 |
| P37551 | | A5E0W5 | | Q41188 | | Q7KQZ4 | A7TQK0 | Q9SV30 | | Q9HXE2 | | P82897 |
| Q8NG50 | | Q6ZPV2 | | A2ZH47 | | Q940Y3 | P27610 | P90953 | | Q46TH1 | | P09635 |
| Q148M8 | | Q99315 | | P9WMD5 | | O32044 | Q46731 | Q3SWU3 | | Q18053 | | P13544 |
| Q9BRP0 | | O48653 | | A5E7M8 | | P07261 | P29776 | P51593 | | O32506 | | P13967 |
| Q9EYV5 | | Q27152 | | O07019 | | P53438 | O83149 | Q941I2 | | Q9UW23 | | P03040 |
| P49716 | | P19560 | | P67430 | | Q5AP80 | P04530 | Q9LQQ2 | | O73592 | | P11189 |
| P58497 | | P32657 | | Q96NC0 | | F4JSG3 | Q8WUU4 | O80302 | | Q870I4 | | P05998 |
| P32144 | | Q9Y4B4 | | Q6XYB7 | | P40657 | Q9U2R6 | P32030 | | P39897 | | P39901 |
| P19940 | | Q9V2F4 | | P9WMB6 | | P21189 | P48731 | O35003 | | P54988 | | O31490 |
| P53769 | | Q7Z591 | | Q8VY64 | | O14130 | O33149 | Q9D4V4 | | P06838 | | P42554 |
| Q2YJ50 | | P13055 | | Q8N8Y5 | | P31264 | A8MQY1 | Q20257 | | Q09771 | | P30791 |
| Q63520 | | Q7Z3K3 | | Q96PT4 | | Q09922 | Q05309 | Q9FJK3 | | O56862 | | Q99282 |
| Q9K492 | | P39806 | | B9MRB2 | | O51125 | P79742 | F4IN23 | | Q8G2M8 | | P15482 |
| Q9ZTC3 | | B8FXD7 | | H8F2P4 | | Q9P7W8 | Q76IQ7 | Q9C7P4 | | Q9SX97 | | P09964 |
| Q5K4R0 | | A5VHK2 | | Q9S7L5 | | P62597 | Q8KRZ2 | P53772 | | G5ECT8 | | P55813 |
| P62177 | | P46974 | | Q9ZVA0 | | Q3ABU1 | P07908 | P23801 | | Q9SIC7 | | P72191 |
| O86382 | | Q8BVE8 | | P0AF10 | | P10277 | Q9I9E1 | O31690 | | Q57824 | | P20710 |
| Q9ERU3 | | P52701 | | Q8VWQ4 | | Q8S8E8 | Q10902 | Q9D0B1 | | Q9LT45 | | P76138 |
| P52688 | | O00874 | | O08335 | | P49880 | Q90270 | A3BYC1 | | P36395 | | P41726 |
| B8AH02 | | P90493 | | Q9ZQP3 | | Q9S7G7 | P40379 | P40154 | | Q60328 | | P41018 |
| Q2YJF8 | | A2CG63 | | A3LZM4 | | P36598 | P56158 | Q94CK9 | | O78417 | | P25921 |
| A3PCR4 | | P09310 | | Q874N0 | | O32215 | Q24705 | P74892 | | Q9W6D8 | | A8A8H1 |
| P14506 | | Q8K083 | | P9WME0 | | P50534 | P34437 | Q9FYJ6 | | Q9RS64 | | P84408 |
| Q46397 | | Q6PG95 | | Q99929 | | Q5XG87 | Q3ED78 | P04526 | | O81900 | | Q51773 |
| Q44273 | | Q9HAZ2 | | P37978 | | O32071 | Q23045 | G5ECU7 | | Q6FNR0 | | Q45100 |
| Q6TDU1 | | Q29L50 | | B8AIK3 | | E5AV36 | P25932 | Q82PM3 | | Q29480 | | P03050 |
| Q5E9A1 | | Q92610 | | P42105 | | P21192 | Q5N8Q4 | O17748 | | Q7USB0 | | P59890 |
| Q04RG9 | | P51827 | | P70368 | | Q9HM08 | Q8GAH9 | Q54YG9 | | A1STA5 | | Q62792 |
| P26487 | | P0CO19 | | Q9RY80 | | P40418 | Q55BM5 | Q84BZ4 | | P64588 | | P20044 |
| P68951 | | Q4WVM1 | | A6ZXD3 | | Q9SVF1 | Q50D79 | B5YJS2 | | O34970 | | Q02513 |
| P0AGA7 | | Q9P243 | | Q65DU9 | | O94490 | P39389 | Q10R18 | | P52743 | | P08433 |
| A6UEL7 | | P08546 | | Q2SS60 | |  |  |  | |  | |  |
| 7131 non-binding proteins | | | | | | | | | | | | |
| Q9X4M1 | Q6CHD6 | | B4EWL8 | | B1XEH8 | | Q96LM5 | P0A7K4 | P37653 | | Q636G2 | |
| Q5L8C0 | Q9XE98 | | A1WF54 | | Q6G770 | | Q6HDN3 | B9DUV7 | Q9XT58 | | Q6ZPR4 | |
| Q6G461 | A2BYL0 | | Q9ERS1 | | Q2JSD0 | | Q8R7U6 | P33201 | P0CF57 | | Q9XFX4 | |
| Q12233 | B0WEV5 | | A5F7H0 | | Q1R0I1 | | Q9Y239 | B1H1P9 | P29067 | | O05509 | |
| Q14692 | Q5BF62 | | Q4KHG0 | | P62202 | | Q98QU6 | A6LEP5 | Q9JIB3 | | Q10220 | |
| P59051 | P62156 | | A9KN62 | | Q3IDE8 | | P57566 | Q82FG6 | Q6NTV6 | | Q4R4K5 | |
| Q8WUQ7 | Q88FY0 | | B9JGK2 | | B9KHN3 | | Q9CR08 | C4K0Y4 | C3NG95 | | O52344 | |
| A8K010 | P40546 | | Q313Q3 | | Q2MY45 | | B5FQL3 | B6JN96 | A2SH96 | | Q7V5D4 | |
| A5CU64 | A7IA28 | | Q6CJ50 | | P68056 | | B8DAT9 | A8V0B3 | Q2NFV7 | | Q3K3R1 | |
| A4SXD7 | Q03EC8 | | Q6P575 | | P84312 | | B4TFD8 | A4T072 | P48223 | | Q16AP3 | |
| Q6P5D4 | Q12ZV1 | | A5FKC6 | | B2UT00 | | P0A5H9 | Q7TQ72 | P01944 | | Q06GT7 | |
| P33070 | A8IJI4 | | Q0P3K6 | | Q5WT85 | | Q9QUR7 | Q6SLL5 | P29348 | | Q8BZI0 | |
| A9L3M8 | Q76631 | | Q980I9 | | Q6IE75 | | A5D7H5 | A4WTU0 | P30632 | | P9WPG9 | |
| B5BGT9 | Q9CLR1 | | Q9RWM4 | | Q9Z9C2 | | B7HDY7 | Q5B0V6 | O10371 | | A7IDJ5 | |
| O10683 | Q5XAZ0 | | Q9K1G6 | | P01903 | | Q80ZF0 | Q1I637 | D4B385 | | B7LGU9 | |
| Q5HR81 | Q1BG58 | | P25635 | | P21082 | | B4R1D8 | Q8WMR3 | P12264 | | Q5RCP7 | |
| Q3SEU9 | Q9UHW5 | | A6VMA4 | | C0MA57 | | Q2FDW8 | Q0P8U3 | P33728 | | Q7V9C3 | |
| A3MTA0 | Q9NUL7 | | A0K2D5 | | A9VZF3 | | Q92J43 | A5FFY3 | Q0TAL1 | | Q6CXX3 | |
| Q83MR9 | A4H4W7 | | Q9HJT5 | | P56365 | | Q5ZIF8 | B0CIQ5 | Q18086 | | P41169 | |
| Q5HM41 | P69355 | | Q15X58 | | Q7VLT3 | | Q32KH5 | B2T5I2 | B2G851 | | Q8JZS9 | |
| Q498J9 | A6GZ81 | | Q21CK7 | | A7Z7C6 | | Q5R516 | Q0K6G3 | P0C8G7 | | Q4R6Y8 | |
| Q14680 | B2K5V7 | | O82481 | | Q03066 | | A0LCZ5 | C1KZK6 | P41635 | | Q9JW69 | |
| A9R4I4 | Q0TAF4 | | A6URB0 | | A9VWV4 | | Q3JR29 | P96188 | B3EMN7 | | Q57KH0 | |
| Q6AYP2 | C3KBZ2 | | Q9H773 | | P20336 | | A6U2E7 | Q487Z1 | Q9WUM3 | | B2SEY2 | |
| B5VM60 | A5IT53 | | Q1WP01 | | Q9MSP1 | | Q6PHG8 | Q1GZ01 | A0L7X0 | | Q84S07 | |
| Q8XVI7 | Q12724 | | A4IY05 | | Q57KE9 | | Q42347 | B1HWC9 | Q5RAH8 | | Q31XD9 | |
| Q1WT38 | G1JSL4 | | Q9R0H5 | | C4K7A6 | | A3NAN4 | Q5HGS8 | Q28UR1 | | Q03UE1 | |
| P0CQ98 | Q0TSS9 | | P46268 | | Q7TPS5 | | P49279 | A9WGQ8 | Q88VG6 | | O04922 | |
| Q8DU64 | Q31D45 | | Q1CV46 | | O68970 | | Q6F0J2 | P69912 | A4YBY0 | | Q7YQK8 | |
| A1KSU7 | Q1MPP0 | | Q2NVM9 | | Q4A5D3 | | B4UHX9 | O82515 | Q28TJ7 | | Q9PPT9 | |
| A1E9M8 | P09088 | | P28745 | | P59638 | | Q6FU45 | Q164N5 | Q28PL3 | | Q5XAL6 | |
| A8F0P3 | Q0A2C9 | | B7LYF5 | | Q39BU7 | | Q5FJX0 | Q9JLB9 | Q3UAW9 | | Q8NC38 | |
| Q3K723 | A5GJE9 | | A4G1T2 | | P03000 | | Q9BAC0 | Q5NVM9 | Q68W93 | | P13256 | |
| B0C1E3 | Q9FT51 | | O49814 | | Q3E7L7 | | Q0S277 | A5CXH8 | A4STP8 | | B0KA53 | |
| B1X6E6 | P04778 | | Q7VP92 | | Q03I57 | | A7FLE4 | Q6ZTU2 | B1W3Y1 | | Q5M0W5 | |
| B1JAI6 | A1A3W4 | | B0JXE2 | | P28002 | | Q8P317 | P60430 | P36891 | | P9WNV8 | |
| Q2IJB1 | P0C1W4 | | P59496 | | A9W8M8 | | B4U743 | A1TM37 | A2AWH2 | | Q1JAS5 | |
| A9WQB1 | O19006 | | Q54B49 | | Q89YW6 | | B8DRD1 | A1W574 | B4HPU1 | | B5ZB67 | |
| Q5HF94 | A0KNE5 | | Q8JZW8 | | A7HCI1 | | Q1QDH4 | A1WLL2 | B3EUR6 | | B6ELD3 | |
| Q318I6 | Q5CD95 | | Q8C010 | | Q8P4H2 | | B5EWY2 | A1V8B6 | A2BY12 | | B6IZI4 | |
| B2S2E7 | Q8BG21 | | Q4QIS1 | | Q110B9 | | B4SLH6 | B3Q039 | P43367 | | P30589 | |
| Q72BR0 | A0QSD3 | | P36647 | | O35732 | | B3W8L5 | Q2EEW9 | Q211D9 | | Q04C52 | |
| Q3AS97 | Q6B8P2 | | A4FPT8 | | Q9D7V2 | | Q3IFY0 | P20136 | Q30P97 | | Q0G9N0 | |
| P57188 | P92548 | | A9R906 | | P01962 | | B7IF30 | B2AGT3 | Q0AZ36 | | Q3K1V6 | |
| Q8DD20 | P0C447 | | A5E0U1 | | A5E8V7 | | Q98K23 | A6U859 | O70790 | | Q6GR30 | |
| C6A1F8 | P0A461 | | P0C507 | | B0SA37 | | O46528 | B9KZY2 | Q7N3U8 | | Q48VS2 | |
| C3MZG6 | P17196 | | Q3SKN7 | | A9BJD6 | | Q8EUQ1 | P00513 | Q5XAI2 | | Q5RD88 | |
| A4YIM8 | Q2YMM3 | | Q9CBU1 | | Q32C86 | | C3MDH9 | A6MMT5 | B9L7Y6 | | O32436 | |
| B6YSQ7 | Q9VE61 | | B5DZN7 | | Q0BYD9 | | Q9HS16 | Q3ID16 | Q8TVB8 | | Q9BH21 | |
| O66570 | Q05770 | | Q98DD5 | | A1TAR1 | | B2TPB8 | O45436 | B9DIU4 | | Q3UNU4 | |
| A6MMC0 | Q8NWB9 | | P03296 | | B3QWJ5 | | Q7V5X8 | A4JF73 | Q9W4T4 | | P04245 | |
| P17703 | P20348 | | Q68X19 | | P65426 | | Q9XHE7 | Q033M9 | C5A5P5 | | D3VBF7 | |
| Q3ZIZ5 | Q72H18 | | B8DTN1 | | Q608K0 | | Q7W2X8 | A0ALV7 | B5F9U8 | | A9WFP8 | |
| Q13XB9 | A7ZQK5 | | Q9S142 | | A4WTH0 | | A4QLV7 | Q72NG0 | Q5HK23 | | O36396 | |
| B7LSB5 | Q8CRI6 | | Q87008 | | Q6MCB9 | | P62402 | A4QJT5 | Q6WB93 | | B0UTU4 | |
| Q1PFW3 | Q8RVL2 | | P25835 | | Q28UY1 | | P19870 | P48176 | C6BXG0 | | A9VMY8 | |
| A0JPQ9 | B0YBR5 | | P32586 | | P37294 | | B3QSS3 | B1X023 | A2WXD3 | | Q46CG8 | |
| Q9QZE5 | A8QB25 | | P23462 | | Q62L77 | | P67919 | Q82JF0 | D5ARZ0 | | C4XLZ1 | |
| Q4JQX9 | A1ADR4 | | P67883 | | A6VJR0 | | Q8Z064 | A0T0B8 | Q3SLQ6 | | B8IZU2 | |
| F2I5T1 | Q8R3L8 | | Q6FDW7 | | Q6HPN2 | | P58555 | Q6QHC5 | A1S6G7 | | Q24789 | |
| B1X7E2 | Q5HCG4 | | P0AFU9 | | Q5SJ79 | | P33486 | P66339 | Q7MSW1 | | P29823 | |
| Q5VYK3 | Q2GCH5 | | P01777 | | Q3YWV4 | | Q8K2M0 | P0AC64 | Q9SLP1 | | Q8KRD1 | |
| Q91WK2 | Q81WI5 | | A7FPG2 | | Q7W865 | | A5I7J0 | O13715 | A2SAG2 | | P24547 | |
| Q07TY1 | Q9CE35 | | Q3SWX5 | | Q6FZD8 | | B2S100 | P0ADR7 | B0BUP9 | | O66271 | |
| Q3II11 | A5I2S2 | | O75474 | | Q5DU28 | | A8EY33 | P46014 | B4RJC7 | | C3MTW9 | |
| Q5E3U1 | O89079 | | B1IW48 | | B7KCB0 | | Q2YZB2 | P53621 | Q5WFT2 | | Q5R941 | |
| Q9LGE3 | Q6N4U5 | | C3LP79 | | Q8NG27 | | A5GD28 | Q66H47 | A8ZVH5 | | A8Z342 | |
| B7L4W1 | A4VTK3 | | Q1JHV6 | | B2SEV7 | | O00230 | Q3T0T5 | Q6GK52 | | Q57HX5 | |
| B1JUA6 | B8GRD3 | | A2Y0X2 | | Q2N7X0 | | A3D0G8 | A4QLS8 | B5BL63 | | Q039B3 | |
| Q9HUB7 | O27191 | | Q9JTR0 | | Q7U4Y5 | | Q21037 | A3QJR5 | B9LAF7 | | A6MMB2 | |
| B0U6M9 | B5Z8U1 | | A5E969 | | Q7ZX64 | | B5EFI9 | Q01643 | Q9QPN4 | | B7VLE1 | |
| Q6G6W1 | P97802 | | Q6BVY4 | | A7GSG2 | | B7N762 | Q1IWN0 | Q2YTK0 | | Q2FKN2 | |
| Q9QWG5 | Q7U322 | | O67221 | | Q6IRK9 | | A3LT90 | Q7WCW3 | Q5X6S7 | | Q8SR54 | |
| Q3T052 | P61123 | | Q7K2Y9 | | A0M8S7 | | Q4KFC3 | A8A129 | B1YRQ5 | | A9IU40 | |
| Q9Z2T2 | Q9LVZ3 | | Q3EBP1 | | Q8WHY0 | | Q861Y2 | Q9X166 | Q5DYF2 | | P32609 | |
| C6DF66 | Q9UZV7 | | C1AN55 | | Q72CG9 | | C0MAV6 | Q6GEP0 | Q9JV18 | | Q68S18 | |
| P65221 | B0SEH8 | | Q2FW06 | | Q8X5L7 | | Q5XGS8 | A3M804 | A3NPW9 | | C3MAY2 | |
| Q3AWF3 | Q7YT39 | | A2SQ87 | | Q94CG2 | | A3N6P6 | P0C9D8 | P50547 | | O93849 | |
| C3PMK2 | Q73GB4 | | Q04CX7 | | A8AU84 | | Q8XY07 | Q9PE64 | Q479B1 | | Q87VB6 | |
| Q55BR6 | Q88MP5 | | P49491 | | Q28021 | | B7LRT1 | B9DSV0 | Q5ZKK4 | | A7TH93 | |
| Q9LR64 | Q5E9Y0 | | Q4R959 | | P27040 | | Q99V38 | Q822M1 | Q2W2K7 | | P17936 | |
| Q5DRA4 | Q2JV29 | | Q9ZQA1 | | B0VU08 | | A6U4T8 | Q5HNN6 | Q01973 | | P25180 | |
| P65676 | A7Y3K4 | | P34147 | | A6LLM8 | | Q2YBS3 | Q1ME34 | P58687 | | Q4QMA6 | |
| Q0AWT8 | Q0TIC2 | | A6VDH0 | | A6MVY4 | | B7HL47 | B2S3Y4 | A5D508 | | B4NSS9 | |
| B8H164 | Q034X1 | | A0R2X1 | | Q3AWT4 | | Q8MMN1 | Q9UNE2 | Q21CH6 | | A8GAV3 | |
| O67262 | Q6PHI9 | | A3MF00 | | P16466 | | Q9AP35 | O42890 | Q56254 | | Q39IQ4 | |
| P59024 | Q5BDX1 | | B0TTS9 | | Q48V78 | | B1X0Q1 | Q6GEK8 | Q6MBS7 | | Q55798 | |
| P42512 | Q925F3 | | A8F753 | | B5R424 | | Q7NS58 | Q33C10 | Q71ZC5 | | A1T097 | |
| P14528 | A5WE20 | | O15243 | | P35084 | | B2S2D7 | A6LPQ6 | Q71WE5 | | Q2J454 | |
| Q6R4B4 | A1WVW0 | | Q3AAM1 | | Q7YRU7 | | B5RQD7 | Q96AT9 | Q97WW0 | | Q8E5V3 | |
| Q63264 | Q6TH22 | | B3A0Q9 | | Q01KC2 | | A8GB18 | Q7ZA46 | A5UCW8 | | Q13115 | |
| Q4L5W2 | B8EBJ7 | | C0Q479 | | A8GWR2 | | A6X8E9 | Q5QN13 | Q57840 | | P28981 | |
| Q48FN1 | Q1KVV1 | | A8AIK9 | | Q7N6I4 | | Q5R7U4 | A1KIJ9 | B4U6A3 | | B5RHE0 | |
| Q8DTG3 | O60245 | | Q88425 | | Q9PKR7 | | P62537 | Q8XAY7 | B4TXQ7 | | Q66655 | |
| Q9CQ07 | A5IGU1 | | P02174 | | Q2KUS4 | | A7NAI2 | Q9LDE3 | B7N7W3 | | Q3U827 | |
| B2ID03 | B7V485 | | Q85V01 | | O25095 | | Q5N638 | Q7W603 | O77741 | | Q46IT2 | |
| O83128 | Q9RWB4 | | A4SLN8 | | P0AB34 | | A1WZG0 | B7J207 | A1REA8 | | A2S5J7 | |
| A5IRV3 | P70335 | | A5E1N2 | | Q92FW6 | | P00128 | P62285 | Q3ZZM4 | | P0AAF1 | |
| P62878 | P9WKE1 | | A0RMD6 | | P09182 | | Q9AEU2 | Q6EJ98 | Q0SNX4 | | A1ATI1 | |
| B1YGT7 | Q3KFR3 | | P51306 | | A9KMP5 | | Q8Y1C6 | Q5LU92 | P0CM92 | | Q18CF1 | |
| B1JJJ5 | Q2YVY2 | | P48193 | | Q04754 | | Q4JQU7 | Q5QJC2 | Q0ADN6 | | O67820 | |
| A8EYA7 | P0CR18 | | Q4ZNI9 | | P25824 | | B0RU72 | Q8Q0A4 | A7TQK0 | | B2T9I7 | |
| P59775 | Q944H2 | | P0A4E1 | | Q9AE55 | | A0KWE9 | Q3AUQ6 | Q06FX1 | | C8ZJB0 | |
| B5FRW4 | O08644 | | Q866E1 | | A1EA17 | | Q9H160 | Q3B6R6 | Q8G0E0 | | A8F0H9 | |
| A7Y3J1 | Q9Y5G5 | | Q31SX4 | | P51607 | | D3W0D1 | P26688 | B7NGT8 | | Q97S73 | |
| Q2QLE2 | Q28S09 | | O08736 | | B7MH94 | | P55924 | A0Q0K2 | Q5E1N8 | | P0AEM9 | |
| B7M2A7 | Q2L2J6 | | Q36421 | | A0KIP7 | | Q40592 | C0ZFN0 | O83217 | | P34856 | |
| A8AIC5 | C3LVM1 | | Q3U1Y4 | | Q72EC8 | | P9WG21 | A3PGM9 | B2T172 | | P54892 | |
| B5ZBZ8 | A7FLX4 | | A0ZZ75 | | Q66T64 | | A0KJ32 | A9IMI9 | Q9ZLL2 | | Q9K1I3 | |
| A8H6W1 | P12391 | | A1W4L3 | | O28373 | | Q1CZI7 | Q81N43 | A4SNJ1 | | A5WCK6 | |
| B2SAT5 | Q9LY15 | | P46592 | | A0B2F5 | | B1KMX2 | Q46IW4 | Q8YAE1 | | Q4R4M3 | |
| Q0BW82 | P0ABF2 | | Q3SSX1 | | O81972 | | P66156 | B5Z0F1 | O84842 | | P0C890 | |
| Q9XG81 | Q8GED7 | | Q9ULM6 | | B7L0Q8 | | Q9JX03 | O68926 | Q5HN85 | | Q39I86 | |
| P0A739 | P45812 | | A0L5U1 | | Q62GM0 | | Q8D205 | B1WS35 | Q65730 | | Q4JAM7 | |
| Q3KLR9 | B8D847 | | A5CVF9 | | A8FD18 | | A4FPR0 | A6VQX9 | B1J6N3 | | B7UFU5 | |
| Q13VE9 | Q86XN8 | | Q07926 | | P17490 | | Q6QVU4 | Q8UE17 | B9ISZ3 | | Q98PY2 | |
| Q3J290 | A8ANP6 | | Q8C0C4 | | A1B4Z8 | | B7J268 | P0DH80 | B6ITW5 | | Q0C0U1 | |
| Q7N9A4 | C4ZQK4 | | A0PK11 | | Q6GAR1 | | Q08DM2 | Q8U3S9 | Q9KU20 | | A1T732 | |
| P38144 | Q03262 | | Q5R2J5 | | Q04NX9 | | Q32ZL2 | Q8N6L1 | Q3M5C9 | | Q5T2T1 | |
| Q8NWZ1 | A6NKP2 | | A5F987 | | O13395 | | Q20EV2 | A9KZX1 | P80593 | | Q49W64 | |
| P36526 | C3PAL5 | | Q2RFW3 | | A1KMP8 | | Q2G8J9 | Q9HIR2 | P58691 | | A7FLL2 | |
| P16020 | B3H2I9 | | A8F4G0 | | Q163J1 | | Q5LRA3 | Q8K1Q3 | Q7A5S2 | | B0UFE2 | |
| Q17Z26 | Q8AWC7 | | B1KL04 | | A9NAL3 | | Q47UW1 | P59150 | A1CJS8 | | A6UFC6 | |
| A1XFZ3 | Q2G283 | | Q5F3A6 | | P16168 | | B4U2K1 | A9BF00 | B2INX0 | | P29459 | |
| P62515 | P9WQ11 | | Q11XC6 | | P0C1P9 | | P84872 | B1IVT5 | P12045 | | A8AF74 | |
| B2ICR0 | P0CK91 | | P35645 | | Q9KNS6 | | Q9KV81 | Q2KE60 | Q9Y4F5 | | Q5PLB0 | |
| B9J8D0 | Q8RQM5 | | Q6LYK1 | | Q15YN4 | | B1L7Q7 | A4QYM6 | Q4P5U4 | | O83387 | |
| B1VDV4 | Q3UHQ6 | | B4ULC6 | | A5FV18 | | A9WEJ7 | A4WE78 | Q9D8M7 | | Q96575 | |
| Q1GRV8 | Q02572 | | Q84TG1 | | Q82JR9 | | C5A782 | Q83D73 | A6X0Y9 | | P0DJS0 | |
| Q9JRD8 | Q7NQF2 | | C6DH09 | | A3CRB8 | | Q8ZBB5 | Q6AML3 | B6JCI8 | | Q5XJV6 | |
| P9WNQ0 | B2RJS2 | | Q0HLZ5 | | B0UV89 | | A8T644 | C5BF79 | P59987 | | P30958 | |
| Q197D5 | Q72CG8 | | B5QXM8 | | C5D8T2 | | B8NDQ2 | Q68S19 | Q2FLH5 | | Q8CS89 | |
| Q9JZ47 | A3PCK5 | | Q0G9X5 | | P83953 | | Q6DUL2 | P0DJ22 | A8XAC4 | | B0UU87 | |
| A5UBT6 | C0M7R5 | | Q9SJT9 | | Q653V7 | | B1YC34 | Q9BM91 | A6NIE6 | | Q49Y70 | |
| B1ZND3 | P56791 | | P0DD12 | | P70917 | | P0A7R2 | Q14525 | Q10067 | | Q08014 | |
| O67086 | Q9SLP8 | | P80287 | | P06781 | | Q59SJ9 | Q0ID60 | P67975 | | A1T4S1 | |
| A4QJE8 | A4QBL5 | | Q8SPZ7 | | B8FVH0 | | Q0MQF2 | Q16AE8 | O06047 | | Q32EV1 | |
| Q5N9W4 | Q7V461 | | A6NMB9 | | A3D4I4 | | A0A374 | A4GYU4 | Q9S7C0 | | P22009 | |
| A8Y9G1 | B4RL47 | | A7HSA5 | | Q8KA38 | | B4JWR9 | Q04C16 | A7ZUL4 | | Q8ZH56 | |
| A8YVR8 | B8CQ82 | | A9VUA7 | | Q9I5U6 | | A7ZHC8 | A2AQ14 | Q0TLX5 | | P62442 | |
| B7M3W8 | B1A926 | | Q9KSQ4 | | Q3JMP9 | | A6Q1Q1 | Q2S4D1 | Q8Z8G5 | | Q1IFK9 | |
| Q0A5V4 | Q8T6B4 | | Q87SG7 | | Q3T064 | | Q2A2U4 | Q8NP03 | B3QCI1 | | A4SWG7 | |
| B2VFV0 | B6JNZ4 | | P39101 | | P34188 | | Q47FN7 | Q8BYI8 | Q4A7J6 | | A6TNP3 | |
| A1KVW2 | Q8RHY7 | | Q92QF4 | | Q6AQ04 | | P31793 | B2ATX1 | Q10415 | | P69766 | |
| Q04954 | P83585 | | Q252V8 | | B1KWM4 | | P51441 | Q11HS7 | Q01129 | | A6TRX7 | |
| Q9TLV5 | Q02SG3 | | Q4D6B2 | | Q886Q7 | | Q5RD60 | P9WQN9 | B5BJV8 | | Q0T634 | |
| Q9F664 | B5EMC8 | | A4XL42 | | Q49V50 | | A1DMT9 | Q1D2S6 | A4G8L5 | | C5CXR6 | |
| P31691 | P9WFM1 | | O70490 | | A7ZFY6 | | A4RQ11 | P63624 | Q0A8Y4 | | A6GWS4 | |
| P0A5Z7 | Q8EI02 | | Q9CLY2 | | Q8UGK8 | | P92477 | C3N5V4 | P07428 | | B9MQX0 | |
| Q9BPE1 | P38782 | | O43432 | | P51165 | | Q3BSN5 | Q7KHA1 | Q6F0L8 | | A3D1W8 | |
| P50013 | P0ACQ1 | | B1XY65 | | A9IFF5 | | B7LGV1 | Q6GE14 | Q9N2U6 | | Q732P2 | |
| B4EFI8 | Q9M4G5 | | A0KXY6 | | Q4UMK5 | | P37779 | A9MEJ1 | B7LU76 | | Q6AFQ2 | |
| Q5H6H2 | Q9HHN3 | | A9IW12 | | Q5BL31 | | Q39752 | Q6CNW3 | A6VEE4 | | Q9RWS9 | |
| B3QLF5 | C1C8J2 | | B6ENG5 | | A2BTC7 | | Q67147 | Q1MBS9 | Q9UUH3 | | Q6LU70 | |
| A5VQY8 | B3QNZ9 | | Q9KPH8 | | B1P1E1 | | Q9A7N4 | Q5M947 | B5ZUE4 | | B5E488 | |
| Q5NIC7 | Q3M8H2 | | Q9SWW6 | | A2S836 | | Q9WVC3 | P42784 | P92990 | | A9F1Y8 | |
| B1J2W7 | Q7V2F8 | | Q7RXW8 | | B1IXL9 | | P0CZ55 | P0C0K4 | A7I7Y6 | | P58797 | |
| Q089R1 | Q0TLG7 | | O54967 | | A4WVK9 | | O13943 | Q8XZ83 | Q56WM6 | | Q9J5C0 | |
| B2RJ15 | Q5R610 | | Q5M4W4 | | Q63ZV0 | | Q1RGF8 | P38133 | Q02W29 | | B7V0L1 | |
| Q09G57 | Q14GW9 | | P23905 | | Q54XV3 | | Q62814 | Q7V383 | P63194 | | Q9TUE1 | |
| B2VF71 | Q9SJH0 | | P09511 | | Q20Z45 | | B8HFN8 | D4A2Z2 | B2S146 | | B3CL87 | |
| P26904 | P83517 | | Q86609 | | B9IXC7 | | Q9JLJ3 | B5FTV0 | Q979F2 | | Q98PZ8 | |
| P0C688 | A4XBP9 | | P49355 | | Q1IDJ9 | | E3VWI4 | Q9TL29 | Q8G1U0 | | B7HJ67 | |
| Q3V384 | B1KHK0 | | A3D5Q0 | | Q32AV4 | | C8ZBB1 | O51750 | A7X1C4 | | Q6AYF4 | |
| Q65TA9 | Q5WIE9 | | Q98N41 | | P80468 | | B5YEF3 | B8IN72 | Q2NQN7 | | Q1BUA0 | |
| C6DKY5 | Q2SJD8 | | Q8R2Y0 | | A6VUT6 | | Q5PNB4 | Q68W99 | P06156 | | C3PH48 | |
| Q9CIW1 | A5VHG3 | | Q9L3Q0 | | Q8K3V7 | | P07384 | A9H9N2 | P45170 | | C1A8F6 | |
| A5UWI7 | A7NS25 | | A4XYG7 | | Q745W0 | | B5FM45 | Q96676 | P34096 | | C7ZKL5 | |
| Q06157 | Q0A9D7 | | Q9JLJ2 | | Q89AZ2 | | Q3KIZ0 | B4R8L7 | P09339 | | P24311 | |
| P92564 | B7H979 | | Q7V2F9 | | Q62JC7 | | Q63UY3 | P41550 | O80337 | | Q7VXY3 | |
| P0DL33 | Q28Q60 | | C1DFK0 | | P57185 | | Q8U8Z7 | Q057W1 | A6NCQ9 | | Q5FN26 | |
| C1C6Z5 | P28506 | | A3N0A3 | | P23279 | | P27704 | P30667 | A0QPF9 | | A7HNY1 | |
| P11764 | A7H3U9 | | P08220 | | A4VHM9 | | Q1C1T7 | Q75AZ8 | Q8G595 | | Q55BJ6 | |
| C1CQ04 | A5EAA0 | | A4FW85 | | Q1MRV6 | | P53954 | Q836R0 | P20784 | | C4ZSX8 | |
| B7MSH6 | Q39T44 | | P38509 | | Q31VW3 | | C1FLA5 | A0LRM0 | B5ELY4 | | Q0VCX4 | |
| E1B2Z9 | A7G9P2 | | Q5ZKK7 | | A7X1E9 | | Q2UDE5 | A4GCK6 | Q8BGX4 | | B5E1U3 | |
| A4IWY2 | B1JDU9 | | Q6L8Q7 | | A8A3L2 | | Q89AM4 | Q69UP6 | P01180 | | B4F2M5 | |
| B0RZP8 | Q3K9X0 | | Q9HLQ1 | | B8NIM7 | | P59663 | P40884 | A8C8X3 | | Q65CC1 | |
| B8JAN4 | A3LRW2 | | Q087L4 | | Q07WF3 | | Q5M079 | Q0VM18 | Q0SNB9 | | Q9M5K3 | |
| C3LRG7 | P47280 | | Q9EVV4 | | Q6C3X7 | | P33501 | Q2RFQ2 | P79338 | | Q9CR58 | |
| P81928 | O07177 | | Q04LK1 | | O01501 | | Q2JL69 | A4QJM3 | P73532 | | Q7VYB9 | |
| Q7MX21 | Q8UDF5 | | Q1LT27 | | A6X0B0 | | P41199 | Q486Z0 | Q6DQX6 | | Q5B3K6 | |
| Q92838 | Q8F8Q2 | | Q2J2C3 | | C0R214 | | P0CE15 | P27526 | Q498D5 | | P0ADZ0 | |
| B5BIS8 | B0BC73 | | Q72NH3 | | Q69028 | | Q11HP7 | Q9M8R9 | Q0UTE1 | | B1WU13 | |
| B4TN36 | P29881 | | A4QLE1 | | Q8XVC2 | | P69679 | Q63PF1 | A7NHN1 | | B3PTW0 | |
| B7UPE1 | Q9PLS0 | | Q01644 | | P11421 | | A3D7C5 | Q39V70 | Q2IGX5 | | A1WJ05 | |
| Q5M140 | B0TX64 | | Q9BYD2 | | A7GE89 | | A9MU47 | A1JML4 | A2BTM4 | | A2BLY5 | |
| Q39W61 | Q9WU60 | | Q704S8 | | Q88CA0 | | Q9LNI2 | A6VIC0 | Q6CT90 | | Q9TNB1 | |
| Q42522 | A7ZSL0 | | A1VDC3 | | Q965W1 | | A2SQJ8 | Q9H8Y5 | Q4SPU8 | | B6DZD2 | |
| Q313K0 | A4Y6H0 | | Q03ZP2 | | O88823 | | P24452 | A0LRN6 | Q8FBI4 | | Q5TBE3 | |
| Q9HWC1 | Q9FJQ2 | | Q03DS2 | | A8A731 | | B4SVD3 | Q57PU7 | Q9YB51 | | A5UTJ6 | |
| Q9WX62 | Q5P791 | | P0DH89 | | Q8XIU9 | | Q8DF92 | Q05718 | Q0ASJ0 | | Q6MED0 | |
| Q9C9N3 | P0A472 | | Q8Y6Y9 | | A4D9B6 | | Q1QRH1 | B5RRJ5 | B0K9M0 | | Q8E9D9 | |
| A3Q0T3 | P08493 | | Q13938 | | P11442 | | Q14F99 | Q38S38 | Q7N799 | | B3DMA2 | |
| B6YWY1 | Q0SS00 | | P53348 | | Q52281 | | A7Z4X2 | P0CJ96 | Q4HW67 | | A5V807 | |
| P67054 | A8LKJ4 | | B0BK70 | | Q2FZ72 | | Q922S4 | A2X0Q3 | Q33C08 | | P10378 | |
| Q7VTA1 | Q1QLI8 | | O42778 | | Q5XA92 | | A1A3P5 | Q0KCJ6 | C4KHB6 | | A4G647 | |
| P0CM81 | Q1MTD3 | | A0KGD2 | | A4QJF6 | | Q96474 | B8E4K7 | A8Y9G5 | | Q2FT98 | |
| Q6HEU5 | A0RIY7 | | Q21KW2 | | D3PW16 | | Q68CZ6 | P0AFI3 | Q5WGB8 | | B2ICL6 | |
| C0ZW28 | A9KJL3 | | Q498S8 | | Q0RQV5 | | Q3SHT9 | A5CEL6 | A0M4U6 | | Q9FJD5 | |
| Q9HX17 | Q1PF16 | | Q5F791 | | P69709 | | P60781 | A6ZZS6 | Q64640 | | P69073 | |
| Q7UC29 | Q9S9N4 | | Q9M4B5 | | Q5FU06 | | Q18CI7 | Q8ZP00 | O35532 | | A8GRJ5 | |
| P39404 | B4S5K3 | | Q8THJ7 | | Q54XW7 | | C7GTL7 | A2C6S9 | Q3SHL2 | | Q07Y78 | |
| Q88FY5 | Q6FZL2 | | Q6DDT1 | | A7Z9Q0 | | Q2JUA5 | Q2NVL1 | Q6UXX5 | | Q6ING2 | |
| Q6D066 | B0CKN4 | | Q02NA9 | | A7HCI0 | | Q7WCK0 | P66164 | P59279 | | A2C0H1 | |
| A0KZM0 | A6VIQ1 | | B0RIJ3 | | Q1XDJ3 | | P0C9C3 | B5Z492 | Q1ACL0 | | A1S1K6 | |
| P0C0Z7 | Q974T1 | | A7SL20 | | A8FJR1 | | P26471 | Q7VVF1 | B1ITC8 | | Q65694 | |
| P20937 | Q73KZ3 | | Q39WF6 | | Q6BXL8 | | B0S187 | Q962R9 | Q3Z598 | | A4G8R1 | |
| Q4ZYW8 | Q62785 | | A7ZK14 | | O49298 | | P67543 | P04061 | B8EES9 | | Q6EUH7 | |
| B7KGB9 | Q6G3K8 | | P95724 | | Q95L39 | | Q21QN5 | Q32RV4 | Q49B96 | | C5BED3 | |
| P06303 | D2HEW7 | | Q1RB47 | | Q7ANU3 | | Q9H2C2 | A4QKT9 | B0BUP4 | | B5RMX8 | |
| C0ZIJ6 | A1KUL0 | | Q65JJ8 | | Q9CCP3 | | B9DQ42 | A6T4Y3 | Q1D7U2 | | Q9P209 | |
| Q1QMM7 | Q3C1M2 | | Q5JEZ8 | | Q72L76 | | B7LJY1 | Q5H6B9 | Q9QWZ1 | | C6C0D6 | |
| Q8MJA0 | P19946 | | Q2JRG2 | | A7MQ65 | | Q2T3K6 | Q9VKB4 | Q0K0V6 | | A6UUY1 | |
| B3TN49 | Q0ZUP0 | | P68546 | | B4U4X8 | | Q5HG19 | Q54GG9 | B1HR71 | | Q4ZWU3 | |
| A6WHS1 | P18605 | | Q5HGJ3 | | Q60952 | | Q0T8F7 | O63057 | Q64240 | | B1JJ36 | |
| O17554 | B0REV4 | | Q8VUS5 | | Q60CV0 | | Q6BGV5 | B1X503 | A5I4K9 | | P29643 | |
| P0A6T3 | Q8NVG4 | | Q09886 | | B1YMW5 | | C0RJC9 | E3E2E2 | B8CZR7 | | A1CHP1 | |
| A7ZTY0 | Q2P757 | | A0KQA7 | | A8Z2R1 | | Q0ACQ8 | Q5HC04 | Q9JRT1 | | Q9CDV7 | |
| A6MMG1 | A0KIC9 | | A1C4R7 | | Q9QY15 | | Q2NW52 | A4VRN4 | Q74IT6 | | Q5L3Q9 | |
| Q668T8 | Q1GAG1 | | A3NEF2 | | A3NQ33 | | Q7M840 | A6U848 | Q4KC79 | | P22805 | |
| O32886 | P0A6U9 | | B4RVP7 | | Q2W2K3 | | A5UA09 | B9IT27 | A4G0J0 | | P28564 | |
| Q0HDL6 | A8G8E7 | | Q1LLI8 | | Q3YVZ9 | | B4E5B5 | A0RRR3 | O50635 | | B1VXY2 | |
| B7HJ47 | P9WH86 | | Q65JB2 | | A6TY54 | | O35205 | Q8XHT4 | Q8DBS9 | | O47043 | |
| Q8XBV9 | A8YXJ9 | | Q2UU13 | | Q3K5T0 | | P45841 | Q5M108 | Q9BY08 | | Q5ZVI4 | |
| C5DJL0 | Q196X8 | | Q9BM95 | | P42131 | | A3CWM4 | Q8TRZ6 | Q145P6 | | Q6GZP9 | |
| A6Q1I6 | P30073 | | A6Q1M3 | | Q39QB0 | | P05371 | Q215X2 | Q5QVF0 | | Q88QG5 | |
| Q1BUF3 | B7V1F8 | | Q2SU22 | | P01329 | | A0RQZ2 | B3DU42 | P31135 | | Q837A8 | |
| O32178 | A8EZT1 | | Q8L936 | | G2K3G6 | | P69636 | Q9HHJ7 | B5XY59 | | P19700 | |
| Q5Z1Q1 | Q5JH91 | | Q9X5F0 | | B1JV80 | | A5D9D4 | Q97B25 | Q7VFZ3 | | B0WAU6 | |
| Q5YS64 | P72186 | | O05953 | | B5YG55 | | A3CPY9 | P9WI46 | Q6NPR7 | | Q02VZ1 | |
| Q71UG0 | A0JNC3 | | Q15MT8 | | B5XMZ4 | | Q8NTT4 | Q8UEZ0 | Q4CY36 | | A2XN66 | |
| P02014 | Q694C5 | | B7UK32 | | Q6UWU2 | | Q8F353 | P99087 | A4FQK6 | | Q3MHU5 | |
| Q7MK59 | B0G100 | | A8GAX8 | | Q747W9 | | Q5HC08 | P29130 | B8IZS6 | | Q92RG8 | |
| Q32PI9 | A6WMW8 | | B5YSF1 | | B7NT79 | | A8HDJ7 | Q8D2P6 | P0DM41 | | Q99PF5 | |
| A5IHU3 | C1FVB2 | | Q1LTN7 | | A6LY33 | | B8E9P2 | Q72PM6 | O46634 | | Q1XDF6 | |
| Q9XED7 | Q10487 | | O35847 | | B5E3L5 | | Q6YNB6 | Q5HM17 | P30345 | | Q6HAU4 | |
| A4VIT1 | Q54R52 | | A0QJ19 | | B1VG90 | | Q8TD57 | B8D5V4 | O82567 | | Q332T8 | |
| Q5WVX4 | P48623 | | A5IK69 | | Q39CE5 | | Q9GZP0 | Q4KTG9 | A7ZK57 | | B0UV09 | |
| Q1QVF0 | Q8NT26 | | Q0HT72 | | B1KV16 | | A3LP72 | A8MH75 | Q48PJ8 | | P0AFC9 | |
| B4EV01 | A4WVG1 | | Q0SN37 | | Q5N159 | | Q48VY9 | B5EFG4 | Q5U2R4 | | Q324F4 | |
| A1VJ38 | Q87BL1 | | Q5QV40 | | P0A5I7 | | P0A681 | P38737 | B6JAR2 | | Q83LP1 | |
| A5EX42 | P52001 | | P11539 | | P0A406 | | Q18CF3 | Q88QL7 | B5RLU8 | | P28578 | |
| D5SGZ8 | Q28462 | | Q06R93 | | O80365 | | P03462 | C6DB25 | P66715 | | Q6GF17 | |
| P63052 | O23254 | | Q9PCG5 | | Q68FU7 | | Q9H079 | Q0W278 | P37735 | | B1IR88 | |
| P03970 | A7FGU7 | | P15231 | | Q07KL9 | | Q980Q7 | Q4ZMN7 | O00115 | | A1B3U2 | |
| C3MQ62 | Q3IUD7 | | Q9SHV2 | | A4X4C8 | | A4TSD3 | Q73F78 | Q3Z788 | | A5CCN0 | |
| Q5N5X5 | Q5ACW8 | | A6U3V7 | | B2K7I5 | | Q2SXU8 | P0C0F3 | P14156 | | Q9UP38 | |
| A7M985 | P19587 | | A5GAW7 | | Q12563 | | Q0VKU6 | B7LUS5 | B0BQZ5 | | C4LA09 | |
| Q99MZ6 | O85282 | | B5LMR2 | | Q9EQI6 | | P56687 | P51351 | A6WCA2 | | A5F3P5 | |
| Q9STD3 | P29118 | | Q8K585 | | B7MB08 | | B0Z5D4 | Q1I640 | Q9D0N7 | | A8M746 | |
| Q17QR5 | O86436 | | Q45535 | | Q5UP16 | | A0B6I9 | B9M2A3 | A1TJ06 | | Q8CH27 | |
| Q33954 | B4SKV5 | | A4FUZ0 | | Q3STQ8 | | A1RMC9 | A1KHB7 | Q4PM54 | | A3PIV3 | |
| B5RFY5 | Q5HUY2 | | A4SYD1 | | Q975G7 | | B9EB51 | B1IPZ1 | A3DNI1 | | Q75AF9 | |
| Q8CSZ5 | P59335 | | Q8K2J7 | | Q05B44 | | P0C8Z1 | Q820Q8 | B5FHG8 | | Q6GZW7 | |
| P01132 | B1YSP3 | | Q1M4A1 | | P87297 | | P59584 | Q830B9 | C5CGM3 | | O28359 | |
| P99076 | A5G270 | | C3LQ29 | | B5FF31 | | Q8R1S0 | Q06115 | A6QIG7 | | A0RBC9 | |
| A1AJE9 | Q5X5E6 | | C1D8A6 | | Q14681 | | Q6GKG7 | Q5QUN6 | B5BAW8 | | A9NC23 | |
| Q2V4A3 | Q7ZWV3 | | B8ZPW3 | | A4FVY0 | | B0UD19 | B2S2N1 | A7WYW4 | | Q5BIW4 | |
| A8GM54 | A6LJ74 | | B8ERB9 | | Q1RGM9 | | P60015 | P59327 | Q1R3P3 | | Q7SEY9 | |
| A7GV47 | Q4ZZ15 | | Q05492 | | Q32KU9 | | Q3JT10 | C3MWN2 | Q1BZH2 | | P0A3J0 | |
| Q8KF03 | O13439 | | O55727 | | A3LXM4 | | B7LRS8 | B5ENA2 | Q8CGK5 | | A6TFP8 | |
| B6YXW5 | Q6PBN5 | | Q588G0 | | A1WPR4 | | B6J202 | C3MZB3 | A8F4E1 | | B8FBE8 | |
| Q62M76 | Q7MHV3 | | B5BI74 | | P48665 | | B4HEM4 | Q1XDF5 | P50523 | | Q5WSE7 | |
| A2C9Q5 | Q01655 | | Q06J70 | | A7ZVE0 | | D9SEU0 | Q8R690 | P19168 | | O84839 | |
| Q988H0 | Q83AL3 | | A8I464 | | Q8Y494 | | Q96G25 | Q8G443 | Q22021 | | Q0TJE1 | |
| P0C456 | Q9RVB9 | | B3Q972 | | P0DJU5 | | A6W5V4 | A4SJR3 | Q62052 | | Q82DX1 | |
| P37037 | O73819 | | Q1C9C5 | | Q9K0I1 | | A3GF61 | B3SVF1 | Q5ZYN5 | | B7JIL4 | |
| B9KP62 | Q8XZ91 | | Q30Z47 | | Q9PNX5 | | Q1AXJ9 | Q6MSN8 | O69014 | | Q1KXS6 | |
| Q24U35 | Q01205 | | Q28944 | | P80015 | | Q9C8T9 | Q0MQD6 | Q6HPQ5 | | P77528 | |
| A8Y9C2 | O82139 | | Q8UBS4 | | Q2RFP8 | | Q1RGG1 | B0TIR7 | A6VLJ8 | | Q8T6J5 | |
| A5FQE0 | Q1JGX1 | | C6E515 | | Q8PC48 | | P22791 | Q57LV0 | C3NJ74 | | Q3AYI2 | |
| B7GNK2 | Q2HXL6 | | Q9A246 | | B5BE12 | | Q09FS4 | Q8ZT50 | Q6LXY6 | | A4G3H2 | |
| B5RG99 | A7Z4N5 | | P01063 | | C3N5U4 | | A5IN74 | Q43189 | P0CZ93 | | P40941 | |
| Q9I0A1 | P02141 | | A7H5T0 | | Q6KF82 | | Q65XK0 | Q1E0W9 | A5VSP2 | | O16027 | |
| Q82TV1 | C0RJJ9 | | Q3JVW9 | | Q5L5F7 | | A6T6L0 | B8DDR8 | Q1CFF9 | | C1CRA4 | |
| B9E8F5 | B4PN49 | | Q8DMM6 | | A4WR94 | | Q8TTA5 | Q49V52 | C1FUF9 | | P83122 | |
| B3DL65 | P08115 | | B7JQ65 | | Q71WN5 | | B5EBP9 | Q5DZI1 | Q89FU7 | | A7ML12 | |
| Q0IIG8 | Q250P7 | | A2BTD2 | | P69661 | | O13670 | A4XWT1 | Q9DBZ1 | | A6Q5D0 | |
| A1REA7 | Q05682 | | Q07660 | | B0RY33 | | Q1WTX5 | Q6F8G1 | Q0UL22 | | P10091 | |
| A0ZZ24 | Q1BR30 | | P27730 | | O35623 | | P49667 | A1JNN2 | A9L4G4 | | Q98TX3 | |
| Q1IF15 | P53550 | | P22002 | | C0ZIJ0 | | Q9CNT7 | Q4UJR2 | Q5R652 | | C3K2X8 | |
| B1KZN7 | Q8R1F1 | | Q5UKY4 | | A9VIV4 | | Q892J5 | B3H1B0 | Q0TRD6 | | Q971I1 | |
| B1Z9R0 | B2IUT0 | | P40487 | | P43396 | | Q2JV88 | P97474 | B7K1N1 | | P25042 | |
| A9VKI1 | Q9AC54 | | B4SKX8 | | Q9CQV4 | | Q800I8 | Q9P2X8 | O00822 | | P04256 | |
| A5CWW1 | E2QXF3 | | B4RBN1 | | O54889 | | Q5X499 | B8DZY2 | B4UN83 | | B5Z8J6 | |
| A8Z4L2 | A4TMU0 | | P46898 | | A1JS00 | | Q09WX1 | A4YB21 | Q8R5Q4 | | C1C6S4 | |
| B5RM48 | Q92GW5 | | Q7ZVF0 | | Q6J756 | | P48856 | P38131 | Q6YQT9 | | Q1MTQ5 | |
| Q96HE7 | A8GNL2 | | Q79VE2 | | P31195 | | B8E0X3 | Q8FSD4 | Q8IRB5 | | Q8FR24 | |
| P06379 | Q9ZHE5 | | B9LTT8 | | Q47G67 | | A9ADI8 | B1X9B7 | Q04WK1 | | P27683 | |
| A3PFJ6 | B7IUM3 | | Q8PC67 | | A9R397 | | B7HK47 | Q18823 | Q47HR2 | | Q75JG8 | |
| B1IXM2 | Q05183 | | B5XZP6 | | B0T663 | | Q85FI4 | B0TL65 | Q3YZ81 | | Q12669 | |
| Q9FDL9 | Q9TH43 | | Q8IFJ8 | | P9WPG7 | | Q32KY6 | C4ZQQ5 | B0RZJ9 | | Q9ZQ77 | |
| Q8NQJ3 | B8DB20 | | A8AXN9 | | Q1ACF5 | | Q2FHG3 | Q1ACF7 | A4WNY1 | | P30562 | |
| B5BGY1 | B6KV60 | | Q3A8L3 | | B7VLM0 | | Q0TMS6 | P21327 | P25200 | | P48705 | |
| Q5EA15 | A4W9U3 | | A5FRX2 | | O29108 | | A1ALT3 | P0A2C9 | Q57969 | | B4SRB3 | |
| P04293 | Q5PLB4 | | A8HDK4 | | B0TC68 | | P33855 | B5EKW6 | B5EXF8 | | Q53EP0 | |
| A0K973 | Q5UAS1 | | P0CP59 | | Q14FB1 | | Q46I76 | P45870 | Q3SWM9 | | C3P4E3 | |
| P17491 | P94612 | | C4LID1 | | Q82SQ2 | | C6DF43 | P64392 | B1I8K3 | | O35350 | |
| A9R563 | A0LSL5 | | Q2RVU4 | | O57286 | | B0RNP8 | Q4UW55 | B7GW01 | | Q0AUD4 | |
| Q6LN63 | A1TKD3 | | B7VKD0 | | C1CTL3 | | Q6Q487 | Q255Q8 | A8GXI1 | | C6A267 | |
| Q1LJ60 | Q48VV0 | | B1JDX2 | | A2XCT8 | | P95907 | Q3AQN3 | B4TAI5 | | A1BE85 | |
| Q46EW4 | Q98234 | | Q9Y718 | | Q90873 | | B4T110 | Q8RAL7 | A8YXM6 | | Q6J8I9 | |
| P0C9Z2 | A1WU91 | | Q6V9H4 | | Q57PW5 | | Q2V2Y0 | P20778 | Q75BV8 | | Q9PE51 | |
| Q2NI83 | Q5HDW8 | | Q6G9T9 | | Q8PNU4 | | B0VBD8 | Q7XJL2 | Q9CPY1 | | Q4JUJ9 | |
| A5GVY8 | Q5RER2 | | Q9Z6Z6 | | P57237 | | O78451 | P04959 | Q5ZJ87 | | Q8BZQ7 | |
| Q5ZIP2 | A1W327 | | Q38S00 | | Q99075 | | Q46I40 | P43909 | B1IA36 | | A1BHZ7 | |
| Q7WG65 | Q34890 | | A9MIH3 | | P46273 | | P22621 | P17513 | C6C1A9 | | Q6MEB3 | |
| A4YSI0 | Q9ZAE3 | | Q9UZ09 | | B8HNK4 | | Q3K5S2 | B1LGQ0 | Q5LRY0 | | A7HZK8 | |
| Q02X10 | A5UL69 | | B2LMI5 | | P38192 | | P22922 | Q9HWD5 | Q1KLY6 | | A6YIH8 | |
| A2S7G4 | B5R297 | | P35605 | | Q477I0 | | A0L8G8 | Q0AUG7 | Q12AK6 | | P00241 | |
| A6NNB3 | O03554 | | Q9C0J1 | | Q1IT15 | | Q8R5Z9 | P79853 | D7LAP2 | | Q6YR16 | |
| Q6QN06 | A0L5Z1 | | Q9CDW7 | | Q9MSA0 | | Q03D70 | B1II67 | A7ZB87 | | Q6BRB4 | |
| Q82MI6 | Q9RR70 | | O64988 | | A0KJD6 | | Q3J1A0 | Q4A7B0 | P50942 | | P03858 | |
| A8AZM6 | Q3ASW4 | | Q554S6 | | Q64413 | | Q2TBR2 | B6SFA4 | C4QYK3 | | P19244 | |
| A9W605 | P97434 | | P38424 | | A5VR14 | | P05744 | Q9R1T5 | P00869 | | Q71YP9 | |
| P52323 | Q0HKD1 | | Q9FJV1 | | Q5M101 | | Q70JZ1 | Q2GL35 | B5XNL1 | | Q8K3A6 | |
| A3PK84 | Q0I284 | | B1VET5 | | P53901 | | Q6DGP7 | C4R702 | Q9HJY7 | | Q3V4Z3 | |
| Q09FY4 | O82798 | | C4KH13 | | Q5HTX9 | | P27903 | P0C8M6 | A2SSV2 | | C6BYG5 | |
| Q98PU8 | B8GET9 | | B7NJ75 | | B2U1Z8 | | A5IYR6 | B3E7T5 | Q17XN2 | | O36052 | |
| P94693 | Q6TK73 | | A4XPY6 | | C3MJ04 | | A3D7X8 | Q8Y7J7 | P51592 | | P57415 | |
| B2ICQ8 | P57722 | | Q4URK1 | | B7I5H1 | | C5C3P2 | B7MBD7 | B6J8M6 | | B0Z4T2 | |
| P44524 | C5D428 | | Q132N3 | | P90866 | | A1KB30 | Q6GPQ5 | P27165 | | A8F204 | |
| Q66H23 | B7NEV3 | | B3W7G1 | | Q62813 | | P12111 | Q9SGC1 | Q8CDD9 | | O60299 | |
| Q9GKW3 | P10503 | | P17099 | | A5GIT3 | | Q72AL5 | A3CT18 | Q8YES8 | | Q63X63 | |
| Q3SLQ0 | A2BUU2 | | A6VGH7 | | P9WFR7 | | Q9ZJV9 | Q9CR30 | A2RML8 | | A6ZQK9 | |
| B1XJS7 | B8IS79 | | P0A9D0 | | P0ABN3 | | C6E4R3 | Q96BT7 | Q97F39 | | P12911 | |
| P24590 | P36921 | | Q9BPD5 | | P14674 | | B4T4Z4 | Q3U3N6 | B2UEL8 | | C6E0V3 | |
| Q3YV71 | Q3T0U2 | | B5BGY7 | | A6WUJ0 | | Q9DXE9 | Q61839 | B7NMD7 | | P47176 | |
| B2V966 | Q03PW9 | | Q49401 | | Q6NRE4 | | Q7MXW3 | B3EP58 | Q8CPK0 | | P92565 | |
| Q55179 | B5XUR6 | | Q142G6 | | Q4JC71 | | B8H618 | P46651 | B2FL98 | | Q5FW42 | |
| A6UE65 | Q9D7M8 | | A6TH65 | | P73737 | | A7ZK12 | Q87QW9 | A9BCH5 | | B8EQH6 | |
| Q95P08 | B0KW95 | | P9WQ26 | | P9WMK8 | | Q2FJ92 | Q9V8I2 | P51310 | | Q652I1 | |
| A8G742 | Q4FNM9 | | B5R0L2 | | P11217 | | Q85FN0 | Q1D760 | Q3KJA1 | | Q4YUG2 | |
| A9KGD2 | P9WGY5 | | Q5W0A0 | | Q6IE62 | | Q87N20 | P47407 | Q5HQ98 | | Q7MI38 | |
| A0K1X6 | Q8TJC5 | | P12538 | | A8AB39 | | Q5FG66 | A5F5N1 | Q18G62 | | Q9ET22 | |
| Q927M3 | B3PC16 | | A4W8S9 | | B4N549 | | Q2L2H8 | P35014 | P60619 | | Q2FF20 | |
| B0RVE1 | A7GJ81 | | Q8K3H7 | | Q97I63 | | B7N247 | B7LI05 | A1VDM6 | | Q57MX5 | |
| Q87N44 | P0CH12 | | P61276 | | Q98GS0 | | Q6LUJ4 | Q67PB4 | B7NT96 | | P56959 | |
| P14972 | Q7VJ10 | | Q02643 | | Q02LX5 | | A1VXH8 | B4RPW8 | Q21939 | | P0C568 | |
| A6UQK8 | Q9L6B7 | | A9MNW8 | | Q54ED7 | | B5ZRL6 | Q8PGG7 | B5EMD4 | | B0KK66 | |
| Q9L1A5 | Q931I4 | | Q71YH6 | | Q7N760 | | C1AVG2 | A5WD02 | A5V282 | | B6ENR7 | |
| A1WVB0 | Q3EAF8 | | B2T2S5 | | Q3JPY2 | | B1KSK9 | A8GT64 | Q71L16 | | P35991 | |
| Q57M43 | Q4K5G0 | | A4WKL2 | | B0Z4M8 | | O22644 | Q2LX49 | Q8UEY7 | | P57975 | |
| B7HKD3 | Q04912 | | A8XAA9 | | B5RH30 | | B2AWD5 | B1JUY7 | P62593 | | Q31QF6 | |
| A6UZI7 | P65794 | | P23311 | | A1CSI0 | | Q5M158 | C1AMX6 | P83946 | | Q5R824 | |
| Q2JH51 | B3QYL5 | | A2A7Q9 | | B1X9Z3 | | Q559A9 | Q2NVF9 | B5YS74 | | Q9HLX7 | |
| Q0CUG5 | Q8YFY2 | | C0QBX7 | | A6U8Y7 | | A6QPB3 | B7I9V2 | Q94793 | | Q91Y44 | |
| Q5I2W8 | O50098 | | C1CZB4 | | Q8ZH78 | | Q06FN2 | Q36570 | A9KXI8 | | Q63T02 | |
| A7HM34 | B7NBB1 | | Q2IW81 | | B2JHF9 | | P0C0D6 | Q5ZKQ5 | A9I1G8 | | O52335 | |
| A1CSR1 | Q5N1N6 | | C3N7W8 | | B0UPJ6 | | B5ZR94 | P0AB69 | C5A2S7 | | Q6GPJ4 | |
| B3WE42 | Q73XU8 | | Q6ENV2 | | Q146Y5 | | Q7U4M0 | Q9M6K1 | P75206 | | Q9LFU1 | |
| P60643 | A0A324 | | P0CM50 | | B8E029 | | A5URA7 | A2BXQ2 | A4SW76 | | O94822 | |
| P28403 | B0T065 | | B3QL68 | | Q49Y40 | | O84410 | P0ACZ8 | A4YIN3 | | Q10131 | |
| Q2FK01 | Q2G2R4 | | Q91FD5 | | B8GN57 | | A4XNB8 | Q44337 | B8GU25 | | A1VFS4 | |
| Q9D0R8 | A2CCJ0 | | Q66EW0 | | A8HA69 | | Q8XNL5 | O12985 | B8J1A6 | | A9M3H7 | |
| B8FUN2 | A0PZZ5 | | Q03611 | | B0T155 | | C8ZG19 | A5UHS9 | C1EZA3 | | Q97WE5 | |
| Q6J3Q7 | P54663 | | P28153 | | A7A270 | | Q6FNK8 | A9N8B8 | O87905 | | Q984U0 | |
| Q42510 | Q8LE43 | | Q9CKW6 | | P49148 | | Q92Q98 | Q75AP6 | P19387 | | P35318 | |
| A0RIB9 | A5GNJ0 | | Q04MR3 | | Q3MIC0 | | Q30P06 | Q35905 | P0C1T5 | | Q53EZ4 | |
| P02877 | Q9NXI6 | | Q8CXF3 | | Q8X5T5 | | Q77SJ8 | A5GWH2 | O14519 | | B1LQG8 | |
| B0RWC3 | Q6AJZ3 | | Q4R760 | | Q821L7 | | P02517 | A2S5U5 | P0C925 | | Q1RHP4 | |
| Q0BKK2 | Q8ZDG9 | | B5EWK6 | | Q5HC01 | | B5FY93 | A7TM20 | Q17043 | | Q4R6N4 | |
| Q9NBK4 | Q6G2W4 | | Q08598 | | B7HY99 | | P82268 | P51408 | P0CM43 | | F5CPD3 | |
| Q83WS0 | Q922R1 | | Q55BI3 | | B5Y305 | | Q9HQ29 | Q1RJ81 | Q99X73 | | A0R3E0 | |
| P01769 | Q660R1 | | A9FHA5 | | B6JAU5 | | B9L729 | Q5RFK5 | B6JPI6 | | O42824 | |
| Q812X3 | Q04211 | | B2RLY1 | | Q5ZLY0 | | P0DH53 | B7UI81 | C3PBN9 | | Q4VBT1 | |
| Q4AAJ5 | A4R254 | | B3PK27 | | P58551 | | Q1DCV9 | P40069 | P01584 | | A8A7E3 | |
| Q55DS4 | B1LL75 | | Q6IN01 | | Q7W609 | | A5GAW3 | A3CWL3 | O59229 | | P04871 | |
| Q0BXU2 | B4TUM3 | | P0AA87 | | P82718 | | Q44473 | A0LWX2 | Q8TGA9 | | Q55286 | |
| P0A313 | A6ZKM2 | | Q197C0 | | A4QKJ4 | | P0DJI4 | Q3V4Z8 | A8Z5H6 | | P00675 | |
| B4S3Q6 | P16209 | | A6QFW1 | | Q3SH26 | | P15364 | B7HER4 | A6UQM0 | | P56715 | |
| Q8YGK3 | Q92Q63 | | E2E2P0 | | Q4K9V1 | | B6HZA9 | Q8XCJ9 | A4SXQ7 | | Q5UYI1 | |
| C0MCC9 | Q5RER0 | | Q9JUV1 | | Q55346 | | C5CKT2 | P39864 | A5V9P8 | | Q0G9R4 | |
| Q6PBK8 | A9MSY2 | | Q53636 | | Q12P12 | | O22622 | A0KJJ9 | Q18014 | | A6Q4C4 | |
| Q5Z0Y0 | B1LBJ4 | | C4LL45 | | O60198 | | B6H1I3 | Q9UUI1 | A8G3N6 | | B9WZX7 | |
| B7M0E7 | Q601T6 | | Q38UU0 | | Q5B995 | | B9DSA4 | Q3K0S1 | Q3A3X3 | | Q6Z808 | |
| Q87TT0 | P54353 | | B9M4M5 | | Q135X7 | | B1XXP1 | A9R803 | Q3ILL1 | | Q3ZJ40 | |
| Q1REF5 | Q3AG75 | | Q8E7J7 | | P61717 | | Q12ZS5 | Q39KH7 | Q8X8U9 | | C1CC07 | |
| Q12Q43 | Q0BE16 | | Q48KX6 | | Q9SG80 | | Q7A471 | P96590 | B2IVU1 | | B6JM59 | |
| B5YY27 | A1KGF0 | | B0JHZ1 | | Q88886 | | B3Q7C4 | Q9EPH1 | Q9B8D0 | | Q8CFV2 | |
| B5FJX0 | Q03K83 | | Q6NEB7 | | O42410 | | Q03479 | Q9C544 | Q54XE8 | | P40091 | |
| Q73B32 | P42148 | | B4SC79 | | Q135C1 | | Q9PEI4 | P0AG44 | Q87UB2 | | P00248 | |
| B7HAZ0 | Q2GI73 | | Q1CD04 | | A7HBI8 | | P71398 | A4XI64 | B7GVQ9 | | B7MRB2 | |
| O55710 | Q96FC7 | | Q9RFD5 | | Q07DX2 | | B0BCG5 | A1R3H9 | Q8NZW9 | | A4JR39 | |
| C3MUV8 | O83549 | | Q8YGV9 | | Q8XKK2 | | Q1IHD2 | P22287 | P27166 | | Q9R9R6 | |
| P09630 | Q8DTC7 | | Q07123 | | Q5GFD9 | | Q588Z1 | Q9BPI4 | C4ZVM2 | | A7EBU5 | |
| Q9SJ24 | A8H0D4 | | A7Z4L2 | | A4VYP1 | | P68547 | B5BIE3 | B2TU88 | | Q99217 | |
| Q3ABL2 | Q3UVC0 | | Q971Y3 | | Q0I0V3 | | Q11BC8 | Q9ZUZ2 | A5N8J0 | | B1HTF6 | |
| A4QLX2 | Q197B5 | | Q1W0Y2 | | Q9HVQ7 | | Q6MTC8 | Q0I203 | A1A7V0 | | C5H429 | |
| Q8RXA8 | A3N122 | | A5VT38 | | Q1PEC0 | | B5DFC9 | Q4VNZ5 | Q28G26 | | P30390 | |
| B1J0R7 | Q3K5Y7 | | A4FLW0 | | O13889 | | A7N654 | Q53QW1 | P0C9I5 | | Q8W926 | |
| B7JL13 | B2MW51 | | Q48PK6 | | A7IA93 | | B2Y1Y2 | A3MRW4 | Q2YSI3 | | P0A7E8 | |
| Q6BNQ5 | Q9UBX8 | | A8G759 | | Q0ANE2 | | Q5YPG4 | P45120 | Q7NS41 | | A5IQ94 | |
| Q2HJ51 | Q1BXP0 | | Q54W20 | | P20715 | | Q8P1J2 | Q4ZW75 | P50673 | | A9IS43 | |
| Q1L6Q1 | Q9HIS4 | | Q3AUJ6 | | P56848 | | Q06GS1 | B3QM33 | Q839J4 | | Q42377 | |
| O49397 | Q8STR8 | | Q6GEN7 | | A6UPR3 | | A8FMZ7 | B1JHZ5 | Q255T8 | | Q8VE91 | |
| A1U342 | Q0RKL9 | | B5E7P8 | | P09660 | | Q05670 | P14808 | Q18GH1 | | B2TN76 | |
| B8HQ34 | Q5WVJ1 | | B7MNJ3 | | Q741G7 | | C4LJM5 | Q8L6J2 | Q9IB75 | | Q0I2W9 | |
| O29987 | B6EH41 | | B9L721 | | Q8Z6R6 | | P52827 | Q9HQC2 | B6VJS4 | | A6TT61 | |
| B3QAA3 | B0RVK9 | | C1C5H4 | | B7GL45 | | P58329 | Q7V9L5 | Q1KVT5 | | F4HTQ1 | |
| Q9CPR5 | Q9CZ83 | | A7ZY11 | | B4S113 | | Q7CLU6 | Q5UB51 | Q9H252 | | Q1B9D5 | |
| C3MZC0 | Q4AEH8 | | A6WR28 | | Q9CN88 | | B1KDX3 | P0AEQ7 | A8L588 | | Q0SNB4 | |
| Q5HS97 | B0RYP5 | | P32587 | | A2S8D4 | | Q90Z04 | Q9UB37 | P37567 | | Q6DPT6 | |
| A0KRK5 | Q5HDV7 | | Q9MUU6 | | Q3YT97 | | Q07951 | P37685 | B1IWY2 | | A8HTY4 | |
| P43866 | Q8BTU7 | | E0TXE1 | | Q5GT39 | | A0L1S6 | Q2P8V2 | Q03MQ8 | | Q2NIG9 | |
| Q74FG0 | Q98QM2 | | Q5X1H6 | | Q94CD8 | | Q54LV7 | Q61184 | A1R0M2 | | Q6MAB4 | |
| A1WXK9 | B1AIC4 | | P0C988 | | Q3JMQ7 | | P22715 | Q6FZB7 | C3LED0 | | A8AYG0 | |
| O49250 | P30951 | | Q7VKE5 | | P50511 | | P0A2D0 | Q63T14 | Q7MPI7 | | Q11NN8 | |
| A5IBL8 | P11121 | | P35720 | | B7HJ43 | | Q7XRU4 | P49495 | Q2S3N1 | | Q53W83 | |
| B0VU92 | B1KMX1 | | P40690 | | A0QRN3 | | C5FRS7 | B7IHU6 | Q5PH15 | | Q46XW5 | |
| Q80IU6 | P43242 | | Q9Y6G3 | | A8Z3Z0 | | P09907 | B2K770 | Q0I136 | | Q7NW95 | |
| O84033 | Q46IM3 | | Q8RPQ1 | | Q97SA9 | | Q1CLW0 | P10666 | Q2IH01 | | B8DG13 | |
| Q5E232 | A3CNE7 | | P77399 | | Q980K0 | | Q54FE8 | O67637 | Q8ZLZ9 | | Q865P3 | |
| E9Q6Z5 | Q9JUD5 | | Q65KT8 | | A7H515 | | P85411 | Q1JJ57 | Q8VEL2 | | O15446 | |
| P53577 | Q9JVH6 | | Q32ZH7 | | Q9Z716 | | A0PJT0 | B3PNC7 | A7FNI2 | | P34968 | |
| Q9Z320 | A7X574 | | Q5RB63 | | B0S3Z1 | | B9M170 | O98947 | A9W4T0 | | Q53GA4 | |
| Q2HDP2 | B7VBN2 | | A4G4T3 | | A1A5I1 | | B2A3G6 | O25687 | B9EBF6 | | P69212 | |
| A0M2K0 | P52951 | | A2SSV3 | | Q98664 | | Q5R4S3 | Q02WD3 | Q492D3 | | Q5ZJU3 | |
| B2SFD5 | A7FG49 | | O60503 | | A8YUR4 | | Q931Q6 | Q1MMV7 | C1A1V2 | | Q6D3B9 | |
| Q327W0 | P21031 | | Q80IU7 | | A0A390 | | P27119 | Q8RIL0 | Q0AUL1 | | Q9ZHV9 | |
| Q5L627 | P50615 | | A3CM09 | | Q97KH9 | | B4SP12 | A1RIW8 | A0KHG0 | | Q3ZYG8 | |
| C5M545 | A9M3T9 | | Q06277 | | Q1LI27 | | B0SA32 | A2QWU9 | Q7WEL4 | | Q5QUB6 | |
| A8YYT8 | B4SC70 | | A0L4Y9 | | Q1H4U4 | | C1DH55 | O22020 | Q1CTA2 | | P9WHA8 | |
| A3MIA9 | Q914N0 | | Q6CIB3 | | B1MGC7 | | B2S303 | Q1KLY4 | A9KEF0 | | O05161 | |
| A8FYW7 | B8CND4 | | P66899 | | B2UEK7 | | O32210 | P0CZ66 | A6QQW8 | | Q87DH0 | |
| Q1RF89 | Q03YE3 | | P50435 | | Q03ZP6 | | P61631 | Q215E8 | Q1GB31 | | Q72ZG3 | |
| Q755A7 | B3WE58 | | B3GXZ2 | | Q2TZQ9 | | C3N5R8 | Q0I7L8 | A4TNE5 | | A4IIK1 | |
| Q87RN0 | A6U3F7 | | A4XYY3 | | O26519 | | Q07NL6 | A0KWF4 | B3QLK3 | | Q6NY74 | |
| O98945 | Q32E99 | | Q2LUA0 | | P80036 | | Q8EWX0 | Q68RW6 | Q7A3E5 | | Q62JK8 | |
| B1KMY1 | B8IT21 | | C4K1P2 | | Q08432 | | Q8NSZ2 | Q8EUC8 | B5RD49 | | Q14392 | |
| B4TAX3 | B2U278 | | Q8QFQ4 | | B5Y988 | | P0A8I9 | A5GMM6 | Q7V992 | | A8AMA5 | |
| Q87VF4 | Q3M644 | | Q65RR6 | | Q89JA0 | | Q03UL2 | P12858 | Q6GDD8 | | Q32B31 | |
| Q8U3M7 | Q4FP52 | | Q03114 | | B6ISX8 | | Q48SI7 | B0UWR4 | Q1XDI0 | | P16892 | |
| P35267 | Q9MUU3 | | Q31H20 | | B3QZG8 | | A1KUQ3 | Q43125 | Q31UQ7 | | Q8VZZ4 | |
| Q197F8 | B1K0S6 | | Q5HC38 | | P68436 | | P32010 | B0K9L7 | P51083 | | P03398 | |
| Q7YM14 | B4U161 | | Q2Y636 | | Q8K209 | | Q4QMG1 | P51188 | Q759Q6 | | Q68RW7 | |
| Q9FPD5 | Q7TST0 | | A4QNL8 | | A4G0D0 | | Q6BLH9 | Q8K2Y0 | Q5HX30 | | B4TZR1 | |
| B6YQ80 | P0A731 | | Q01581 | | O01802 | | B5ZC77 | B9E1F8 | B2RSH2 | | Q82VN1 | |
| Q3J9B1 | C1CIU0 | | Q6L147 | | Q4L8A6 | | P71479 | A5IHQ9 | C0ZA70 | | B8HY03 | |
| Q8H1Q8 | A1RU26 | | C0M9C4 | | Q2NL14 | | Q9X6Y9 | P05036 | Q8TU90 | | Q5L172 | |
| Q9KUI0 | P47768 | | B3DSB4 | | Q8DKU5 | | Q8ZTJ2 | Q7A085 | Q9HI36 | | P0A091 | |
| P06583 | Q8GBD4 | | P67876 | | Q9BH04 | | B7NP10 | A5FGN1 | B4XMC6 | | A9L9D7 | |
| Q62GL5 | Q5FVX7 | | C5FGB1 | | Q6PIL6 | | A6T5I9 | O65621 | Q6CZY2 | | P83583 | |
| A4VI28 | Q1BAU9 | | Q5RDF9 | | Q2FW13 | | P26410 | Q7RJF9 | A3QB25 | | Q7F9K4 | |
| Q2NFF7 | Q4ZUN7 | | P69418 | | C1D9Q4 | | Q5N3B7 | P9WGB2 | Q9PB76 | | O31298 | |
| A6MMM9 | Q8HXN9 | | P61930 | | Q8EK69 | | P08075 | A5U9I2 | Q8K2D6 | | A7MIC7 | |
| B7J0N2 | Q9DA75 | | Q8SWM6 | | A5IC29 | | A7ZEZ5 | Q96GE6 | O07084 | | Q113I7 | |
| Q5ZXU1 | O44578 | | P80074 | | A2BZ43 | | Q7WRC7 | Q6MG22 | A3DJ11 | | P14788 | |
| A1V713 | B5YDV5 | | Q9WU45 | | Q50167 | | A0RQ16 | B5XUY0 | Q2NRS4 | | B3QP02 | |
| Q5R143 | Q04R63 | | P36156 | | Q5Q0T9 | | Q4A701 | Q2HJ96 | Q5NRP8 | | Q759K4 | |
| B5F072 | A9H3R6 | | A6QJ80 | | O27957 | | P59843 | A6U5J1 | P0A4E3 | | Q9JII1 | |
| Q62838 | Q17Z25 | | Q252Q4 | | A0KLY1 | | A0RP87 | Q91ZK7 | P56997 | | P45514 | |
| B2JZ23 | A4YD26 | | Q5U2Q7 | | A8GYV7 | | B9JLF7 | Q9FV70 | B8DFZ6 | | P66280 | |
| A0LCY8 | A4QKI4 | | A4WKK8 | | Q8N1P7 | | Q4P5V5 | P04757 | Q3Z611 | | Q601J0 | |
| Q0P3X8 | Q8D2W5 | | O59932 | | Q0I6T4 | | B5Z225 | Q804A5 | Q58742 | | Q21SW6 | |
| C4XRE2 | Q0VZ41 | | Q9STL5 | | B8ERW8 | | Q8IZN7 | P52434 | Q0HXV7 | | Q97ST6 | |
| Q9UIV1 | Q54F87 | | O66891 | | Q9GL72 | | A0T0K4 | Q748I9 | A8ESU5 | | B7NI94 | |
| Q2KU21 | Q57H68 | | Q493I3 | | Q6NLA5 | | Q5LNJ2 | Q07RN1 | Q6AQV6 | | Q1WT99 | |
| Q1GCT5 | A2SSW7 | | P0AFW5 | | Q81M50 | | A1V9X6 | Q05994 | B2I8H6 | | Q9W328 | |
| Q64VX4 | B1H069 | | P97536 | | A5GJ90 | | A5EGE2 | P28023 | O02733 | | P27646 | |
| Q5XAX5 | A4SRW9 | | A4YAL0 | | Q1RI55 | | C1CRK5 | Q6HEW2 | Q6FJ70 | | B2JLS3 | |
| Q1CJE5 | B1XIT5 | | Q90YU9 | | B7J0E6 | | A9R910 | B4SRK8 | P04352 | | P75104 | |
| B3QVA0 | A8A6N0 | | P67523 | | Q1QMB9 | | B6YT17 | B9DSZ6 | Q5YRP8 | | B4UBA0 | |
| Q9BBN1 | Q7RTV0 | | B2V5A9 | | Q4PS85 | | A1KU21 | A0KGL0 | B1Y901 | | P68256 | |
| Q8FQY0 | Q182T5 | | A5GPD4 | | Q4ZMA9 | | A6QCI9 | C1DAT3 | Q87Q53 | | Q9CAP8 | |
| A4RHR8 | Q9FUL4 | | Q05186 | | O88483 | | A7NN50 | Q881U0 | Q6G1G7 | | Q58539 | |
| Q7WJ93 | Q1BH71 | | Q5HQ70 | | B5R6B4 | | C1L0I8 | A9VY77 | P18600 | | P60853 | |
| P82471 | B7MNZ6 | | B1Z9C8 | | B8GV59 | | Q9I587 | Q17QB7 | B0SEI3 | | Q839V4 | |
| Q47840 | P72800 | | Q8G4H4 | | Q9I1W2 | | A3PI66 | A3MRU9 | Q28469 | | P0A2A2 | |
| A1SDH8 | Q9A715 | | B0G101 | | Q3BXI0 | | A6S3R7 | Q2A1Z9 | Q1CSG4 | | Q17IE8 | |
| Q9LSD8 | B7KBH7 | | B4T2B2 | | B5YXR6 | | A8Z213 | P54382 | Q5LMQ4 | | Q4AA74 | |
| B5BGL0 | Q9LXM8 | | B7MNM2 | | Q2G636 | | Q0AAV2 | P40518 | Q2FSE6 | | B6EPT0 | |
| Q7VQM3 | P81693 | | Q91FN1 | | Q9YFV2 | | Q757C7 | B0TYD1 | Q9HJW8 | | Q5PQN7 | |
| B0V8J2 | P15164 | | Q8MUC1 | | Q6LVI5 | | Q8ZBL9 | Q93QC6 | Q0SI56 | | Q9ZBY8 | |
| P16356 | Q7VR10 | | Q7TMK9 | | P77712 | | P60983 | A1DDV0 | Q2FNT7 | | Q99252 | |
| P21010 | Q7U8T6 | | Q1MGQ9 | | A7ZLX3 | | Q9TLY9 | Q9SKJ5 | Q1GHF1 | | Q9Y217 | |
| Q9XJ38 | A6UXT7 | | Q4R703 | | Q92J87 | | Q7ZVZ7 | A7ZTI7 | A0L1P2 | | A6Q228 | |
| Q6W4T3 | Q9KCX2 | | Q8FUI5 | | Q93V47 | | P36320 | P81392 | P09765 | | Q8P2D3 | |
| C7GW39 | Q6D5H7 | | B8GXB6 | | B5R2J0 | | A8G9S7 | B3QZG6 | Q9V9Z1 | | B2XWQ6 | |
| Q40577 | Q9FV49 | | C5MA32 | | C4L012 | | P15746 | A8M8T7 | A1KAV5 | | Q0TTB0 | |
| P31617 | Q2YU75 | | Q5ZCW1 | | Q73VH7 | | P0CE01 | A3BMZ5 | B3QZT9 | | P69220 | |
| A3MWI4 | B9J8D1 | | B8DFR4 | | A2QA23 | | A5ESV8 | B8I808 | C0QRH2 | | Q3SZW3 | |
| B0TI68 | Q058E6 | | Q5P7M2 | | Q2FEU3 | | B5DGM7 | Q6IND7 | B3RFR8 | | Q668N7 | |
| Q92N33 | Q46WY4 | | Q8CG09 | | Q08324 | | B4SHW1 | A1SNL9 | A6TWI3 | | O27125 | |
| O26144 | B5ZB18 | | Q8D4H4 | | Q0W4B8 | | Q8WXA8 | F4KFT6 | B2FUB6 | | Q86VH4 | |
| Q17905 | P49383 | | P23023 | | Q3J4H6 | | Q67171 | B7GS74 | P28566 | | P29410 | |
| P51210 | Q5HAT0 | | Q80TQ5 | | P62399 | | O75844 | B5YKS2 | A4THE9 | | B4RCC0 | |
| Q6PPB3 | Q6D093 | | A1VEG0 | | A7X1Q8 | | Q6R648 | Q5AL03 | Q84N38 | | E1RHM5 | |
| B7NFS6 | Q8XM22 | | A4IHY6 | | Q1R073 | | Q9CNY5 | P0A5W3 | Q4A8J6 | | Q08BN9 | |
| Q3KHA2 | Q9NZA1 | | Q06FM7 | | C1CTY2 | | A5CRX2 | Q9Z7M5 | Q4X1Q4 | | Q38919 | |
| Q61139 | P15286 | | Q6F6K2 | | O77715 | | Q9NZF1 | Q5ZJU2 | Q8R4N0 | | A7FLQ4 | |
| A9IVB9 | Q5X4M9 | | A7ZAE3 | | Q8RKT5 | | A9ADK3 | Q8V571 | P79237 | | A4QL65 | |
| Q8D3B7 | Q6C6V8 | | Q0BC21 | | A4G4P0 | | P22361 | B2CNY4 | P11132 | | Q8TT44 | |
| A1UU39 | A4W5R2 | | A9ABS7 | | A9A135 | | Q3KFI7 | Q6CDS6 | P92847 | | Q57648 | |
| F5CPE3 | A0LSV2 | | Q5R1S9 | | Q5PBG4 | | Q2U8R6 | Q87SA4 | P82751 | | A4XL78 | |
| Q9GYI4 | P35176 | | Q6VN20 | | Q0HNV7 | | A1CZE5 | Q8GLE9 | A2RG32 | | P58925 | |
| A1R0K9 | B0TI64 | | P69757 | | Q9BIW5 | | Q49X49 | A9ADJ6 | A0L5Z6 | | B0KSA0 | |
| Q27783 | B8G6X2 | | A5GR53 | | Q0SYP0 | | Q46398 | A0B6U3 | Q24JY1 | | B4S7L8 | |
| Q2PMQ0 | A2S451 | | B5ETZ2 | | Q6D2N5 | | A5D172 | Q3SZC0 | B6EIX9 | | B1XB40 | |
| Q1R5D9 | Q5GWW0 | | B6HZ68 | | Q93HD3 | | Q2FHQ4 | A5ISE3 | Q8YRN1 | | P42893 | |
| A4Y5S0 | P43771 | | Q8TQQ1 | | C0JAR0 | | Q1Q986 | Q5WZI6 | A7IPK6 | | P55293 | |
| B8DBG4 | A7HVD8 | | Q9DG99 | | Q74MI6 | | A3QAX7 | C3ZAH2 | A8GKT5 | | P93746 | |
| C1AFZ7 | F5ZTT9 | | A7HT11 | | O78256 | | Q73XM4 | B0SFU6 | P9WPN7 | | A6UN87 | |
| A0T0X6 | P61021 | | B0KL32 | | Q8HEC0 | | A4VGT8 | A6ZQG7 | Q2FI05 | | B5EAS8 | |
| B0KQA1 | B4T0Y6 | | A3M1G2 | | P0A2R5 | | A8Z1Q1 | Q0IMG5 | Q8EHU2 | | P10064 | |
| Q24320 | B1J3K5 | | Q9ZJQ9 | | A6UDV3 | | B4RNP4 | Q89HW8 | B7L656 | | Q2V354 | |
| B7K221 | B2IJ38 | | P17425 | | A1RSN2 | | Q7NQF7 | Q8RAE4 | Q0B711 | | B0KN18 | |
| A4XZA0 | Q1R175 | | Q9WVG1 | | Q1XDJ9 | | P00325 | A3QJR4 | P31419 | | P05394 | |
| P9WP71 | A8F6X2 | | Q54MV2 | | A8F005 | | A2BY56 | Q2VBN9 | P0CD60 | | Q0T203 | |
| Q65JJ6 | Q2RNH9 | | Q19143 | | A2R4I6 | | P68264 | Q5Z061 | A5DUB2 | | Q9HNR3 | |
| C5PHM7 | Q24QL6 | | O08543 | | A3Q0V0 | | Q9KPB3 | A8H1E0 | Q8NY77 | | P51752 | |
| Q3Z7F5 | A6VX99 | | B9EKX1 | | Q5R482 | | B3R3N0 | Q27956 | Q8ERU6 | | D4AXX2 | |
| B2TXU1 | P59117 | | Q920A6 | | Q9H0K6 | | Q4QMV7 | Q2YNB9 | Q8K9F3 | | Q8XHR9 | |
| O04403 | B1M787 | | B8F5T4 | | Q12968 | | B0VN04 | P07634 | Q28603 | | Q9PKW4 | |
| A1E9M9 | Q31H51 | | Q4FLM3 | | A8F4X1 | | Q8R2J9 | P25063 | Q9CAL8 | | Q9ZS40 | |
| Q09FX2 | B7MTE3 | | Q3YYN2 | | O78928 | | P25671 | Q1C367 | Q1MQQ0 | | Q80ZM8 | |
| B0BC02 | Q95IT3 | | B1XAT9 | | Q12481 | | A1KFE4 | Q820D6 | P63978 | | P0CR21 | |
| Q1JJB0 | C3KZ49 | | B8I5V9 | | Q2YNC6 | | Q72XA8 | A5VQZ1 | P0CW06 | | Q9RRU5 | |
| B1I814 | Q9ZDX3 | | Q08269 | | P0C438 | | B9E3M4 | C3PP97 | C1GG27 | | P26849 | |
| Q9Y946 | B1HQG9 | | A8AC01 | | P20908 | | Q5NRQ1 | Q68RX1 | A5U766 | | B2KE25 | |
| Q4USG1 | B3PI96 | | C0Q6K4 | | Q62931 | | A0T0Z6 | Q1J6M7 | Q88XV9 | | Q3IEE3 | |
| Q6MG84 | Q0VCP1 | | Q59XQ1 | | A8GYB4 | | Q8WHY3 | Q68W74 | P41102 | | A0JZ49 | |
| Q1QZ10 | A1V2G0 | | B2TTK5 | | P84290 | | Q054E1 | P27402 | Q1ISB5 | | B7JJY0 | |
| Q324Q5 | Q9CL42 | | B5F1Z5 | | Q1AVH5 | | O43303 | Q9UN86 | A5D2F9 | | P94664 | |
| B0Z4V3 | P0A1Z7 | | Q50DM8 | | Q6W5G4 | | Q95M54 | Q8P3X4 | P56398 | | A7H3U1 | |
| Q1H4R3 | Q02G12 | | Q06011 | | Q1MSD5 | | P10394 | P12796 | C5DJH5 | | Q46WC7 | |
| P01091 | C4ZA73 | | Q0BLC5 | | Q4UTX4 | | Q6NCY4 | Q4R8L2 | A6VFY5 | | Q8QMQ2 | |
| Q07MT9 | Q39XY1 | | Q09232 | | Q47JS8 | | Q33BZ0 | Q88VE2 | Q8ZC45 | | P13783 | |
| Q3K119 | Q66209 | | Q9X7F0 | | Q5E8A3 | | B7VLD2 | Q5A1E3 | Q9N5K2 | | B5YWE1 | |
| A0AIC1 | B5XK61 | | O51601 | | Q91G70 | | B3TN90 | Q5RFN2 | Q96MB7 | | Q5RD26 | |
| Q2V4C4 | B3PBD6 | | P03875 | | Q0BIH5 | | A1UE95 | A6NJB7 | Q6FJ31 | | A5IYX4 | |
| Q7W2E0 | Q7A115 | | C4L3C2 | | P65610 | | B0BQT2 | Q9JIL4 | Q38XI2 | | P32439 | |
| P38264 | B4TDZ4 | | P9WNA6 | | P56157 | | C1CQB5 | O83644 | Q39Y17 | | B2Y1V6 | |
| A4SJ79 | A4IFD8 | | Q9CNL5 | | P66152 | | Q9RR81 | B5FP38 | O01360 | | Q046D1 | |
| P06360 | A1KBL9 | | P04656 | | B7MFJ7 | | Q5V2X7 | Q9CA58 | P39635 | | A1TSK5 | |
| Q837C8 | Q65DF9 | | A7MKY0 | | Q50173 | | A6U2W8 | P55264 | Q8P8Z8 | | O67085 | |
| O31778 | Q1GND0 | | Q8FL64 | | Q17QQ1 | | B3EEM7 | P23755 | Q3KLI4 | | Q96G03 | |
| P83114 | P0DE00 | | Q252W4 | | B5BB85 | | Q67JT2 | B9DSV7 | Q4WWN2 | | A1KLN6 | |
| B4TFA5 | B8CWJ6 | | A1S8R3 | | A2QUS7 | | Q3IRM5 | B1LW29 | Q1IJS7 | | A8GFH6 | |
| Q7WI90 | Q8FFV5 | | Q6CRH2 | | B0CAD1 | | A6LSF2 | Q0CSL5 | B8CHM9 | | A8GVD2 | |
| P23580 | Q8NEV9 | | P20807 | | A1SZY2 | | A2RF12 | Q8GP72 | B3E7S9 | | A0KPW7 | |
| P07192 | A2C5I6 | | Q9JHF9 | | Q6D201 | | P79820 | B3QRP3 | P9WGB5 | | B2HZM0 | |
| Q9BE45 | Q2WGF1 | | A5FZ26 | | O18924 | | Q55C62 | P50609 | A3Q972 | | A6T469 | |
| Q5HAN0 | P05154 | | C0PZX5 | | P0CAZ4 | | Q7VLX6 | Q9MRF3 | B4SLI5 | | B0CKX7 | |
| C3LUM7 | B7I3K0 | | Q62GR7 | | A8A271 | | A8FEC9 | Q54GD0 | P05079 | | P68864 | |
| Q2MID8 | Q7CNP4 | | E6MUA0 | | P35224 | | A9BCH4 | A0JZ27 | Q7U4P7 | | B0K5P2 | |
| A7GJ50 | Q08080 | | P47785 | | Q8KRD3 | | Q11QB5 | B5R757 | Q81Q29 | | C3NHQ5 | |
| Q96LS8 | Q61907 | | C1CP89 | | P29249 | | A1SW94 | C3MAG2 | P44722 | | B2RZI6 | |
| Q5SD19 | Q60887 | | B5R486 | | Q0I0X7 | | Q196Y8 | Q6PHN9 | Q724G3 | | Q0BIB8 | |
| B4S3A5 | Q89A67 | | B2I5X3 | | Q7U8F2 | | O54890 | P79990 | Q5HID5 | | Q62HC0 | |
| A6TC99 | Q669P7 | | A5F696 | | Q2A1I2 | | Q9PGB5 | Q9FWS4 | Q8RDA2 | | Q0T3D7 | |
| B0Z5I4 | B2U2T9 | | P31315 | | P59954 | | Q6DN52 | A9M838 | B2VGX3 | | Q2FK43 | |
| Q9Y5H8 | Q02955 | | Q95719 | | Q99J95 | | P25238 | Q3M4Z2 | B2S4I8 | | Q49W40 | |
| Q6LN85 | B0Z4M9 | | Q6FV12 | | C1ET55 | | Q06FP4 | B7N8W8 | B4TQN8 | | Q8CXM7 | |
| Q9TLT9 | A3NSC3 | | Q3BV71 | | Q5XI97 | | A1S382 | A9R1G4 | P9WPT3 | | Q9RY49 | |
| B4EET6 | Q4FLL3 | | Q5HRL0 | | P16405 | | Q1JEB8 | B4SRY7 | C3MZY6 | | C4K3I5 | |
| B7LXT6 | B9E8Q8 | | P9WQL9 | | A6UT21 | | Q8U0W9 | P0A6Q3 | A3PGM6 | | Q95153 | |
| Q48VS4 | Q5N3C1 | | B0XXS1 | | B1YAF1 | | A4T1U0 | A3M2W0 | Q252W5 | | P02395 | |
| O07584 | Q9CNN9 | | Q18J48 | | Q5J6J0 | | Q60CR5 | Q1J7B6 | Q2MY51 | | B1II75 | |
| Q1WSM8 | Q3MAV9 | | Q32EZ9 | | B0K0M0 | | P43724 | Q7U3V0 | Q1RFB2 | | Q8UI70 | |
| P66327 | A5GWP0 | | P82627 | | A6VA50 | | Q2NIV1 | P16430 | P22771 | | B2KEN2 | |
| B7MTC0 | B5XNJ9 | | P34985 | | A0PX93 | | Q4UK23 | O57899 | Q9UX83 | | P40450 | |
| B0SNK3 | A4WG55 | | A8FP09 | | Q6P4L6 | | Q7WFS3 | P9WK05 | Q5EA18 | | A2C4N6 | |
| P04813 | Q3IY22 | | C3LQC2 | | B1J2A2 | | Q9TLT8 | E3VWI6 | Q7UA42 | | Q8L518 | |
| Q1CDL3 | B2FN86 | | Q9JZA5 | | A5UDJ4 | | Q5HNH4 | B6K6L6 | Q06032 | | B1NWH3 | |
| Q3UE17 | A1S601 | | P47673 | | O47685 | | A7ZJM7 | B1LJN4 | Q3JMQ3 | | Q74ZS6 | |
| Q6H9K5 | A2C873 | | P49496 | | Q4FUE6 | | Q2A783 | Q9XJR5 | Q9CEJ1 | | Q5L4P3 | |
| Q7YJU1 | Q89HR3 | | B7MQD0 | | Q06GL9 | | A1S8R2 | A5W7B0 | A9IJN0 | | P07307 | |
| A2SK12 | Q0VN37 | | Q09FY9 | | Q2YXD1 | | P39196 | Q51792 | A3LWH3 | | P18606 | |
| P48136 | B4T9M8 | | A7TLU2 | | Q9RTG5 | | Q9PPS3 | Q51366 | Q2TW49 | | Q6K5X1 | |
| P26250 | B1VKH6 | | Q05537 | | Q2SDW8 | | Q6PF69 | B5Z0D4 | O15541 | | Q9K8F1 | |
| P9WNW5 | P80366 | | Q03ZL9 | | B1LH07 | | B5Z8J8 | Q97IQ6 | O47554 | | P0AB96 | |
| C6E2L2 | F5CPD7 | | Q7XR61 | | Q04G74 | | B3H2J1 | Q3A129 | B5XLG8 | | Q00080 | |
| Q8MKG9 | Q99S48 | | B2RLV6 | | Q87WY6 | | Q57J48 | P65987 | Q5TM50 | | A7ZDQ5 | |
| Q7VNP1 | P16380 | | Q5M249 | | C0RE70 | | Q588U8 | Q84KK5 | P83436 | | A5UDT7 | |
| Q0J4P2 | B1YIR9 | | Q2PBM3 | | Q1J5S6 | | Q5XA13 | B1J0X8 | A1TYK9 | | A2C3L3 | |
| B1IPZ4 | P23259 | | Q9JYH8 | | B5FCZ2 | | O55135 | Q5ZJ56 | P08219 | | Q47VT1 | |
| Q738L2 | Q9AAA6 | | P0CQ80 | | B7MR71 | | A7MY83 | B9LBM0 | C3LWV8 | | Q63068 | |
| Q6FAM6 | B3GZ10 | | Q0VFE7 | | A6TS50 | | C0ME18 | P0A6I4 | C1CDU1 | | Q55029 | |
| B9K7W0 | P09914 | | P53050 | | Q250K6 | | B5EZK7 | P09513 | Q1X711 | | Q1KVU9 | |
| O00591 | A7H2U1 | | Q211F8 | | Q69140 | | Q2FEZ4 | P14681 | Q661I0 | | Q8MJD0 | |
| A9WUU3 | P9WI60 | | A3D7P9 | | Q47836 | | Q0AAD7 | P50512 | Q6NUJ5 | | A4ITK4 | |
| O83873 | Q5N453 | | A4W8T1 | | Q9M3I4 | | Q5AHC2 | Q0T847 | A0R946 | | B7GM52 | |
| A3DJK3 | Q8W2F1 | | B2AH57 | | P18106 | | Q9CE39 | Q73L91 | Q3U1G5 | | Q5M2R5 | |
| Q7CMM8 | P05228 | | P24824 | | Q67ER4 | | Q1J5H5 | A4QP81 | O68579 | | Q5VKP0 | |
| Q68EY2 | Q42908 | | B2VCK3 | | F4G4E2 | | Q8C0J2 | Q85AW7 | B2UBN8 | | P18165 | |
| P18905 | Q11C97 | | Q0A6K2 | | A1T8U7 | | P68555 | Q5VUY2 | Q8RF14 | | C0MBS9 | |
| Q7G8Y3 | Q9VTE5 | | B1LY47 | | A1SZY7 | | B1XT10 | Q5AA40 | P0DC32 | | A9M5N8 | |
| P0CZ00 | C3MZR9 | | P61988 | | Q3K3W6 | | Q9JX59 | Q1RF97 | Q8DY92 | | Q0MQD2 | |
| Q96UL8 | Q6BGY8 | | Q1JCJ9 | | B7NEU0 | | A6LPU0 | Q5LHL2 | Q9F7Q9 | | O74884 | |
| Q0A446 | A7X5V7 | | Q8D232 | | B6DZD0 | | P84297 | Q92J29 | B0B7Q0 | | B3PKN1 | |
| P15701 | P07267 | | B2U7R0 | | Q8R1Q3 | | P82891 | Q8XID0 | A1SNN6 | | Q9Z313 | |
| B1ICL9 | Q9LKN0 | | C6DZ58 | | A5N5W0 | | Q28DV7 | Q4ZWS5 | Q99797 | | P64181 | |
| Q7NBF8 | C5DZT2 | | Q0TMS7 | | Q5BDU4 | | A9L0P6 | Q197D8 | B3TN82 | | Q9ZCX4 | |
| Q4P3U9 | P37112 | | Q2YD11 | | P52712 | | Q8P999 | Q6GD66 | Q03683 | | P03612 | |
| A5GF86 | B1AIL9 | | O68128 | | Q5SHF5 | | A3PDJ8 | P37276 | Q66405 | | Q24F59 | |
| Q83G55 | Q9SMI3 | | Q57826 | | P40023 | | A8A683 | Q48GW4 | B7NQ13 | | A8FKN5 | |
| Q39Q55 | P48040 | | Q9RUP2 | | A4IGL7 | | B5XZ17 | B0TX39 | A8LEP8 | | B0BRX7 | |
| C4Y4A0 | C0PZJ7 | | A8WCC4 | | A9W6T5 | | B1MWX1 | P46749 | P30956 | | P47711 | |
| A0PUG0 | B1J0Z5 | | P22637 | | P08223 | | A4SVE2 | Q6A9W5 | P0DE60 | | Q0SM52 | |
| Q5V5F7 | B2K6T3 | | P83297 | | Q12ZB3 | | Q9H0A8 | A4XLK1 | B2HGE6 | | Q8F3J4 | |
| A5GUA8 | P44624 | | Q9Y802 | | Q7XP59 | | Q8Y666 | Q970Z2 | P12916 | | P0A803 | |
| Q3SF18 | Q8E6H6 | | B2K6M5 | | O16452 | | Q9PE17 | Q05758 | P37374 | | Q4L3K3 | |
| Q5R7L3 | B2HSN0 | | O05467 | | P0DA61 | | P80246 | Q0AQB0 | Q9SWB6 | | B1JUW8 | |
| Q12PZ5 | Q1GB50 | | P37810 | | Q9NYR9 | | O67265 | A6VL62 | O24785 | | Q9HV53 | |
| A5IWA1 | O16294 | | P24226 | | B9MBK7 | | O50312 | B4TSP0 | B1I9W1 | | P9WI50 | |
| O32956 | Q8XZI8 | | Q2L2F7 | | Q6LNV7 | | Q3ZIZ0 | Q9LFB8 | Q9I423 | | B9EJV3 | |
| A4QKI2 | P63251 | | B2S9H9 | | P64112 | | O13963 | Q5L687 | P17329 | | P19170 | |
| Q10980 | Q1LQM3 | | Q2V371 | | Q8SSJ5 | | Q2UL89 | Q93GF1 | A5FUL3 | | A2Y040 | |
| A9KL10 | Q3J5E0 | | P42157 | | Q7NAV1 | | Q2UGQ8 | P68031 | P56760 | | P08170 | |
| Q5GXY3 | O27077 | | Q7HHY5 | | A5IHQ2 | | B6IMQ3 | Q7MHB1 | C1CH67 | | A8Z3D6 | |
| C0QQ90 | Q7VZ81 | | Q93GF2 | | C1CGA5 | | B5QV13 | Q0TJ67 | Q9ZZE3 | | Q39659 | |
| Q8KAH2 | A1V8A8 | | A3DB95 | | Q31HZ6 | | A6UBQ4 | P0DC39 | B2I730 | | P30586 | |
| B8D067 | Q3A398 | | Q1GK21 | | Q8K9R9 | | P9WQH4 | B2VF49 | C1DSS8 | | A5IYM9 | |
| Q1Q8Y4 | Q17X66 | | Q7M461 | | Q91VC0 | | P02100 | Q0ACA3 | P25266 | | Q11QD9 | |
| B8I6H3 | Q35000 | | O94320 | | O97581 | | A0KQZ9 | P60887 | Q9HV43 | | A8Z454 | |
| P37141 | A2SLF7 | | Q08078 | | Q7XT07 | | P24403 | B9WED1 | Q54KI3 | | B1J596 | |
| Q57HA5 | Q8CCS2 | | Q5PFD8 | | B1XT00 | | Q9UT83 | B8CPE2 | Q9SMZ4 | | A8AA22 | |
| P69358 | C6A2E4 | | F1CIY9 | | Q75DB8 | | A5U9X6 | B4R8T3 | Q8ER27 | | P02985 | |
| Q9SKZ2 | Q1IXA0 | | Q0J3D9 | | A9A3J7 | | Q7NT75 | A5FMY8 | Q5M8L8 | | P0CC00 | |
| B1JU10 | Q97BW3 | | Q96C23 | | Q88KM5 | | P09070 | P0C6P2 | A6MMX7 | | A0M599 | |
| Q3Z5A4 | A5K5K4 | | O35090 | | Q5ZJI6 | | Q2FHZ1 | Q2NFX0 | Q9CET2 | | Q6B8S1 | |
| Q7T3P1 | Q4PFI5 | | Q1MG28 | | O27939 | | Q9RRC1 | P31682 | P68944 | | Q00PI9 | |
| Q3UZ01 | C1CAM7 | | Q9I9M5 | | Q9Y7P0 | | Q6NV72 | B1IWZ0 | Q9ZQ74 | | P06814 | |
| Q9JW71 | Q5ZZS1 | | Q99081 | | Q91Z46 | | Q6AG59 | Q70RW3 | C3PMU4 | | Q6ENW9 | |
| P13861 | Q0HEK0 | | P61217 | | P13481 | | B4RQW3 | C0SDG9 | Q557H1 | | Q4WN99 | |
| Q7VRT2 | P70562 | | F5ZEI8 | | B1LBN9 | | A9N8H2 | Q8LEQ0 | Q9URY2 | | A0LV66 | |
| A7X2Y1 | D3ZW91 | | B8G2Q5 | | A9N8V8 | | Q8Z687 | Q8BHH8 | P46972 | | B7LKC7 | |
| Q8RED2 | A2SLE1 | | Q95DP2 | | B8D8D5 | | Q9BV20 | Q11TN7 | Q3KSS3 | | Q9CHG8 | |
| B5YYB9 | Q30YX3 | | P20615 | | A6YGC4 | | C0QKM9 | Q0HL88 | B8GWR1 | | Q12611 | |
| Q1MPQ0 | P0A5V5 | | P19091 | | O46200 | | Q5NXR0 | P10592 | A0L2T3 | | Q8DH26 | |
| B8HD01 | Q3T1I9 | | B1K0L8 | | Q88XY1 | | Q6KZP6 | Q3V4X2 | C0ZUX9 | | Q9JF82 | |
| Q9LXS1 | P43605 | | A2BTD6 | | B5Z079 | | P64535 | Q9J5A4 | P86855 | | P46529 | |
| O84465 | Q5JGM7 | | A5GHR9 | | Q3ABU1 | | C1FNL3 | Q2L150 | B8GSS3 | | Q9BRQ8 | |
| Q1GK19 | B3EMI3 | | Q9M9E0 | | A5W9N5 | | Q7VSV5 | B0K5G4 | O26710 | | Q9GKL8 | |
| Q5ZML1 | Q04C45 | | P0A172 | | A1KGI1 | | Q3ILP4 | A1SWW0 | Q3AL87 | | Q985M5 | |
| P07673 | Q8W1L6 | | B5BGX6 | | Q7S4G7 | | O02744 | Q82S08 | A7YY54 | | B0YPM0 | |
| Q9W1N6 | Q5XI14 | | Q8NK83 | | A2WY46 | | B0TI09 | Q8UF44 | P83124 | | B7M8Q3 | |
| Q32KX5 | Q3AC15 | | Q7PT10 | | Q756E1 | | Q7VYC6 | A5VYP1 | Q6T4R5 | | A0RQL9 | |
| Q2FN18 | Q641G4 | | Q3BAQ3 | | A9A9U9 | | B0Z4Q9 | Q3AMI3 | A1A5Q4 | | Q3KME9 | |
| A6MMQ4 | P06496 | | Q2JKN5 | | Q1RMP0 | | B1L550 | Q8PPY3 | A0KHC4 | | Q9PE76 | |
| Q08E13 | B0KT65 | | D2TPR5 | | P38167 | | Q9FKQ2 | Q9YEQ4 | C6DYS3 | | Q9JIF0 | |
| Q5M575 | B7IP82 | | Q1J7C3 | | Q01VB0 | | A1CMA3 | B8GBV5 | A5G5A2 | | Q8EPW8 | |
| B7MI07 | P20806 | | B2A5W1 | | P40469 | | Q2ICQ8 | Q8BM47 | Q03VY0 | | Q5JI42 | |
| Q2L942 | Q9MI38 | | Q2YKR3 | | Q99UJ7 | | B8GV42 | B4IMQ8 | Q5F5X2 | | Q8Y624 | |
| A9W685 | A9ADK5 | | Q91V41 | | Q8M9W5 | | B4S5B3 | A2YBX5 | Q0VCF9 | | Q3ZBF3 | |
| A7GKE4 | Q7PC71 | | Q885W8 | | Q86HX1 | | B5XYK9 | Q5RCA2 | Q6FJP1 | | P10996 | |
| Q8MSX2 | Q2JV83 | | P69630 | | Q5H1E4 | | C0MBF0 | Q8TT41 | Q1R8X4 | | A4T9W6 | |
| Q6GZM9 | P0AG50 | | P44561 | | B5QVW2 | | A9I6E7 | Q91Y97 | P51485 | | B8G4U8 | |
| P66063 | A7GK28 | | Q9S2N0 | | Q81U23 | | A1VBE9 | A7ZSJ7 | Q11128 | | A2BL68 | |
| A8KYU3 | B0KTY0 | | B9E8Q7 | | B5XL08 | | Q9PJB9 | B0JGZ3 | Q9H3D4 | | P01058 | |
| Q4QNS2 | B2VIN2 | | P79191 | | Q7ZYD9 | | C0MEA0 | C4KIJ1 | Q09865 | | B3QE25 | |
| A7GTP6 | A8EXA9 | | Q8H3C8 | | P61331 | | B9IWX4 | Q4ZMP4 | Q3ZBK7 | | Q07HZ5 | |
| Q9W739 | P61026 | | A1AC51 | | Q81JH0 | | A8P1W0 | P0A4Z5 | Q46WE2 | | B1YP15 | |
| Q470E0 | A3MUK4 | | Q0KCC0 | | Q92LB8 | | P07314 | A5G3J9 | Q1ZYQ8 | | Q9LQ36 | |
| Q6MSN6 | Q63TM5 | | Q6FZN3 | | B5RDQ6 | | A5GIR7 | Q11XX3 | Q15843 | | Q83AR8 | |
| Q9JRF6 | Q9GHR6 | | B0T8D1 | | Q82UL7 | | P10778 | O59221 | Q31FB1 | | Q9CD69 | |
| P36249 | Q3Z7T4 | | B4E8Y8 | | Q9H336 | | C3LRQ9 | A6TDG7 | P50886 | | Q7CQY0 | |
| Q30PQ4 | B0Z5G1 | | Q1GK67 | | B2GJ05 | | Q5AI58 | Q6PA90 | Q65UT5 | | B5XKV2 | |
| A9HS30 | B3PG60 | | B7GH35 | | Q9S6S2 | | B1XLJ1 | Q5NDM9 | Q9I0R2 | | B2UVF4 | |
| A7ZPA1 | Q02198 | | Q03KD6 | | O81769 | | Q51741 | Q65UW1 | A6MMP4 | | Q9KD71 | |
| A9VP72 | A3DDH1 | | Q9VU65 | | A1KSZ5 | | C6DG38 | Q49L12 | Q3IF27 | | Q3AIH3 | |
| Q9X7A0 | Q0BAU3 | | Q7NVY3 | | B8F6N9 | | B7VHL5 | Q6GQJ8 | P47786 | | O13113 | |
| P9WPT4 | Q9CWT2 | | P87117 | | Q6KZ23 | | Q08DB0 | Q1BV09 | O07643 | | B2VFX4 | |
| Q8SRS6 | B7I459 | | A4VIF7 | | A2SHP8 | | Q1JF94 | Q5R0C3 | P50094 | | Q48709 | |
| B7N3I0 | C5CEA8 | | Q63481 | | P07519 | | P68799 | Q1WUJ4 | Q8R0J1 | | A8MFD4 | |
| A6VQU9 | Q9B1K8 | | B0BNS1 | | P43257 | | Q317Y0 | Q30T61 | P59083 | | P07171 | |
| Q985A3 | A5EXX1 | | P0A908 | | A9M3V8 | | Q4VZL1 | Q9CQS3 | B7UPX4 | | P50465 | |
| B4TE50 | A4QL78 | | B0S3R4 | | A5EX69 | | Q9RC23 | A9MPU2 | P14092 | | B6YVG1 | |
| Q8RH46 | Q929A8 | | Q49Y85 | | Q9D103 | | Q04441 | B9KPU4 | Q8H6G8 | | Q39LF7 | |
| P29697 | Q9HF68 | | O27983 | | P0A6N5 | | P61303 | Q6G2W6 | P49185 | | Q41348 | |
| Q55638 | A6UYF6 | | P0C6K8 | | Q86KD1 | | Q54FA8 | Q7A937 | Q8EFW4 | | A9M9S3 | |
| Q3Z0N6 | Q80W71 | | Q0BE28 | | Q1AX09 | | B7GYD0 | A3CWL5 | Q9JMI0 | | B1IRC7 | |
| P46414 | Q9FLN4 | | Q82K95 | | Q9Y2L6 | | E5KIB9 | A8A4K7 | Q91FU5 | | P12898 | |
| Q667Z8 | Q70XW5 | | P10075 | | O55739 | | B2UBA4 | Q7VRG1 | P93526 | | Q4ZXC8 | |
| A8XJZ8 | C3NFR5 | | Q63H79 | | P98005 | | Q5WDX4 | B6YWH9 | C4LJ85 | | Q32Q52 | |
| Q04621 | C1CH85 | | Q6BPN4 | | A7X2A9 | | A5CX66 | Q9UKP3 | Q9SUB1 | | A5EX92 | |
| Q8Z5N9 | Q6W6L7 | | Q801X7 | | A6ZMM2 | | C9XUE4 | Q6Q7X3 | P16148 | | B9DMK9 | |
| A7GTE8 | A8AWM5 | | Q0T9P4 | | Q6HEY9 | | Q7VQN2 | Q55E93 | P93673 | | C0Q9W6 | |
| P0A068 | B8ZSC2 | | Q73FX6 | | B5R6R8 | | A9MSY7 | B0CJW1 | P38372 | | Q91MG4 | |
| Q0TE01 | O76819 | | Q1GTK0 | | C1F1C7 | | B1MZQ4 | Q9V113 | A4TL95 | | Q3Z7W1 | |
| Q2LPL3 | C0PXF6 | | B2ITP3 | | Q6MRX5 | | B4HFB7 | A5W9J3 | P64103 | | Q06R71 | |
| P42343 | B3QNZ0 | | Q6LZL8 | | Q12T99 | | Q1R824 | Q99MC0 | B0KND5 | | A9H3Q8 | |
| Q9XSE4 | Q5FPZ5 | | Q7SZQ4 | | A9IPU1 | | Q6B922 | Q9Y546 | O62786 | | P24640 | |
| A6VC58 | B3R2B6 | | Q029I5 | | Q9PM77 | | B0Y752 | P16853 | A0PU16 | | Q07L51 | |
| Q3KPW1 | Q5RCZ5 | | Q3SRH5 | | A1DNG2 | | Q7MAV0 | Q4USP9 | A9WSX9 | | P00789 | |
| O14313 | P60105 | | Q9FJK8 | | Q8ZTF9 | | Q9TS34 | Q61727 | P33863 | | Q75VA8 | |
| A6UP47 | Q31YL7 | | A8ALD5 | | B5EZW0 | | Q6CKB6 | Q8G478 | Q64385 | | P07732 | |
| A0KTP8 | Q890R1 | | Q0I7L9 | | A2SPM1 | | A3CR17 | Q5BJB7 | A8G9U7 | | B3EWR7 | |
| D4GU68 | B8D7C1 | | Q4UQB5 | | C6DHX7 | | B8AIK3 | Q2GL43 | Q80VM8 | | O55758 | |
| Q9PPU6 | B1J0J4 | | P49665 | | Q9KJC3 | | B1I1B4 | P41947 | Q15WB3 | | Q97SU3 | |
| C5DYK5 | Q2NWZ6 | | A6QHG3 | | B7GNK3 | | A6Q4Q9 | Q5HWL9 | Q8DFB2 | | P9WGZ2 | |
| A0AF64 | P03143 | | Q27520 | | Q7A184 | | B8H3C3 | P0DD14 | Q9UPN4 | | Q6FZE6 | |
| Q8AYC1 | P53332 | | Q7MLF2 | | A8GPD4 | | Q1WT14 | A8Z2N4 | Q1LX59 | | P0CD85 | |
| B7MD74 | Q3J9J6 | | P69778 | | Q9L1I0 | | Q55ED1 | A8Z497 | A4VPP0 | | E6UIS7 | |
| Q1QXY5 | Q19VA6 | | B2UV20 | | Q9ZUI3 | | Q0ITU1 | Q06704 | O53521 | | P0CH56 | |
| D2AJU0 | Q5E6L4 | | Q9HIH4 | | Q48F12 | | A9A5K6 | Q93YZ7 | Q9PE41 | | P15133 | |
| Q14IN9 | A0PM63 | | Q8K385 | | Q5FAG1 | | Q8X6X6 | A1A5G0 | Q2TBH1 | | Q9P7F2 | |
| Q0WQG8 | B7MQW3 | | P29321 | | Q9CQI6 | | Q6MSP5 | Q8VI04 | Q9TKX8 | | Q9LT17 | |
| Q7U477 | Q7TPQ9 | | P12737 | | C3L6X4 | | P0ABU7 | Q73HM7 | B2A474 | | P69546 | |
| B5F7G0 | A7TQM5 | | P10396 | | P58885 | | Q39DS5 | A4XST1 | Q1GWJ5 | | Q82VB9 | |
| P15443 | Q6GHN9 | | Q4VX62 | | B5YT56 | | B8DKV6 | Q92358 | Q255G8 | | P48700 | |
| Q9LU90 | A0Q5J0 | | C3P9E6 | | B6J258 | | Q9GZZ8 | A1JN60 | A9V767 | | P10365 | |
| P0CL03 | P16615 | | A8G7I0 | | Q2IL45 | | B9KSE2 | B3R2P9 | Q8P137 | | A4SJ60 | |
| C0SGX7 | Q2S4L6 | | Q3KKY9 | | P60893 | | P14575 | P15274 | C3P742 | | Q6NRD5 | |
| A4WBH1 | A1A4L5 | | Q03438 | | Q14CM0 | | Q9CRD0 | Q865F0 | O35116 | | P9WHK5 | |
| B5BKQ8 | Q9J3N7 | | C0Q8C5 | | Q88RW8 | | Q6KH82 | Q07KK6 | B4SCE7 | | Q1HGK7 | |
| Q6NSR3 | Q6ACB0 | | Q4V7T9 | | Q3JY54 | | A5E8A7 | P78990 | Q49176 | | Q65VH4 | |
| Q5RBB5 | B1KKX7 | | P01950 | | Q0HLM3 | | Q5L6U0 | Q18JI3 | Q8FVR1 | | Q6NCY0 | |
| C3PKQ1 | Q3KJB1 | | Q1CMD4 | | Q89AC2 | | P37652 | Q5P9S6 | P64036 | | Q19V75 | |
| Q39YG6 | B2JIH3 | | B5E2M6 | | P06881 | | Q0PXV9 | A1KJ22 | A6WP69 | | Q9XQQ6 | |
| Q5HAT4 | A7MN03 | | B1VYV9 | | P48401 | | A3Q7S1 | Q9LS23 | Q7UZX1 | | Q9FG87 | |
| B0TA56 | Q938C9 | | Q5AY82 | | Q7MUW6 | | Q7V007 | P79242 | Q87E75 | | Q5E888 | |
| Q9D5Z7 | O40977 | | Q9VTE0 | | A5I427 | | Q6XIP0 | A5F633 | A8I3Y9 | | Q9F7A2 | |
| Q81U58 | P01391 | | Q58178 | | Q83QC3 | | A9R2J3 | P04482 | P0AFD6 | | P53510 | |
| P0C5E7 | Q5L6X6 | | Q9LU02 | | P51448 | | P86231 | B0K9Q0 | P0A2E7 | | P9WJE2 | |
| Q7VDY0 | A7FCT8 | | Q8Y5S0 | | A7ZX05 | | A9B421 | Q7NRU2 | A5A3E0 | | Q9FRV1 | |
| Q2J3I3 | B7N839 | | Q4UMT4 | | Q5X1B5 | | Q9LVC9 | A8GB16 | Q2VEE1 | | Q8E9U9 | |
| A2QQV6 | Q2EEW1 | | A1CFS2 | | B7LXM2 | | A6MMP0 | O51886 | Q8EK52 | | Q8N1C3 | |
| Q2NZ91 | B5E2A0 | | Q8T6J1 | | A5GN79 | | Q7LZQ3 | A8ESV8 | Q8KAW3 | | Q9JHW1 | |
| Q04CM2 | Q8YQX7 | | Q4FS74 | | Q1CFI6 | | B4TQR9 | B8DWJ5 | B7M755 | | B1JGZ6 | |
| O74374 | Q5EA40 | | A3N0Z0 | | B0U3S8 | | Q1H4L3 | Q20765 | A9WY20 | | P35195 | |
| A4QLS2 | B8GV58 | | Q46IT3 | | Q39KH4 | | B7LEY4 | A5UL38 | Q9H2U2 | | Q5X0P2 | |
| A0QH68 | Q7SC98 | | P11705 | | Q2FZ92 | | P46535 | P42195 | Q01549 | | B8J861 | |
| P44595 | B0TL76 | | Q9DA01 | | B7NVR1 | | B9JH26 | Q59835 | P0C5W5 | | Q2NXC7 | |
| B9K8E3 | Q91FA3 | | Q88XZ2 | | Q45410 | | P9WJY2 | Q8CMX3 | A6W393 | | Q2J730 | |
| Q2JUV4 | Q1GBJ0 | | Q2RJW1 | | P00438 | | P97346 | Q13DP1 | B3QM10 | | A1SLS4 | |
| Q4USQ8 | Q924C6 | | A7NAX2 | | B1XEA2 | | A1RRK1 | Q1LUI2 | B8CVD4 | | Q97UA2 | |
| Q6G799 | A6QD47 | | B9E056 | | B4T708 | | A9HYV0 | Q89AF5 | Q53207 | | Q72R95 | |
| C5BGL0 | C4LCC5 | | O01840 | | Q9KLY7 | | Q9D5Z5 | P61218 | Q7XJK5 | | Q95QW4 | |
| Q9V0Y5 | Q67878 | | B4R8L6 | | A3M3G7 | | A7TJY9 | P11440 | P51945 | | P67274 | |
| P0A9B2 | Q13XV3 | | O55655 | | P0C044 | | P49871 | Q8HXX8 | Q1QSI0 | | O75096 | |
| B9KE24 | Q9Z806 | | Q4VUF1 | | A2RH21 | | Q8H1T3 | Q0WUU6 | A6QG68 | | B8IYH7 | |
| B1LLS6 | Q54I57 | | P56464 | | Q4FPK6 | | Q8D3Z2 | Q5N7A7 | Q6GZT6 | | P21081 | |
| B9KYX4 | B6JGU7 | | P60405 | | Q863C3 | | Q0TRH0 | C6C184 | Q75FF2 | | Q74AX4 | |
| Q5M0J7 | C3N7Q8 | | A2SB75 | | B2RH13 | | P19502 | B5EPM5 | Q1RE41 | | B9MR74 | |
| Q83MZ5 | Q9Z968 | | Q89AR8 | | P34422 | | Q6ND09 | Q2YUM5 | Q6B8M4 | | Q46ID3 | |
| C3N5V3 | Q02YG5 | | Q84N28 | | P51000 | | Q2YWG0 | Q98EE0 | Q1ZXE7 | | Q8Y032 | |
| Q9M4C4 | Q6KF81 | | Q5L8D7 | | O94693 | | B9IU58 | Q7Y7N3 | Q75B89 | | Q9TTJ7 | |
| P0DD54 | P0A1H3 | | Q70XW4 | | A4WSH9 | | A3M7Q1 | Q10979 | B1X4K3 | | Q08677 | |
| P24306 | Q318U1 | | A5I4L0 | | Q8EQB3 | | B2VK70 | A5VAK5 | B7UMH1 | | P0C494 | |
| P0A746 | A4QJG9 | | C1KYQ0 | | Q9CMZ2 | | Q8Z543 | Q821W4 | P35871 | | Q2FKP8 | |
| Q12C18 | Q5ACM9 | | Q8WI00 | | Q9ER10 | | Q04W49 | Q33C30 | Q8P3D7 | | Q0II41 | |
| Q8K9G2 | A7TFI4 | | A1KGK1 | | Q1H141 | | A0ALW6 | Q58445 | B3RNR8 | | Q9AGU4 | |
| Q5GTR3 | P39739 | | Q6N6L0 | | P34234 | | A1TJS8 | B7JKD5 | O88551 | | Q01FJ6 | |
| P40382 | B2UQY6 | | Q8L6J5 | | Q9C5E7 | | Q9PNI5 | Q05981 | B7UPX7 | | Q1CK94 | |
| A6M934 | Q9AMJ8 | | C1DCC2 | | B5F750 | | Q57425 | A8GKH8 | B4U516 | | A5IGB8 | |
| D1ZG64 | P48519 | | Q9PE38 | | B1LBK1 | | Q5HGV0 | E1SSZ4 | P27602 | | A2CCX2 | |
| P0A2I7 | Q3AUB6 | | P63861 | | A5UG34 | | P9WJB4 | Q5HF23 | P25272 | | P61110 | |
| A9WH99 | Q9RPP2 | | Q9MAL0 | | Q66G37 | | P33969 | A5I524 | B8HV20 | | Q88DV0 | |
| Q9WX71 | Q47Z14 | | O62677 | | Q8J1L4 | | P46222 | Q608S6 | P16303 | | A5FQX9 | |
| Q63RP8 | B1JFJ3 | | Q0HZR6 | | A8ERX9 | | A1VTF7 | B1LJ30 | A9MHL3 | | Q4FNW1 | |
| Q7Z7H3 | B2UUW4 | | B5Z3K2 | | A0LIP0 | | Q05029 | Q9C7F7 | Q13418 | | Q28GQ3 | |
| Q3JV86 | O24413 | | B1YKR5 | | Q8CXK5 | | Q8YBH3 | A0RI39 | Q15329 | | O84215 | |
| Q47HI2 | B8I578 | | B2IS39 | | A8GXC3 | | Q6YGZ4 | B4UBA1 | A1EA46 | | Q31W63 | |
| B2V7M3 | Q3Z9H9 | | Q14K36 | | Q0K769 | | A4QLN2 | A9AAZ2 | Q6DKI7 | | C5A241 | |
| Q755C8 | P50478 | | Q13VD0 | | Q07053 | | P18664 | B1VZ57 | Q64437 | | Q9LV10 | |
| P17804 | Q889W1 | | Q8PNS5 | | Q7V1I7 | | Q3M6L2 | Q3IMX5 | P34560 | | Q6N535 | |
| Q5L548 | Q81JW0 | | P08478 | | C6A1R5 | | Q88V81 | Q72NG9 | Q99K01 | | B7H299 | |
| P33860 | Q0VCG9 | | B0KRA9 | | Q09120 | | Q5PLI6 | P11944 | B5RR18 | | Q8CWC7 | |
| Q6FZC5 | B1P1C0 | | B1VG49 | | Q9UN72 | | P0A2R4 | Q0TMI3 | Q9BMK3 | | Q8FZP2 | |
| Q3IJH7 | A9BNK6 | | Q7NYC4 | | P9WG38 | | Q7WJ91 | Q037T6 | C5A286 | | Q3YSP8 | |
| A0A383 | P17474 | | Q81LS2 | | A7Z5B2 | | A9R395 | P51496 | B9MLZ0 | | Q5P322 | |
| P06368 | O80641 | | O95398 | | Q54QF4 | | P68267 | Q5HF39 | B0V5N7 | | Q6GFV0 | |
| Q6B8R2 | Q28113 | | C4KJM8 | | B0BQB0 | | C1CP93 | Q48A63 | B0S1N1 | | Q17Y14 | |
| Q6CZY0 | Q9RV79 | | A2XSY1 | | Q5RCA3 | | Q9FNY2 | A7FU83 | A7Z0P8 | | Q890X1 | |
| A2QAU8 | Q0ZJ18 | | Q05785 | | Q9I9B9 | | Q91PQ2 | P0A7R5 | A0K2H2 | | D2XV59 | |
| A0JMU8 | Q6GHW1 | | A4XI37 | | B4RD56 | | B3PDH6 | Q8CGM2 | P80301 | | Q3MKY2 | |
| P19423 | Q2HJD7 | | A6WNG2 | | A2SCG5 | | Q8WTR2 | B7JIE1 | P69684 | | Q8CPH7 | |
| Q319D8 | Q8R1B8 | | Q6FZS3 | | Q47R88 | | Q6LVB4 | Q09327 | Q0SNF0 | | A7ZY37 | |
| P61787 | O73700 | | B3QN57 | | Q9P7J4 | | A4SVI8 | Q4QK60 | P37082 | | Q6GV10 | |
| Q5E006 | Q6FKQ6 | | Q9Z6K9 | | Q8PML7 | | Q9KQ15 | B1J085 | P0A1Z8 | | Q5XDN3 | |
| A8M548 | O68986 | | Q754V0 | | Q6AXQ9 | | Q9P4E7 | Q7QAJ2 | B7MXZ7 | | P05937 | |
| B8D9K7 | B1H1F9 | | A7E8H8 | | A7FZ54 | | B2T612 | Q9H0X4 | O26130 | | C4XLY0 | |
| C5A1N3 | B2LMN3 | | Q9TF93 | | P44861 | | Q97EH2 | A5U0A5 | A7FIG1 | | P47755 | |
| Q3ZCV2 | P0AA15 | | P01029 | | A8AYK5 | | A5VD53 | B1N002 | P60452 | | Q9AI34 | |
| B7N7S4 | Q92BB9 | | A5VLJ3 | | B0BSU6 | | Q5P340 | Q14GJ6 | A3P7Z0 | | P15153 | |
| Q9BUT1 | Q74N73 | | P0AA12 | | Q8MIQ9 | | B8FT58 | Q6FCR9 | Q1XGU9 | | P22887 | |
| Q30SR5 | A4XF35 | | Q4ZX09 | | Q64PZ2 | | Q9FLG2 | Q33820 | Q3MA60 | | Q46FR7 | |
| Q829P9 | Q6DIP3 | | Q6ENU0 | | A5WM93 | | B1JBA9 | O59303 | Q9MI64 | | Q5ZRJ5 | |
| Q3IBU5 | Q9WY96 | | P73296 | | Q9CQE6 | | A0M537 | P02704 | Q0V9W6 | | Q4A0G1 | |
| P19624 | Q0RDP7 | | P17417 | | A3NA56 | | A6WZW3 | P36625 | Q6G084 | | B4UIV7 | |
| Q0ZIZ8 | A5I0A5 | | Q5GXV3 | | Q6A9Q5 | | Q95342 | Q9CLG7 | Q8WHM8 | | P66274 | |
| P0C9M3 | C4ZY31 | | Q9HMN0 | | Q9KTJ5 | | Q06154 | B6JLD2 | A5U121 | | Q8G1M4 | |
| B0V6X3 | O76971 | | C0MBT1 | | Q6UX41 | | Q90WJ3 | Q8SPY6 | Q5NVS4 | | P0CN30 | |
| Q7N8B1 | A1UBY6 | | O63930 | | P60181 | | Q5RFK9 | B7IFU7 | C4L810 | | A5D1I3 | |
| Q9DHT0 | Q92ZT0 | | Q8T112 | | Q3JRM8 | | A9WI34 | Q59ZZ6 | Q5YNB0 | | A9VMG0 | |
| Q3SAT7 | C0JAQ5 | | Q6CZT4 | | P44813 | | Q2KIV9 | A4VNP3 | B2RL41 | | P58332 | |
| B8G461 | Q2IH91 | | A4QDL6 | | Q8K3C0 | | C1FNH5 | A8G1F0 | P51262 | | A9BDG0 | |
| B0U3R3 | Q9H9F9 | | B7KIR6 | | P0ABJ7 | | Q57RH2 | Q890P1 | B9M914 | | Q54WS5 | |
| A7H0Z4 | Q2QWW7 | | P00653 | | P0C7A2 | | O83759 | Q9BZG8 | Q3AGT1 | | P9WHT3 | |
| Q1JKP7 | P24717 | | P01768 | | P34854 | | Q6GG27 | P38609 | Q4QQQ4 | | Q6GZR9 | |
| B2IS66 | C4Y206 | | Q3SRA8 | | Q80Y50 | | Q7W2F6 | B2TU31 | Q0I560 | | Q1QH30 | |
| A3D1R9 | Q0SQD3 | | C3MR78 | | A9R076 | | Q9PK26 | B0B7N3 | Q046B3 | | Q7VWE7 | |
| Q8G2M7 | B7L8T9 | | A7H664 | | A1JT84 | | A2S8K4 | C1A0X5 | P20779 | | Q87YC6 | |
| Q97H50 | P57472 | | A4JCC5 | | P30080 | | P19627 | P42475 | Q9H633 | | Q1JPA0 | |
| C0PZW7 | A4TMT8 | | B7V208 | | B1IBH4 | | A5U1K8 | Q556Q3 | P9WNR5 | | P69902 | |
| Q3J2Y1 | C1ER80 | | Q1GLQ5 | | B7UIK6 | | A8LB01 | P86424 | P30277 | | Q8BU03 | |
| P27403 | Q4R4J0 | | Q0VAX3 | | Q85435 | | A1JPA5 | Q4ZJY8 | P59205 | | Q9Z4Z7 | |
| Q67185 | P9WJB2 | | Q1QVG9 | | Q7YRH1 | | B6JN18 | A6VIS0 | Q3SWY3 | | P10251 | |
| Q13TF9 | Q0AE32 | | Q05975 | | O59856 | | Q5Z2U2 | Q46HH1 | Q7W566 | | A6U1N8 | |
| P04809 | B2VJ42 | | Q1QCP8 | | B8FT17 | | P0A3Q6 | O94345 | Q9ZE21 | | Q7VQM8 | |
| Q4P5F5 | Q0I0B1 | | Q0APT3 | | Q5QM25 | | P33724 | Q3Y5G8 | Q9VSD7 | | A0Q528 | |
| Q4K3S0 | Q3BX06 | | B5E4T0 | | A1CBP8 | | Q9ZM44 | Q7W050 | Q7MBC1 | | B1YT08 | |
| Q53SF7 | Q3IF25 | | D4D675 | | A7N9T1 | | Q2T1C2 | B5ZAY6 | P00657 | | Q7VW29 | |
| B7NRC9 | P66669 | | B3CM66 | | Q9HXQ0 | | A7ZWJ6 | P14029 | Q62J26 | | A1V8S5 | |
| A3EZ55 | Q05610 | | Q8YPJ1 | | B4EUG3 | | A2T378 | Q5PHT4 | B0RT37 | | P52723 | |
| Q5LW48 | Q8WWZ3 | | Q85X54 | | Q8U374 | | A8L2B6 | Q6LVB1 | Q1CUY2 | | P05767 | |
| B5Y8M4 | B6I234 | | Q9HFY6 | | P20215 | | Q21QP2 | Q3JH97 | A2S3Q4 | | B1KYH7 | |
| A7MN01 | Q6LXD4 | | A6W969 | | B3EUK8 | | P0C9M9 | Q8XGA0 | C3L0M1 | | Q33BZ1 | |
| P17005 | P9WIY0 | | P29965 | | A6Q581 | | P9WGY3 | B9MRK2 | P49849 | | C1AGR4 | |
| Q7WII7 | Q01444 | | B5F7I4 | | Q9I4Z2 | | Q8VD83 | A5VA82 | B7M133 | | B4R9R7 | |
| P01261 | Q12UP9 | | Q64VP0 | | Q9FY50 | | Q959V6 | B7N7R3 | C3K469 | | Q8PW06 | |
| P01089 | Q827R5 | | Q9UJ99 | | B4TQC3 | | B1ZG99 | Q9ZCQ4 | Q2J6Y2 | | Q10Q46 | |
| P66686 | Q21TL4 | | Q39596 | | Q18CA2 | | Q609J2 | Q9MUL7 | C6DCI7 | | Q3J7Y0 | |
| P11894 | Q8B9Q8 | | P38221 | | Q0P3M0 | | B5BA35 | Q2RN76 | Q2W4C6 | | Q9W1U4 | |
| Q54IU8 | Q81K04 | | P51728 | | Q9ZV52 | | P9WQ37 | Q03298 | Q1J0P9 | | Q69Z61 | |
| B3CMP5 | P0C493 | | Q0W2J4 | | Q9HAT0 | | B2VG97 | Q2EEV8 | Q1C9H9 | | P12442 | |
| Q62HE6 | A2BT67 | | Q5E9K8 | | Q817Q2 | | B8HL79 | Q04443 | O13967 | | C5CXH7 | |
| P18870 | B7MQJ1 | | B3H5Q1 | | C0RH46 | | O74854 | Q3T9M1 | A2SAW7 | | B2S3S0 | |
| Q01837 | B2S663 | | Q3MEN8 | | Q8ZEB3 | | Q92564 | B2T6J4 | P49918 | | B5E3G0 | |
| Q4QLL2 | Q2HJ93 | | Q9RH40 | | A8EXT7 | | B3DTC2 | B7V4D9 | P39760 | | Q39Q56 | |
| Q2FDI7 | A1A947 | | Q0TNE1 | | Q9WYM8 | | O26115 | Q09704 | Q03YS1 | | B5ZVU9 | |
| B5ZCB8 | Q8PNR1 | | B5XSW2 | | Q9L7Z3 | | P0CN41 | B8J1X3 | Q0I2N3 | | B0BS03 | |
| Q5YZY9 | A1TN72 | | Q730K1 | | P48854 | | B2XWM8 | C3N8M1 | A5F0D5 | | P26325 | |
| Q5WV31 | P9WHE3 | | Q6IN84 | | A4XWP8 | | O53551 | A5IHP0 | Q9UTE6 | | P98110 | |
| B2K518 | Q3Z7C7 | | Q82681 | | Q71WQ4 | | Q13TZ0 | Q9XWE9 | Q4AAU5 | | A5ISS7 | |
| Q17X37 | Q9JFM9 | | Q169E2 | | Q493M0 | | Q5PAQ2 | Q3JCJ6 | Q7MNG3 | | Q8GU86 | |
| Q9BYT8 | A4SFZ9 | | Q5RJV0 | | C6A429 | | P0A6R0 | B0KK69 | A6TI00 | | I4EPA3 | |
| A1VJY2 | Q68CI2 | | Q9S9P2 | | P51566 | | Q1XDF0 | Q7N8V4 | A7E3Q8 | | B9MKF2 | |
| Q8TS37 | Q7MP92 | | Q9K9M8 | | P68921 | | Q2SBD7 | B5RDS6 | B2RLZ1 | | P0A9R6 | |
| Q6UXD7 | B3E0J9 | | G1UII1 | | P59241 | | Q24UZ7 | A7FFK5 | P96037 | | A9BF03 | |
| A5WG49 | Q3MHJ5 | | A0RMQ6 | | B1XW69 | | A9R805 | A0M597 | B1XN32 | | Q85FV0 | |
| Q9ZDW1 | Q8FA32 | | Q2YNB0 | | P61199 | | Q67S99 | P50685 | P38738 | | Q197D0 | |
| D3Z1Q2 | A8F9A6 | | P39712 | | Q66FF3 | | Q7MYG2 | Q06J36 | B2S532 | | B1IC08 | |
| A9R664 | Q5FFJ4 | | Q39I87 | | Q6ARX9 | | A2BPX4 | B7NLL0 | Q9VAD6 | | Q1ACN5 | |
| Q8NKW9 | O35240 | | Q088A4 | | Q746G0 | | Q28IM6 | Q5HIH2 | Q49WI3 | | B4RJY6 | |
| A8G1C4 | A8ANZ1 | | P17353 | | Q7WRC9 | | Q76V03 | B5RLV1 | P98054 | | O18971 | |
| A3DA73 | Q9MYS6 | | P41851 | | B0VLZ5 | | B2GKH0 | B1ZLJ9 | Q5NQH8 | | Q0V8B7 | |
| C1B7T0 | Q3T0T0 | | Q5REL3 | | Q2SU16 | | C1DCN2 | P09502 | B2A4Q1 | | Q60HE2 | |
| Q8KA37 | A1WV96 | | B5X392 | | A8MZ97 | | B4TY04 | B5YIQ7 | Q7MTL8 | | Q29555 | |
| P34092 | Q9FLN0 | | O59133 | | Q2SL55 | | A5V280 | Q3JMZ6 | Q4URT6 | | Q92C79 | |
| Q06912 | A8HYU5 | | P56724 | | Q70XZ1 | | Q2YDK0 | P91252 | C5BMA0 | | P0DJT6 | |
| Q1C5V2 | C5BAU7 | | Q4ZZG6 | | P40607 | | B3CN50 | Q13V50 | O13614 | | Q7ZV00 | |
| Q1CCJ6 | Q8NN57 | | B3EUF2 | | P30597 | | Q8GDQ6 | A6L685 | C3P7V9 | | Q85FJ0 | |
| Q5ZZ86 | Q73F91 | | Q99P21 | | Q6G2Q5 | | Q7NIT2 | Q3K1Q9 | Q9WUB4 | | Q6DEV3 | |
| P11510 | Q5L562 | | A1VUV1 | | P0A479 | | A1RNU4 | B9JVD6 | Q48VQ0 | | Q8ZT92 | |
| D7STK2 | P17697 | | Q9CPA2 | | A4SUW4 | | B7MYJ7 | B0SS68 | Q24T28 | | A3MM56 | |
| B7MCT5 | Q0TIY5 | | Q92338 | | Q1RFX9 | | Q9PET2 | Q6P1H6 | B2UXE0 | | Q2JSU8 | |
| Q4VZN0 | A5I5I5 | | C4Z2U6 | | B3PWQ6 | | P81425 | Q21VT3 | A0K3N5 | | P9WGY8 | |
| Q8KBD8 | B9JX30 | | C3L763 | | P40390 | | P30161 | Q0WPW5 | P37474 | | Q923G2 | |
| O74213 | P02768 | | B7LWK5 | | Q00052 | | Q2KUK5 | P30753 | Q32B42 | | B2S9V1 | |
| A8CEN3 | Q5HWG0 | | A5W1E8 | | Q9KTX3 | | Q7VZQ3 | A6VGQ8 | Q8GXN2 | | P61309 | |
| O59715 | B6I8Y4 | | C5B8X6 | | B3PMN2 | | Q2VL78 | Q0J4D4 | P58289 | | A2RC35 | |
| A3E4D8 | P33901 | | P0C138 | | A1KHS4 | | P15471 | B4UT09 | Q57F29 | | Q6UDH4 | |
| Q9S7H4 | B1JTF0 | | Q330K2 | | O70570 | | Q8YHP8 | A3NDW3 | B2TTX9 | | Q5YZ59 | |
| B8DTW7 | A0RP13 | | Q11DD6 | | C0MGL4 | | P60193 | Q54MT0 | A8A6M7 | | P63823 | |
| Q14JC1 | O78936 | | A8XKV0 | | A6QIW2 | | Q9STF1 | P80544 | P03596 | | O52194 | |
| Q3SMH1 | Q4W382 | | A9MVI4 | | A8B5P0 | | Q9WUX5 | Q7WI07 | Q8H112 | | Q2ILC5 | |
| C1CEU3 | Q606F3 | | Q6GBY7 | | Q0MQJ4 | | Q62HK4 | B0KJ96 | Q6CPX6 | | P47860 | |
| B4H581 | Q8EM65 | | P07458 | | A1KS33 | | Q3APJ1 | P21603 | A0PJX0 | | B2V652 | |
| P21660 | Q9UK53 | | Q83LU4 | | Q04152 | | Q39ZA5 | Q7BZ80 | A6H4Q5 | | P55993 | |
| P32529 | A0LRE5 | | P35984 | | Q97IC9 | | A1VRC8 | A6MM31 | Q6GZS7 | | P00652 | |
| P01763 | B5EZJ2 | | B4TJ60 | | B4SA43 | | Q88VE0 | P24709 | C0QC86 | | Q42381 | |
| B2RMJ1 | B3PIU8 | | A5U6V9 | | Q4WZY1 | | Q9KTX1 | Q0I1D2 | Q5SYE7 | | P82174 | |
| B7IN19 | Q08031 | | A5UHJ6 | | Q5R4I8 | | Q5ZRU0 | Q3SS37 | Q8A466 | | Q87AB7 | |
| A1JKC3 | Q895K8 | | P27039 | | P0C9E7 | | P27493 | A4YF02 | Q9FFF4 | | A1S1P8 | |
| Q8EHK0 | Q02XA4 | | B2TVQ7 | | P12487 | | C3N7I8 | Q9FN08 | Q9NQW8 | | B1LNT8 | |
| Q3E703 | B5EXE7 | | A6VM01 | | B5YZ00 | | B0C899 | Q1J733 | Q5F7E6 | | B5Z8L0 | |
| Q5CD96 | D6C4L6 | | Q8TXI5 | | A2SGQ6 | | B7NLI7 | A6VCK3 | A7X5G0 | | Q788J2 | |
| B9DUK3 | A7ZU50 | | B8CKI4 | | Q09MC9 | | Q6DEG4 | B5XJY9 | A0Q6F0 | | C5A1H2 | |
| Q8PAD2 | B1L8T8 | | Q0HV48 | | Q8TZW4 | | C3K6E1 | Q8EH71 | Q9DAT5 | | A5CRQ9 | |
| A6U6K3 | A8G7E8 | | Q96S96 | | Q02T64 | | Q0BKC0 | B8GQV7 | Q9KNH4 | | A0LT89 | |
| Q83BL5 | B4T3P6 | | Q1DCX5 | | B7IH25 | | Q9N5L4 | Q1IXW9 | Q7NH24 | | Q6EN83 | |
| Q9RDE5 | Q07E47 | | Q332V6 | | P0ADG9 | | B4T6V0 | Q475R4 | P28418 | | Q32RL5 | |
| Q4VNZ6 | Q9ZIX6 | | Q88M04 | | Q9ZS34 | | Q11HB4 | B0YPQ8 | A7NR58 | | P95713 | |
| A3KQA5 | C1L2M0 | | Q9KWG3 | | P30055 | | O58058 | A7H651 | Q85B65 | | Q6CJI9 | |
| Q0AIH8 | Q2S0M9 | | A9MYJ6 | | C5CQ74 | | P37419 | P0A2B8 | P0CV94 | | Q6YXM4 | |
| A6BM12 | Q49SP8 | | A3CP33 | | B3QQI2 | | O59619 | B4UBB0 | Q2Q5Y8 | | B9JDB9 | |
| Q9YGP5 | Q5F3A2 | | B0TD39 | | A1K3U6 | | Q5ABZ2 | Q2JJM6 | A5F3N4 | | Q64896 | |
| Q93KF4 | Q4ZPM1 | | B7K762 | | P53502 | | Q9KVE0 | A4XPS6 | Q863H3 | | Q9TI97 | |
| A4IZA2 | Q93YW7 | | Q4V7A9 | | B2RC85 | | Q318N6 | P40616 | B5QTH9 | | Q96TA0 | |
| A0RBW0 | Q0I4M3 | | A4XR61 | | A4QJS3 | | A4SVV4 | A4YT30 | Q2JHS3 | | O88379 | |
| Q8EHC4 | Q88P52 | | A8F1N2 | | P0C1U3 | | B5BGA7 | Q5SCX5 | P66124 | | Q8K4Y7 | |
| C0QRW6 | Q3HRN9 | | Q2G8H1 | | Q9Z7E8 | | Q8JHV9 | Q6PIE5 | A4QGQ6 | | P0C5V0 | |
| B2UCD6 | A7HB50 | | Q2KIR4 | | Q88L02 | | A1BEZ3 | A0Q8R8 | Q8D8Q9 | | B8I2Q1 | |
| Q866F1 | Q13496 | | A7ZVD3 | | C3K259 | | B2RL46 | Q8G401 | O88984 | | P23250 | |
| B0TDI1 | A4XDK4 | | Q8CFK6 | | Q7MPI6 | | B1XB03 | B2IPA0 | B8ZPY7 | | B9IZL0 | |
| Q4FR30 | A9VKN0 | | Q2NQF9 | | P49332 | | B2U8D8 | Q5UXN1 | Q5QVE5 | | Q776A6 | |
| O97401 | Q48VT4 | | Q8GUQ5 | | P62200 | | Q6FYD4 | A5UJW5 | Q3T062 | | P52610 | |
| Q0S3H4 | A5U089 | | Q92I26 | |  | |  |  |  | |  | |
